# Exploring Zinc(II) Coordination Chemistry with Picolinate and Amino Alcohols: Toward New Antibacterial Agents

**DOI:** 10.1021/acsomega.6c02474

**Published:** 2026-05-25

**Authors:** Barbara Modec, Nina Podjed Rihtaršič, Joaquín López-Serrano, Martina Hrast Rambaher, Majda Golob

**Affiliations:** a Faculty of Chemistry and Chemical Technology, 37663 University of Ljubljana, Večna pot 113, Ljubljana 1000, Slovenia; b Instituto de Investigaciones Químicas (IIQ), Departamento de Química Inorgánica and Centro de Innovación en Química Avanzada (ORFEO−CINQA), Consejo Superior de Investigaciones Científicas (CSIC) and Universidad de Sevilla, Avenida Américo Vespucio 49, Sevilla 41092, Spain; c Faculty of Pharmacy, 37663 University of Ljubljana, Aškerčeva 7, Ljubljana 1000, Slovenia; d Institute of Microbiology and Parasitology, Veterinary Faculty, University of Ljubljana, Gerbičeva 60, Ljubljana 1000, Slovenia

## Abstract

Zinc­(II) coordination chemistry with picolinate (abbreviated as pic^–^, an anion of pyridine-2-carboxylic acid) and a series of β-amino alcohols (2-aminoethanol, 2-methylaminoethanol, 2-ethylaminoethanol, 2-dimethylaminoethanol, 2-amino-1-propanol, 1-amino-2-butanol, and 1-amino-2-methyl-2-propanol) was systematically investigated. Reaction systems typically yielded complexes with the general composition [Zn­(pic)_2_(amino alcohol)] *via* a straightforward synthetic route. Zinc­(II) adopts an octahedral coordination environment consisting of two *N*,*O*-bidentate chelating picolinates and one amino alcohol, likewise coordinated in a chelating manner through amino and hydroxyl groups. As no *fac* isomers were isolated, the relative stability of *mer* and *fac* isomers of the 2-methylaminoethanol complex was evaluated by DFT calculations, which supported a preference for the *mer* geometry. Interestingly, the 1-amino-2-methyl-2-propanol system also produced an ionic species, [Zn­(pic)_3_]^−^, which crystallized as a salt with protonated amino alcohol. All novel compounds were characterized using infrared and ^1^H NMR spectroscopy, elemental analysis, mass spectrometry, and single-crystal X-ray diffraction. Selected *tris-chelates* displayed moderate antibacterial activity against *S. epidermidis*, with MIC values ranging from 32 to 64 μg/mL, whereas picolinic acid alone was inactive.

## Graphical abstract

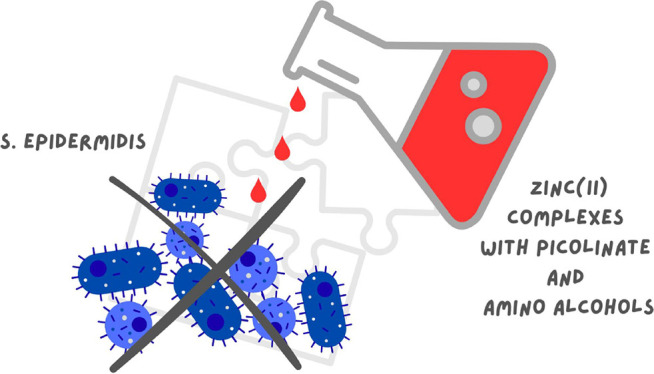

## Introduction

Infections caused by bacteria are one of the most common causes of death worldwide. A recent study estimated that 13.7 million infection-related deaths occurred in 2019.^1^ Following Fleming’s discovery of penicillin, the first true antibiotic, millions of lives were saved; however, only a few years after its widespread use, more than 50% of bacterial strains had already developed resistance.^2^ Unfortunately, this is still the case today, as an increasing number of pathogens acquired resistance to existing treatments. Part of the solution is to better understand mechanisms of bacterial resistance.^3^ As an alternative, metal complexes are also being investigated for their antimicrobial properties.^4–6^ In many instances, the metal complexes exhibit greater activity than the ligands alone.^7^ Metal complexes offer unique modes of action such as exchange or release of ligands, redox reactions, and generation of reactive oxygen species. Due to the Lewis acidity of metal ions, they can interact with electron-rich molecules such as DNA and proteins. They can exist in different coordination environments and geometries. This is important as three-dimensionality has been associated with improved selectivity.^8^ Among the metal ions found in bioactive metal complexes, zinc­(II) holds a particularly prominent position. Its biological importance arises from its essential role in hundreds of enzymes and regulatory proteins, where it contributes to both catalytic activity and structural stability.^9^ Its unique chemical properties make zinc suitable for its many biological roles. Owing to its *d* ^10^ electron configuration, zinc­(II) is difficult to oxidize or reduce.^10^ In living organisms, zinc exists in only one oxidation state, namely, +2. Furthermore, its spherical electron configuration leads to minimal ligand-field stabilization effects, enabling various coordination environments: from tetrahedral through trigonal-bipyramidal or square-pyramidal to octahedral.^11^ By being of borderline hardness, zinc­(II) readily coordinates *N*-, *O*-, and *S*-donor ligands. Consequently, numerous zinc­(II) complexes have been extensively investigated for their physiological merits,^12^ including antibacterial activity.^13–17^ Reported mechanisms involve multiple inhibitory effects on the bacterial metabolism such as glycolysis, interference with polysaccharide synthesis and, suppression of glucosyltransferase production.^18^ Complexation of zinc­(II) with suitable multifunctional organic ligands is believed to improve its bioavailability and strengthen its antimicrobial efficacy.

As a follow-up of our research on zinc­(II) complexes, we investigated the coordination behavior of zinc­(II) toward picolinate and selected amino alcohols. Picolinic acid (IUPAC name: pyridine-2-carboxylic acid, abbreviated as picH) consists of a pyridine ring with carboxyl group attached to its *ortho* position (Scheme 1). In its deprotonated form, it typically binds to transition metal ions in an *N*,*O*-bidentate chelating manner.^19^ Picolinic acid has already been introduced in the coordination chemistry of zinc­(II), as exemplified by several simple mononuclear complexes such as [Zn­(picH)­(pic)­Cl], [Zn­(picH)­(pic)­Br], *cis*-[Zn­(pic)_2_(H_2_O)_2_]·0.5DMF, and *trans*-[Zn­(pic)_2_(H_2_O)_2_]·2H_2_O (where pic^–^ denotes a form with a COO^–^ group).^20–24^ Specifically, *trans*-[Zn­(pic)_2_(H_2_O)_2_]·2H_2_O and a series of zinc­(II) complexes with picolinate derivatives have been evaluated for insulinomimetic properties.^24,25^ In contrast to picolinate, which exhibits a well-defined and preferred binding mode toward metal ions, amino alcohols are known for their structural flexibility. Amino alcohols contain two functional groups, a hydroxyl group and an amine group, attached to an alkyl chain, and both can coordinate to transition metal ions. As a result, a variety of coordination modes are possible: either one or both donor groups may bind, and a single ligand can coordinate to one or multiple metal centers. The hydroxyl group often undergoes deprotonation, and the resulting anion typically binds in a chelating bridging fashion.^26^ The amino alcohols selected for this study, shown in Scheme 2, are all β-amino alcohols. Their general structure may be represented as R_2_N–C_β_–C_α_–OH, where R stands for H or alkyl. Despite the commercial availability of amino alcohols, their coordination chemistry toward zinc­(II) remains comparatively underexplored. Known zinc­(II) coordination compounds mostly feature polyfunctional amino alcohols such as diethanolamine,^27^ *N*-methyldiethanolamine,^28,29^ and triethanolamine.^29–32^ Reports of zinc­(II) complexes with simpler β-amino alcohols are less common and include 2-aminoethanol,^27,33^ 2-dimethylaminoethanol,^34,35^ and (*S*)-2-amino-2-phenylethan-1-ol.^36^ A report on the successful introduction of 2-methylaminoetanol and quinaldinate, as a representative of a ligand with a highly directing binding mode, comes also from our laboratory.^37^ In zinc­(II) coordination chemistry, β-amino alcohols typically bind in an *N*,*O*-bidentate chelating manner, forming a five-membered chelate ring.^19^ Only rarely, one functional group can be engaged in coordination, this being the amine group. The β-amino alcohols are known for various biological activities: some are used as antibacterial agents,^38^ active pharmaceutical ingredients such as HIV protease inhibitors,^39^ and antihypertensives.^40^ Thus, it comes as no surprise that the dinuclear zinc­(II) complex with (*S*)-2-amino-2-phenylethan-1-ol, one of the cited examples, showed anticancer activity.^36^

**Scheme 1:**
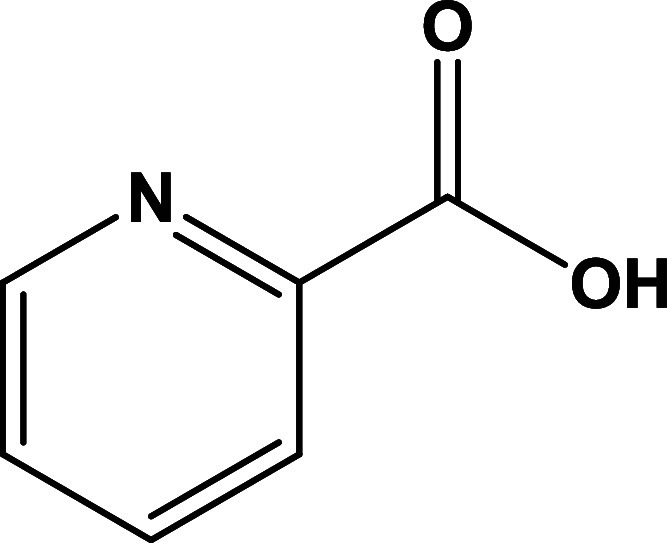
Molecular Structure of Picolinic Acid (picH)

**Scheme 2:**
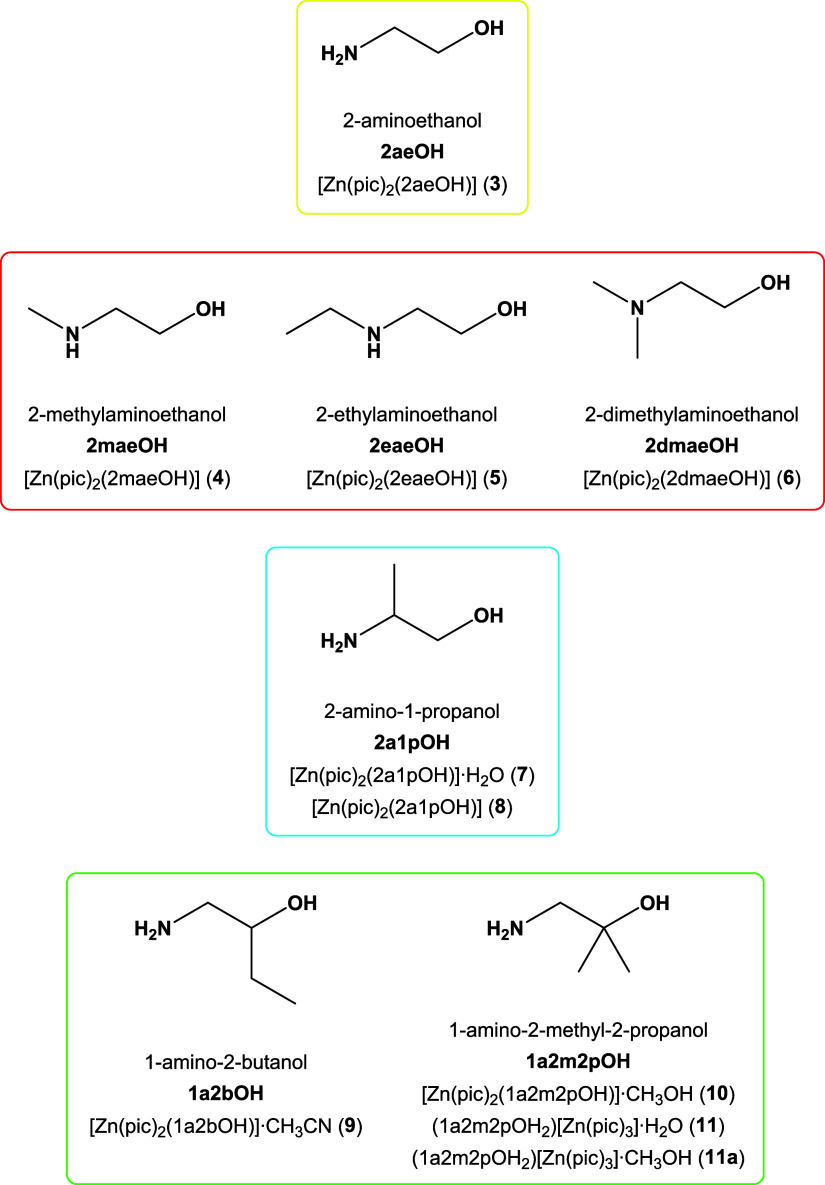
Amino Alcohols Used in This Study (Structural Formula, Name, and Abbreviation) and Zinc­(II) Compounds Obtained from Each System (Formula and Label)

Herein, we report on the synthesis, IR and ^1^H NMR spectroscopic characterization, and X-ray structures of a series of mononuclear zinc­(II) complexes with picolinate and amino alcohol ligands. With no exception, the novel complexes featured *mer* distribution of N_3_O_3_ donors. Thus, the stability of the *mer* and *fac* isomers of an exemplary complex was evaluated by theoretical DFT calculations. Selected compounds were also tested for their antibacterial activity against Gram-positive and -negative bacteria.

### Synthetic Considerations

For the synthesis of novel zinc­(II) complexes with picolinate and amino alcohols with the [Zn­(pic)_2_(amino alcohol)] composition, a straightforward synthetic procedure, as summarized in Scheme 3, was employed. First, zinc­(II) oxide was reacted with picolinic acid in a 1:2 mol ratio in methanol under reflux to produce a clear solution of the {Zn­(pic)_2_} precursor. Upon prolonged standing of this solution, small amounts of large, colorless crystals of *trans*-[Zn­(pic)_2_(CH_3_OH)_2_] (**1**) occasionally precipitated. When crystals of **1** were taken out from the solution, they underwent an almost instantaneous decomposition, which involved a loss of methanol ligands and the absorption of air moisture. Owing to their inherent instability, no further attempts were made to isolate **1** from the solution. In the second step, the {Zn­(pic)_2_} precursor solution was reacted with the amino alcohol under reflux. The heating was typically maintained for 3 h. Alternatively, the amino alcohol could be added at ambient temperature, in which case the reaction mixture was allowed to stand for 1 week prior to further treatment. The resulting solutions were then concentrated and treated with a small amount of nitrile, and finally, the precipitation of the corresponding [Zn­(pic)_2_(amino alcohol)] complex was induced by vapor diffusion of diethyl ether. This step was essential as no solids formed without diethyl ether. The procedure furnished crystals suitable for X-ray diffraction in moderate to good yields. The nitrile was identified through trial and error, and the one that ultimately produced the highest quality crystals was selected.

**Scheme 3:**
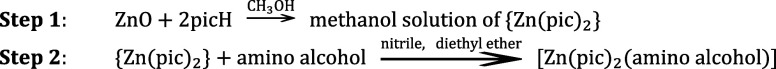
Synthesis of [Zn­(pic)_2_(amino alcohol)]

A series of amino alcohols were employed (Scheme 2). They differed in the alkyl substituents and their relative position with respect to the hydroxyl or amine groups. Three of these amino alcohols bear alkyl substituents on the amine nitrogen: 2-methylaminoethanol and 2-ethylaminoethanol, both secondary amines, and 2-dimethylaminoethanol, a tertiary amine. Considering the immediate chemical environment of the hydroxyl group, some can be classified as primary alcohols, with one example each of a secondary and a tertiary alcohol. Scheme 2 also lists zinc­(II) compounds obtained from each amino alcohol system. Some of the reaction systems merit further comments. Interestingly, the attempt at recrystallization of the 2-dimethylaminoethanol complex [Zn­(pic)_2_(2dmaeOH)] (**6**) from a mixture of methanol and propionitrile yielded *cis*-[Zn­(pic)_2_(H_2_O)_2_]·1/2CH_3_CH_2_CN (**2**). After standing in a sealed glass tube for 1 week, water apparently entered the system, replacing the amino alcohol ligand. Consistent with the donor arrangement in the starting complex, the *cis* isomer of [Zn­(pic)_2_(H_2_O)_2_] was obtained. This transformation highlights the known tendency of zinc­(II) to coordinate water molecules.^41^ In contrast, the reaction with 2-amino-1-propanol initially produced pure crystals of the hydrated form, [Zn­(pic)_2_(2a1pOH)]·H_2_O (**7**). On further standing of the reaction mixture, crystals of the anhydrous compound [Zn­(pic)_2_(2a1pOH)] (**8**) began to precipitate. This sequence of events could be attributed to small amounts of water inherently present in the sealed system. The hydrate forms first when sufficient amount of water is available, and only after its content decreases, and no additional water enters the system, does the anhydrous compound precipitate. The 1-amino-1-butanol system is also of interest as it produced crystals of two distinct morphologies: large prisms of [Zn­(pic)_2_(1a2bOH)]·CH_3_CN (**9**) and very fragile, needle-like crystals that grew in clusters that we labeled as **9a**. Due to their thinness and fragility, X-ray structure analysis of needle-like crystals was not feasible. The spectroscopic data, IR, and ^1^H NMR spectroscopy strongly suggest that these needle-like crystals are also [Zn­(pic)_2_(1a2bOH)] complex that contains acetonitrile solvent molecules. The 1-amino-2-methyl-2-propanol system is particularly noteworthy, yielding not only the very unstable methanol solvate [Zn­(pic)_2_(1a2m2pOH)]·CH_3_OH (**10**) but also a few crystals of the protonated amino alcohol salt (1a2m2pOH_2_)­[Zn­(pic)_3_]·H_2_O (**11**). Its formation is unexpected: with no extraneous source of picolinate, the stoichiometry of **11** implies a zinc­(II) species in solution with fewer than two picolinate ligands permetal ion. Moreover, the source of the acid responsible for protonating amino alcohol remains unknown. To perform a complete characterization of the protonated amino alcohol salt **11**, a rationamarized in the equation below, was attempted. A methanol solution of **10** was reacted with the excess of picolinic acid. Precipitation with diethyl ether, interestingly, afforded crystals of another solvate, (1a2m2pOH_2_)­[Zn­(pic)_3_]·CH_3_OH (**11a**).

$$\left[\mathrm{Zn}{\left(\mathrm{pic}\right)}_{2}\left(1a2m2\mathrm{pOH}\right)\right]+\mathrm{picH}\to \left(1a2m2{\mathrm{pOH}}_{2}\right)\left[\mathrm{Zn}{\left(\mathrm{pic}\right)}_{3}\right]$$

In summary, all systems produced zinc­(II) complexes containing two picolinate ligands and one amino alcohol ligand, each bound in a chelating bidentate manner. Notably, in some cases, the amino alcohol-to-zinc­(II) mole ratio exceeded 2, yet this had no influence on the nature of the isolated product. This observation supports the notion that [Zn­(pic)_2_(amino alcohol)] complexes are, in general, thermodynamically stable. Moreover, our results demonstrate that methyl, ethyl, and two methyl groups on the nitrogen of the amino alcohol did not introduce sufficient steric hindrance to impede coordination of the amine nitrogen to the zinc­(II) ion.

### Crystal Structures

*trans*-[Zn­(pic)_2_(CH_3_OH)_2_] (**1**) exhibits a *trans* arrangement of two *N*,*O*-bidentate chelating picolinates and two monodentate methanol ligands. The ORTEP drawing of **1** is shown in Figure 1. The N_2_O_4_ donor atoms provide a nearly octahedral coordination environment for the central metal ion. With the metal ion residing on the inversion center, one-half of the complex molecule belongs to the asymmetric unit. The zinc­(II)-to-picolinate bonds are shorter than those reported for *trans*-[Zn­(pic)_2_(H_2_O)_2_]·2H_2_O.^23^ In **1**, the lengths of the Zn–N and Zn–O bonds are 2.0825(13) and 2.0485(10), respectively, whereas the corresponding bonds in *trans*-[Zn­(pic)_2_(H_2_O)_2_]·2H_2_O amount to 2.1161(17) and 2.0755(13) Å. Compared to other zinc­(II) picolinate complexes, the picolinate ligand in **1** displays a rather large bite angle [the N–Zn–O angle], 80.88(4)°. The Zn–O­(methanol)­bond with the length of 2.2153(11) is longer than 2.170(2) Å, a bond observed for *trans*-[Zn­(quin)_2_(CH_3_OH)_2_].^42^

**Figure 1:**
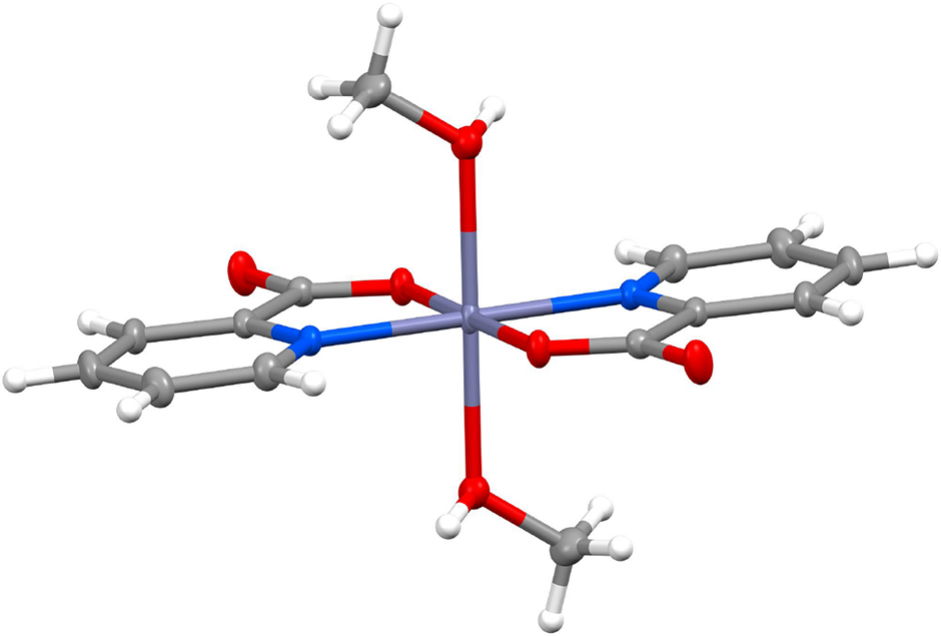
ORTEP drawing of *trans*-[Zn­(pic)_2_(CH_3_OH)_2_], a complex molecule in **1**, with thermal ellipsoids at the 50% probability level.

The complex molecule of *cis*-[Zn­(pic)_2_(H_2_O)_2_]·1/2CH_3_CH_2_CN (**2**) features a *cis* distribution of ligands (Figure 2). An analogous compound containing dimethylformamide in place of propionitrile has been reported previously.^22^ Notably, the dimethylformamide analogue is likewise a hemisolvate. Moreover, the two structures are isotypic and display very similar geometric parameters for the complex molecules (Table 1). In the *cis* isomer **2**, the zinc­(II)-to-picolinate bonds are slightly longer than those observed in *trans*-[Zn­(pic)_2_(H_2_O)_2_]·2H_2_O.^23^ In contrast, the Zn–O­(water) bond in **2** is approximately 0.08 Å shorter than in the *trans* isomer.

**Table 1:** Relevant Geometric Parameters [Å,°] of Selected Octahedral Zinc­(II) Complexes with Picolinate

	Zn–picolinate				
**compound**	Zn–N	Zn–O	picolinate bite angle	L^a^	Zn–L
*trans*-[Zn(pic)_2_(CH_3_OH)_2_] (**1**)	2.0825(13)	2.0485(10)	80.88(4)	CH_3_OH	2.2153(11)
*cis*-[Zn(pic)_2_(H_2_O)_2_]·1/2CH_3_CH_2_CN (**2**)	2.1324(12), 2.1457(14)	2.0937(10), 2.0976(11)	76.80(5), 78.67(5)	H_2_O	2.0711(12), 2.0789(12)
*cis*-[Zn(pic)_2_(H_2_O)_2_]·1/2DMF^22^	2.134(4), 2.150(4)	2.094(3), 2.104(3)	76.58(15), 78.44(15)	H_2_O	2.078(4), 2.101(4)
*trans*-[Zn(pic)_2_(H_2_O)_2_]·2H_2_O^23^	2.1161(17)	2.0755(13)	79.57(6)	H_2_O	2.1544(14)

a An auxiliary ligand is bound in a monodentate manner.

**Figure 2:**
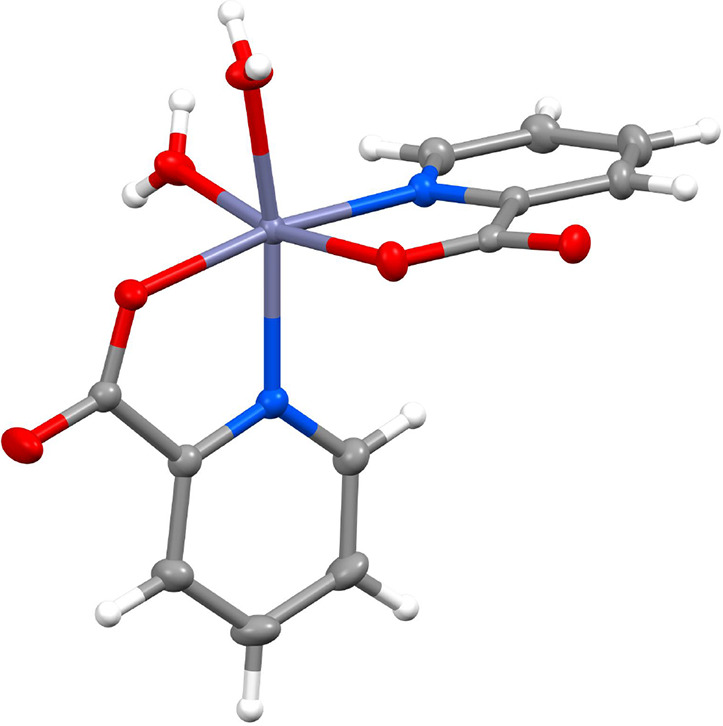
ORTEP drawing of *cis*-[Zn­(pic)_2_(H_2_O)_2_], a complex molecule in **2**, with thermal ellipsoids at the 50% probability level.

The picolinate-amino alcohol complexes share several common structural features. In addition to two *N*,*O*-bidentate chelating picolinates, all contain a single amino alcohol ligand that is likewise coordinated in the *N*,*O*-bidentate chelating manner. The new complex molecules of [Zn­(pic)_2_(2aeOH)] (**3**), [Zn­(pic)_2_(2maeOH)] (**4**), [Zn­(pic)_2_(2eaeOH)] (**5**), [Zn­(pic)_2_(2dmaeOH)] (**6**), [Zn­(pic)_2_(2a1pOH)]·H_2_O (**7**), [Zn­(pic)_2_(2a1pOH)] (**8**), [Zn­(pic)_2_(1a2bOH)]·CH_3_CN (**9**), and [Zn­(pic)_2_(1a2m2pOH)]·CH_3_OH (**10**) may be thus described as *tris-chelates*. Figure 3 shows the ORTEP drawing of [Zn­(pic)_2_(2maeOH)] (**4**), whereas drawings of other complex molecules form part of the SI. In all *tris-chelates*, the zinc­(II) centers are coordinated by N_3_O_3_ donor sets adopting a *mer* arrangement, whereas *fac* isomers with an N_3_O_3_ facial distribution were not observed. The presence of two different *N*,*O*-bidentate chelating ligands allows for three distinct *mer* isomers, which differ in the relative positions of the pyridine and carboxylate groups. Interestingly, all three are observed across the series of complexes **3**–**10**. Drawings of different isomers and their distribution among the *tris-chelates* are given in Scheme 4.

**Figure 3:**
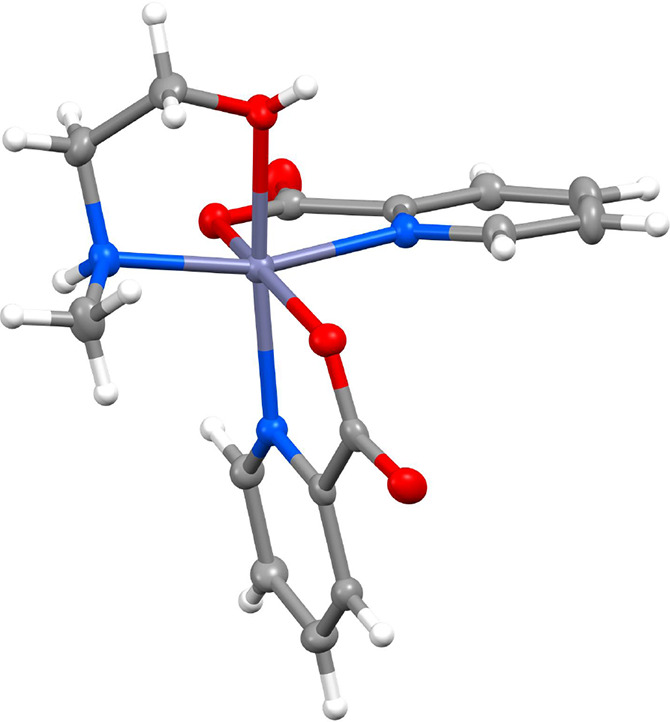
ORTEP drawing of [Zn­(pic)_2_(2maeOH)], a complex molecule in **4**, with thermal ellipsoids at the 50% probability level.

**Scheme 4:**
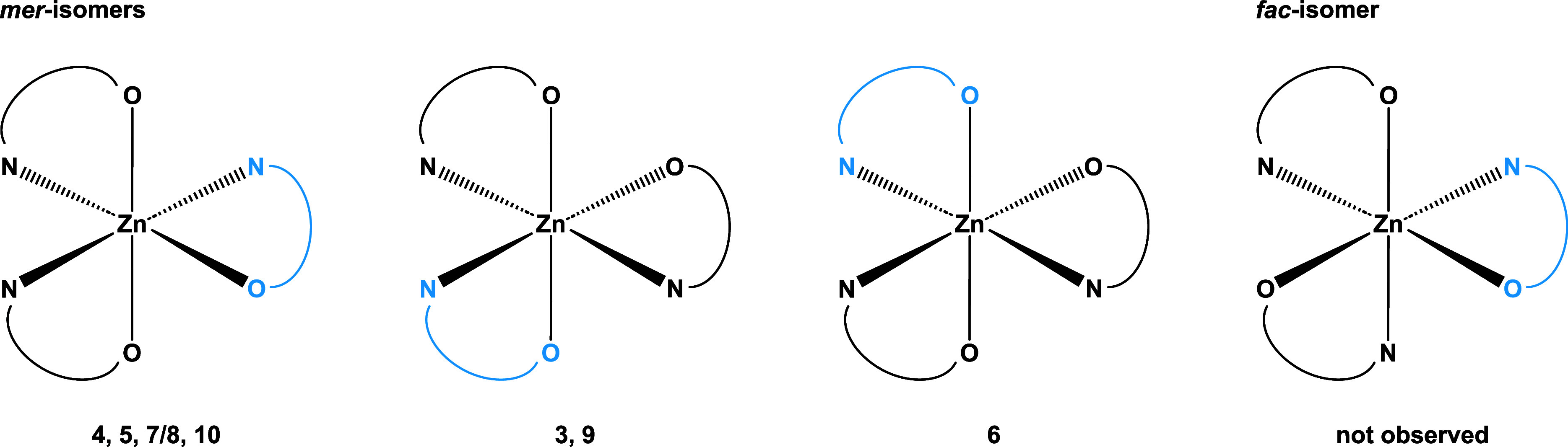
Drawings of *mer-* and *fac*-[Zn­(pic)_2_(amino alcohol)] Isomers^a^

a The *N*,*O*-bidentate chelating picolinate is depicted in black, while the amino alcohol ligand is shown in blue. Drawings are accompanied by labels indicating the complexes of each type.

Within the series, the zinc­(II)-amino alcohol bond lengths span a relatively wide range, i.e., 2.0708(12)–2.157(2) Å for the Zn–N bonds and 2.136(3)–2.2192(12) Å for the Zn–O bonds (Table 2). Owing to the crystallographically imposed disorder of the 2-aminoethanol ligand in [Zn­(pic)_2_(2aeOH)] (**3**), its geometric parameters are excluded from the discussion. The 1a2pOH complex, [Zn­(pic)_2_(2a1pOH)], presents a particularly instructive example, as it crystallizes in two distinct solid forms: a solvate with water molecules of crystallization, denoted as **7**, and a solvent-free modification, denoted as **8**. Comparison of their solid-state structures reveals a noticeable difference in the Zn–N­(amino alcohol) bond length, 2.078(3) Å in **7** vs 2.1257(12) Å in **8**. In contrast, the corresponding Zn–O­(amino alcohol) bonds in the two forms are essentially the same. Evaluation of the Zn–N­(amino alcohol) bond lengths throughout the entire series indicates that the ones bearing alkyl-substituted amine groups consistently lie at the longer end of the interval. Thus, [Zn­(pic)_2_(2dmaeOH)] (**6**), in which the amine nitrogen is substituted with two methyl groups, displays the longest Zn–N bond, 2.157(2) Å. A related bond in [Zn­(pic)_2_(2eaeOH)] (**5**), whose amine group possesses a single ethyl substituent, is slightly shorter, 2.1353(14) Å, whereas [Zn­(pic)_2_(2maeOH)] (**4**) with an HNMe group exhibits an even shorter one, 2.1040(13) Å. For comparison, the corresponding Zn–N bond in [Zn­(pic)_2_(1a2m2pOH)]·CH_3_OH (**10**) with the unsubstituted NH_2_ group amounts to 2.0708(12) Å. This progressive lengthening of the Zn–N bonds with increasing alkyl substitution reflects the expected reduction in donor strength of the amine nitrogen as steric effects become more pronounced. A similar trend is observed for literature examples. Thus, *cis*-[Zn­(sac)_2_(2dmaeOH)_2_] (sac^–^ = saccharinate)^35^ and [Zn­(acac)_2_(2dmaeOH)] (acac^–^ = acetylacetonate),^34^ both featuring the *N*,*O*-bidentate chelating 2dmaeOH ligands, exhibit Zn–N bonds that are longer than 2.2 Å. In contrast, the 2aeOH complex [Zn­(sac)_2_(2aeOH)_2_][Bibr ref27] displays a significantly shorter Zn–N bond of 2.0873(9) Å. The length is consistent with the absence of alkyl substitution at the amine group and is comparable to the Zn–N bond in [Zn­(pic)_2_(1a2m2pOH)]·CH_3_OH (**10**). A dinuclear zinc­(II) complex with (*S*)-2-amino-2-phenylethan-1-ol is also noteworthy as the amino alcohol ligand exhibits two coordination modes: bidentate chelating and monodentate through the amine nitrogen.^36^ The Zn–N bond length associated with the bidentate ligand is 2.068(13) Å, while that involving the monodentate ligand is as short as 2.047(13) Å. The series of novel *tris-chelates* displays a rather narrow interval of amino alcohol bite angles. Similarly, the picolinate bite angles fall within the interval that is less than 2° wide. In contrast, the zinc­(II)-to-picolinate bonds occupy wider intervals; the Zn–N bond ranges from 2.0996(11) to 2.179(2) Å and the Zn–O bond from 2.0359(18) to 2.1467(10) Å. A pair of the Zn–N bonds can be essentially equal as in the 2a1pOH compound **8** or markedly different as in the 1a2m2pOH compound **10**. The (subtle) differences in the overall geometry of the complexes are a joint result of the binding ability of a particular amino alcohol ligand and its involvement in hydrogen-bonding interactions.

**Table 2:** Relevant Geometric Parameters [Å,°] of *Tris-chelates*

	Zn–amino alcohol			Zn–picolinate^a^		
compound	Zn–N	Zn–O	amino alcohol bite angle	Zn–N	Zn–O	picolinate bite angle^a^
[Zn(pic)_2_(2aeOH)] (**3**)	2.179(15)	2.121(13)	80.4(2)	2.1669(15)	2.0654(17)	78.34(7)
[Zn(pic)_2_(2maeOH)] (**4**)	2.1040(13)	2.1734(12)	79.79(5)	2.1084(13), 2.1735(13)	2.0970(11), 2.1116(10)	76.99(4), 78.66(5)
[Zn(pic)_2_(2eaeOH)] (**5**)	2.1353(14)	2.2192(12)	79.82(5)	2.1190(13), 2.1516(13)	2.0851(11), 2.0962(11)	77.40(5), 78.69(5)
[Zn(pic)_2_(2dmaeOH)] (**6**)	2.157(2)	2.136(3)	79.39(12)	2.176(2), 2.179(2)	2.0359(18), 2.082(3)	77.97(11), 78.16(8)
[Zn(pic)_2_(2a1pOH)]·H_2_O (**7**)	2.078(3)	2.202(2)	78.91(10)	2.109(3), 2.161(2)	2.100(2), 2.110(2)	77.00(9), 78.10(9)
[Zn(pic)_2_(2a1pOH)] (**8**)	2.1257(12)	2.2001(9)	79.03(4)	2.1469(12), 2.1508(10)	2.0905(9), 2.0912(9)	77.80(4), 78.44(4)
[Zn(pic)_2_(1a2bOH)]·CH_3_CN (**9**)	2.0849(18)	2.2092(16)	78.06(7)	2.1319(18), 2.1413(17)	2.1149(15), 2.1155(15)	77.13(6), 77.50(6)
[Zn(pic)_2_(1a2m2pOH)]·CH_3_OH (**10**)	2.0708(12)	2.1946(10)	79.43(4)	2.0996(11), 2.1680(11)	2.0946(9), 2.1467(10)	77.25(4), 77.70(4)

a Parameters are listed in the order from the smallest to largest.

The solid-state structure of the ionic compound (1a2m2pOH_2_)­[Zn­(pic)_3_]·H_2_O (**11**) consists of protonated amino alcohol, anionic [Zn­(pic)_3_]^−^ complex, and water molecules of crystallization. In the [Zn­(pic)_3_]^−^ complex, the metal ion is coordinated with three *N*,*O*-bidentate chelating picolinates (Figure 4). Their mutual arrangement results in a *mer* distribution of the N_3_O_3_ donor set. As expected, the Zn–Obonds [2.0932(12)–2.1169(12) Å] are, on average, shorter than the Zn–N bonds [2.0972(14)–2.1869(14) Å]. Interestingly, a much narrower range of bond lengths was observed in the methanol solvate (1a2m2pOH_2_)­[Zn­(pic)_3_]·CH_3_OH (**11a**) with Zn–O bonds of 2.0771(11)–2.0954(11) Å and the Zn–N bonds of 2.1410(13)–2.1594(13) Å. The previously reported [Zn­(pic)_3_]^−^ compound^43^ pipeamH­[Zn­(pic)_3_]·0.5H_2_O (pipeamH^+^ = protonated piperidinoacetamidine) also displayed narrow intervals of Zn–O and Zn–N bond lengths.

**Figure 4:**
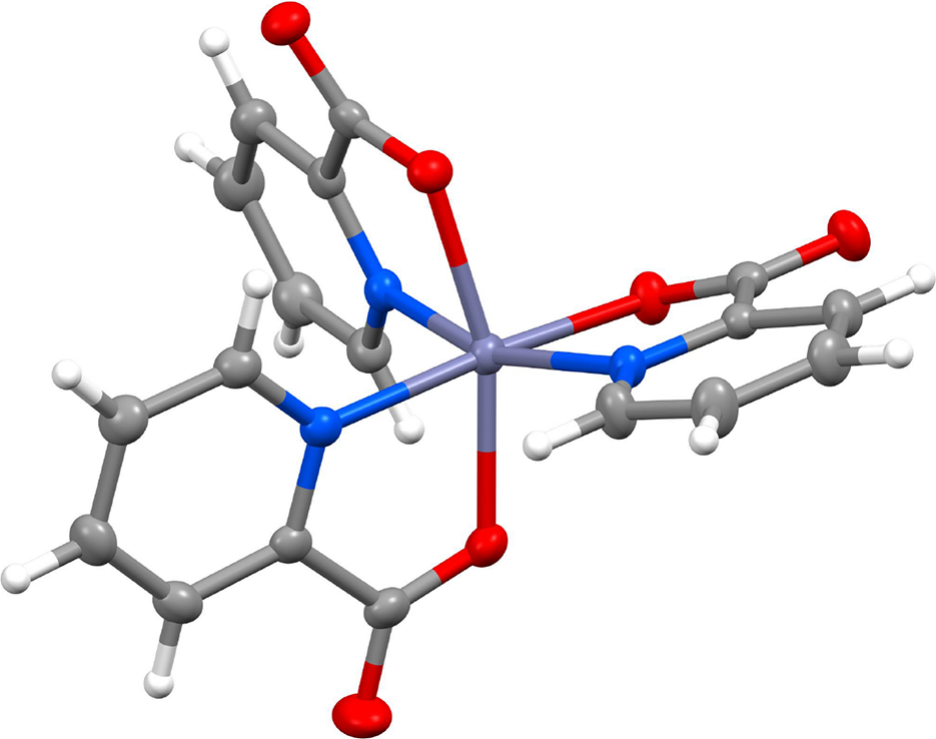
ORTEP drawing of [Zn­(pic)_3_]^−^, one of the two complex anions in the asymmetric unit of (1a2m2pOH_2_)­[Zn­(pic)_3_]·H_2_O (**11**), with thermal ellipsoids at the 50% probability level.

The solid-state structures of compounds **1**–**11a** are stabilized by hydrogen bonds. The SI provides an exhaustive list of hydrogen bonds (Table S1) and drawings of connectivity patterns. In *trans*-[Zn­(pic)_2_(CH_3_OH)_2_] (**1**) and *cis*-[Zn­(pic)_2_(H_2_O)_2_]·1/2CH_3_CH_2_CN (**2**), the picolinate ligands act as hydrogen-bond acceptors, whereas the coordinated methanol and water molecules function as donors. In *trans*-[Zn­(pic)_2_(CH_3_OH)_2_] (**1**), the CH_3_OH···COO^–^ interactions link complex molecules into a supramolecular chain. In this chain, each molecule forms four hydrogen bonds with two adjacent molecules. In *cis*-[Zn­(pic)_2_(H_2_O)_2_]·1/2CH_3_CH_2_CN (**2**), the H_2_O···COO^–^ interactions link complex molecules into an infinite two-dimensional layer. The stacking of layers one upon another produces pockets that are filled with propionitrile solvent molecules.

The structures of picolinate–amino alcohol complexes display a variety of connectivity patterns. All of the amino alcohol ligands possess a hydroxyl group and, with the exception of 2-dimethylaminoethanol, also NH_2_ or NH groups, which participate in hydrogen-bonding interactions. The structures of [Zn­(pic)_2_(2aeOH)] (**3**) and [Zn­(pic)_2_(2a1pOH)] (**8**) that both contain amino alcohols with NH_2_ group display different supramolecular patterns. In [Zn­(pic)_2_(2aeOH)] (**3**), the OH···COO^–^ and NH_2_···COO^–^ hydrogen bonds among complex molecules result in infinite layers, whereas the infinite chains in [Zn­(pic)_2_(2a1pOH)] (**8**) are sustained solely by the OH···COO^–^ interactions (Figure 5). In **8**, the amino group of 2-amino-1-propanol makes a contact with the carboxylate oxygen, but the N···O separation exceeds the sum of the van der Waals radii.^44^ A longer contact could be attributed to a steric hindrance caused by the methyl substituent on the −C–C– backbone of the amino alcohol. However, the structure of [Zn­(pic)_2_(2a1pOH)]·H_2_O (**7**) overrules this explanation. In **7**, the water molecules of crystallization, as expected, participate in the hydrogen-bonding scheme, and two-dimensional layers are formed (Figure 6). These layers are sustained among other types of interactions also by the NH_2_···H_2_O hydrogen bonds. [Zn­(pic)_2_(1a2bOH)]·CH_3_CN (**9**), a complex with another amino alcohol that bears an NH_2_ moiety, possesses acetonitrile solvent molecules of crystallization. Since acetonitrile does not interfere with the supramolecular connectivity, a two-dimensional layer structure is formed by the OH···COO^–^ and NH_2_···COO^–^ hydrogen bonding interactions. Evidently, the ethyl substituent of 1-amino-2-butanol does not impose steric hindrance on either functional group. This is not the case for the 1-amino-2-methyl-2-propanol complex [Zn­(pic)_2_(1a2m2pOH)]·CH_3_OH (**10**), in which the NH_2_ group participates only in weak interactions. In the structure of **10**, methanol has a pronounced influence over the hydrogen-bonding pattern. With methanol being hydrogen-bonded to one of the two carboxylate groups, only the remaining carboxylate is available to interact with the OH group of amino alcohol. Consequently, two complex molecules are linked by two OH···COO^–^ hydrogen bonds to form a “dimer”. The supramolecular structures of complexes with mono-*N*-alkylsubstituted amino alcohols are also different. In [Zn­(pic)_2_(2maeOH)] (**4**), a complex with 2-methylaminoethanol, the OH···COO^–^ bonds link complex molecules into dimers, whereas the NH group engages only in a weak contact with a carboxylate oxygen. Interestingly, in [Zn­(pic)_2_(2eaeOH)] (**5**) whose amino alcohol, 2-ethylaminoethanol, bears a bulkier alkyl substituent, the NH moiety nevertheless participates in hydrogen bonding. As a result, both the NH···COO^–^ and the OH···COO^–^ interactions connect complex molecules into chains. In [Zn­(pic)_2_(2dmaeOH)] (**6**) which contains 2-dimethylaminoethanol and therefore lacks an NH group, the supramolecular assembly is governed solely by OH···COO^–^ hydrogen bonds and results in infinite chains.

**Figure 5:**
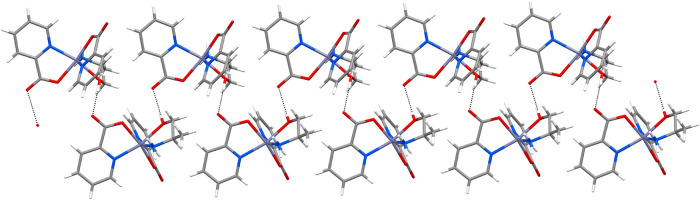
A supramolecular chain in the structure of [Zn­(pic)_2_(2a1pOH)] (**8**).

**Figure 6:**
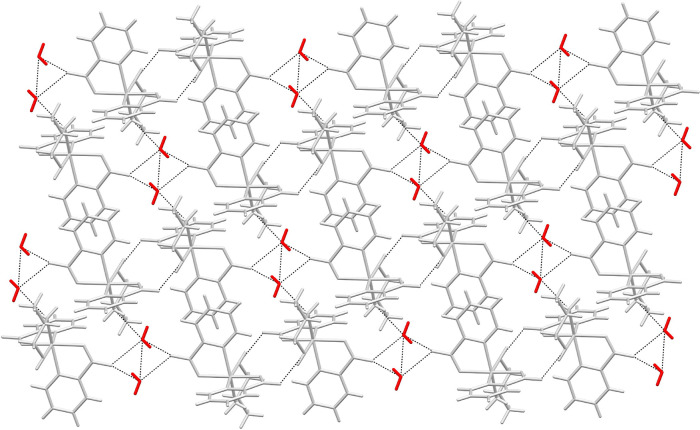
A perpendicular view of the supramolecular layer in the structure of [Zn­(pic)_2_(2a1pOH)]·H_2_O (**7**). Color code: gray, complex molecules; red, water molecules of crystallization.

The structure of the ionic compound (1a2m2pOH_2_)­[Zn­(pic)_3_]·H_2_O (**11**) features an intricate hydrogen-bonding network that gives rise to a three-dimensional framework. Similarly, a hydrogen-bonding pattern in the methanol solvate (1a2m2pOH_2_)­[Zn­(pic)_3_]·CH_3_OH (**11a**) produces a three-dimensional supramolecular structure. In alternative description, the structure of **11a** may be described as layers, consisting of [Zn­(pic)_3_]^−^ anions with attached protonated amino alcohol molecules, held together by methanol solvent molecules.

### DFT Calculations

Calculations were performed at the DFT-B3PW91-D3BJ/def2-TZVP-SMD­(methanol) level of theory, starting from the solid-state XRD molecular structure of the exemplary complex **4** (labeled as **4** ***mer*** in the paragraph that follows). In addition, a second, almost isoenergetic, *mer* isomer, **4** ***mer*** ***_b***, was optimized; this species differs in the arrangement of the substituents around the chiral amine nitrogen. The corresponding *fac* isomers **4** ***fac*** and **4** ***fac*** ***_b*** were also examined, together with a trigonal–bipyramidal isomer **4** ***tbp*** arising from the decoordination of the O–H oxygen and the formation of hydrogen bond to one of the picolinate oxygen atoms. The calculations indicate that the experimentally observed isomer is thermodynamically the most stable, with the *fac* isomers lying only 1.9 kcal/mol higher in energy, which suggests that these species may not be observable. Decoordination of the O–H in **4** ***mer*** requires 1.7 kcal/mol. A possible pathway for isomerization of **4**, suggested by the **4** ***tbp*** isomer, would also require rearrangement of picolinate ligands. These results are summarized in Figure 7.

**Figure 7:**
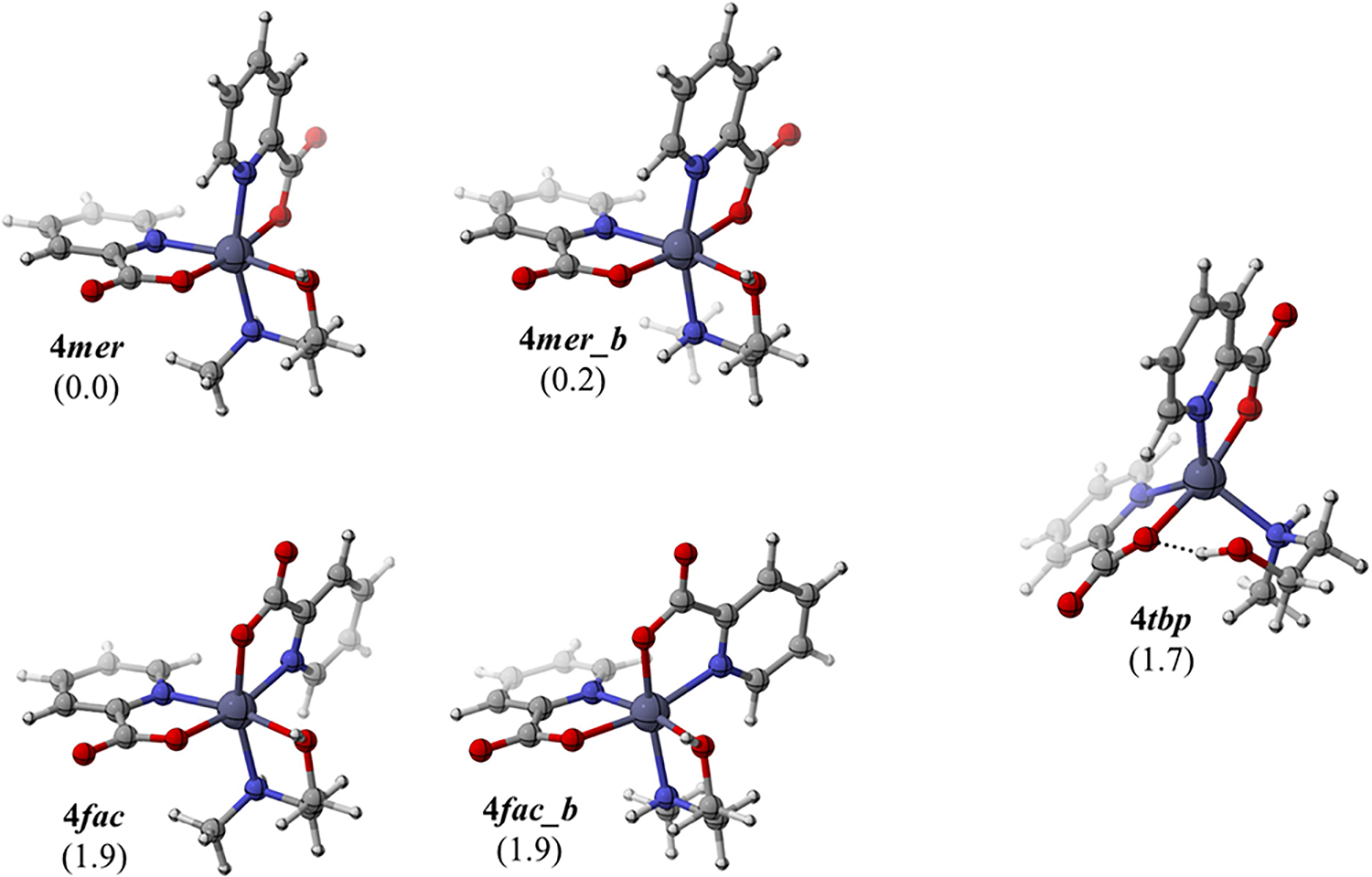
Optimized geometries for stereoisomers of **4**. Relative free energies in kcal/mol are shown in parentheses.

### Antibacterial Activity

Representative zinc­(II) complexes featuring both types of ligands were selected for biological activity studies. The compounds chosen for testing were required to incorporate amino alcohols with different substitution patterns. Accordingly, the antibacterial activity of selected zinc­(II) complexes with picolinate and amino alcohols, [Zn­(pic)_2_(2aeOH)] (**3**), [Zn­(pic)_2_(2maeOH)] (**4**), [Zn­(pic)_2_(2dmaeOH)] (**6**), and [Zn­(pic)_2_(2a1pOH)]·H_2_O (**7**), was evaluated against six bacterial strains, including the Gram-negative species *Escherichia coli*, *Pseudomonas aeruginosa*, and *Proteus mirabilis*, as well as the Gram-positive species *Staphylococcus aureus*, *Bacillus subtilis*, and *Staphylococcus epidermidis* (Table 3). The stability of the complexes in DMSO solution was monitored by ^1^H NMR spectroscopy. No changes in the spectra were observed over a 48 h period, indicating that the complexes remain stable under these conditions. While picolinic acid showed no antibacterial activity at a concentration of 128 μg/mL, all tested zinc­(II) complexes exhibited moderate activity against *S. epidermidis*, with minimum inhibitory concentration (MIC) values ranging from 32 to 64 μg/mL. No inhibitory effects were observed against *E. coli*, *S. aureus*, *P. aeruginosa*, *P. mirabilis*, or *B. subtilis* under the conditions tested.

**Table 3:** Results of Antibacterial Testing

	MIC [μg/mL]					
compound	*E. coli*	*S. aureus*	*P. aeruginosa*	*P. mirabilis*	*B. subtilis*	*S. epidermidis*
picolinic acid	>128	>128	>128	>128	>128	>128
[Zn(pic)_2_(2aeOH)] (**3**)	>128	>128	>128	>128	>128	32
[Zn(pic)_2_(2maeOH)] (**4**)	>128	>128	>128	>128	>128	64
[Zn(pic)_2_(2dmaeOH)] (**6**)	>128	>128	>128	>128	>128	64
[Zn(pic)_2_(2a1pOH)]·H_2_O (**7**)	>128	>128	>128	>128	>128	32
tetracycline	1	0.5	>4	>4	0.5	>4

The lack of antibacterial activity observed for picolinic acid is consistent with previous reports. A series of Cu­(II), Co­(II), Pt­(II), and Zn­(II) picolinate complexes have been reported to exhibit generally weak antibacterial activity against a range of Gram-positive and Gram-negative bacterial strains.^45,46^ In contrast, ruthenium­(II) complexes incorporating picolinate and phosphine ligands displayed enhanced antibacterial potency against *Mycobacterium tuberculosis*, with MIC values of 0.49–0.91 μg/mL, significantly outperforming free picolinic acid (MIC > 50 μg/mL).^47^ [Zn­(sal)_2_(met)_2_], a zinc­(II) complex containing salicylate and metronidazole ligands, exhibited a similar selectivity profile to the present complexes but with substantially higher MIC values. This compound showed its greatest activity against *S. epidermidis* (MIC ≈ 600 μg/mL), while weaker activity was observed against *S. aureus*, *E. coli*, and *P. aeruginosa* with MIC values of approximately 950, 1350, and 2050 μg/mL, respectively.^48^ Same selectivity but markedly greater activity against *S. epidermidis* (MIC = 8 μg/mL) was observed for [Zn_7_O_2_(CH_3_COO)_10_(met)_2_]·2CH_3_CN, a heptanuclear zinc­(II) complex with metronidazole that was prepared in our laboratory.^49^ The selective activity of the latter and the novel zinc­(II) complexes with picolinate and amino alcohols **3**, **4**, **6** and **7** may be associated with the distinctive surface properties of *S. epidermidis*. This species possesses a highly anionic cell surface that can interact with polar metal complexes. The higher antibacterial potency of complexes **3** and **7**, which contain 2-aminoethanol or 2-amino-1-propanol, amino alcohols with primary amino groups, compared to **4** and **6**, which contain 2-methylaminoethanol or 2-dimethylaminoethanol, amino alcohols with methyl-substituted nitrogen, suggests that the hydrogen-bonding capacity and greater polarity of primary amines enhance interactions and contribute to improved antimicrobial activity. Given the increasing prevalence of multidrug-resistant *S. epidermidis* strains,^50^ the development of new antimicrobial agents targeting this species is of growing importance. *S. epidermidis* normally resides on human skin and mucous membranes, acting mostly as a harmless commensal organism. However, it can become an opportunistic pathogen, particularly in hospital settings, where it is a major cause of infections associated with implanted medical devices.^51,52^ Unlike the more virulent *S. aureus*, infections caused by *S. epidermidis* generally result from accidental colonization and biofilm formation rather than toxin-mediated pathogenicity. This biofilm formation protects the bacteria from both antibiotics and the host immune response, making infections persistent and difficult to eradicate.^53^

### IR Spectroscopy

The infrared spectra of zinc­(II) complexes with picolinate and amino alcohols form part of the Supporting Information. Owing to the complexity of these spectra, the discussion is limited to the most prominent absorption bands of key structural elements. The coordinated picolinate ion imparts a distinctive spectral fingerprint that is consistently observed across the entire series of complexes, as summarized in Table 4. For comparison, the spectrum of a related compound *trans*-[Co­(pic)_2_(H_2_O)_2_]·2H_2_O exhibits three very intense bands in the regions associated with the ν_as_(COO^–^), ν­(C=N) and ν­(C=C) vibrations. Their positions, at 1630, 1596, and 1569 cm^–1^, match with the ones observed in the spectra of our compounds. The ν_s_(COO^–^) absorption in *trans*-[Co­(pic)_2_(H_2_O)_2_]·2H_2_O may be seen at 1374 cm^–1^, while the deformation vibrations of pyridine ring occur at 765 and 703 cm^–1^. The authors tentatively assigned the low-frequency bands at 445 and 422 cm^–1^ to the ν­(Co–N) and ν­(Co–O) absorptions, respectively.^54^ Monodentate carboxylate coordination is typically reflected in a large difference between the position of the ν_as_(COO^–^) and ν_s_(COO^–^) absorption bands, commonly referred to as the splitting value Δ.^55^ In the spectra of compounds **1**–**11a**, the splitting values occupy a 245–291 cm^–1^ interval, which agrees with a monodentate binding mode.

**Table 4:** Characteristic Picolinate Bands [cm^–1^] in the Spectra of Novel Zinc­(II) Compounds

compound	ν_as_(COO^–^), ν(C=N), and ν(C=C)	ν_s_(COO^–^)	pyridine deformation vibrations	
*trans*-[Zn(pic)_2_(CH_3_OH)_2_] (**1**)	1624, 1589, 1565	1376	758, 695, 645	439, 417
*cis*-[Zn(pic)_2_(H_2_O)_2_]·1/2CH_3_CH_2_CN (**2**)	1619, 1588, 1566	1370	760, 698, 637	431, 410
[Zn(pic)_2_(2aeOH)] (**3**)	1626, 1591, 1562	1387, 1372, 1335	751, 698, 637	429, 411
[Zn(pic)_2_(2maeOH)] (**4**)	1622, 1591, 1566	1382, 1352	760, 699, 639	428, 416
[Zn(pic)_2_(2eaeOH)] (**5**)	1622, 1589, 1566	1380, 1350	761, 700, 639	431
[Zn(pic)_2_(2dmaeOH)] (**6**)	1634, 1620, 1589, 1562	1388, 1364	774, 753, 697, 637	432, 422
[Zn(pic)_2_(2a1pOH)]·H_2_O (**7**)	1622, 1590, 1565	1373	763, 700, 639	430, 415
[Zn(pic)_2_(1a2bOH)]·CH_3_CN (**9**)	1631, 1595, 1568	1357	755, 698, 641	431
[Zn(pic)_2_(1a2m2pOH)]·CH_3_OH (**10**)	1625, 1591, 1566	1372	761, 699, 639	430, 416
(1a2m2pOH_2_)[Zn(pic)_3_]·CH_3_OH (**11a**)	1618, 1589, 1565	1373	757, 700, 639	430

Both functional groups and the CH bonds of amino alcohol ligands are reflected in the infrared spectra of their complexes. The characteristic absorption bands associated with coordinated amino alcohols are summarized in Table 5. In general, the spectra of compounds **3**–**10** exhibit a series of weak to medium-intensity bands corresponding to the ν­(O–H) and ν­(N–H) vibrations. However, these bands cannot be assigned unambiguously due to their overlapping. For instance, [Zn­(pic)_2_(2aeOH)] (**3**), a 2-aminoethanol complex, displays a weak but sharp absorption band at 3348 cm^–1^ in this region. A complex with 2-methylaminoethanol, [Zn­(pic)_2_(2maeOH)] (**4**), as another example, is characterized by a sharp band at a lower frequency, 3244 cm^–1^. Similarly, [Zn­(pic)_2_(2eaeOH)] (**5**), a complex with 2-ethylaminoethanol, an analogue of 2-methylaminoethanol, displays a significantly weaker band in the same spectral region. On the other hand, [Zn­(pic)_2_(2dmaeOH)] (**6**), a complex with 2-dimethylaminoethanol that possesses a tertiary amino group, shows no absorption in this region. Although the observation is consistent with the absence of the N–H stretching vibrations, the absence of the O–H stretching cannot be accounted for. The presence of methyl, methylene, or methine groups is shown by several even weaker bands in the 3000–2740 cm^–1^ spectral region that is characteristic for the ν­(C–H) vibrations. Depending on their immediate chemical environment, the hydroxyl groups can be classified as belonging to primary, secondary, or tertiary alcohols. According to theory, the different types of alcohols can be distinguished by their ν­(C–O) regions: primary alcohols absorb in the 1075–1000 cm^–1^ range, secondary alcohols in the 1150–1075 cm^–1^ range, and tertiary alcohols in the 1210–1100 cm^–1^ range.^56^

**Table 5:** Characteristic Amino Alcohol Bands [cm^–1^] in the Spectra of Novel Zinc­(II) Complexes

amino alcohol complexes with primary OH groups			
compound	ν(O–H) and ν(N–H) region	ν(C–H)	ν(C–O)
[Zn(pic)_2_(2aeOH)] (**3**)	3348, 3220	2954–2803	1052, 1020, 1001
[Zn(pic)_2_(2maeOH)] (**4**)	3244, 3195	2999–2736	1066, 1042, 1001
[Zn(pic)_2_(2eaeOH)] (**5**)	3225, 3184	2976–2738	1067, 1043, 1016
[Zn(pic)_2_(2dmaeOH)] (**6**)		2979–2721	1076, 1047, 1030, 1014
[Zn(pic)_2_(2a1pOH)]·H_2_O (**7**)	3480, 3255, 3173^a^	2964–2884	1042, 1020

amino alcohol complex with secondary OH groups			
compound	ν(O–H) and ν(N–H) region	ν(C–H)	ν(C–O)
[Zn(pic)_2_(1a2bOH)]·CH_3_CN (**9**)	3258, 3166	2963–2880	1147, 1114, 1090, 1062, 1048, 1020

amino alcohol complex with tertiary OH groups			
compound	ν(O–H) and ν(N–H) region	ν(C–H)	ν(C–O)
[Zn(pic)_2_(1a2m2pOH)]·CH_3_OH (**10**)	3373, 3282, 3176	2963–2817	1149, 1095, 1045, 1019

a The water ν­(O–H) vibration also appears in this region.

As shown in Table 5, a number of absorption bands are observed in the region of the ν­(C–O) absorptions. For the amino alcohol complexes with primary OH groups, the observed regions are in good agreement with the expected values. The highest frequency band appears in the spectrum of **6** at 1076 cm^–1^ and the lowest one at 1001 cm^–1^ in the spectra of **3** and **4**. The spectrum of the secondary alcohol complex **9** features bands within the 1147–1020 cm^–1^ range, which is slightly broader than typically reported in the literature. The spectrum of **10**, a complex with 1-amino-2-methyl-2-propanol, a formally tertiary alcohol, combines features of the coordinated amino alcohol and methanol solvent molecules. The absorptions of both occupy a 1149–1019 cm^–1^ spectral interval. The presence of protonated tertiary amino alcohol in **11a** may be inferred from a series of weak bands in the 1240–1148 cm^–1^ spectral range, whereas stronger bands in the 1090–1019 cm^–1^ region speak of the residual methanol. The participation of the 1a2m2pOH_2_^+^ ion in the hydrogen bonding manifests itself in broad bands centered at *ca*. 3330 and 2900 cm^–1^. The infrared spectrum of *trans*-[Zn­(pic)_2_(CH_3_OH)_2_] (**1**) confirms the presence of coordinated methanol molecules, shown by a series of weak bands at 2982–2806 cm^–1^ and a strong band at 1023 cm^–1^ with a shoulder at 1043 cm^–1^. The former bands find their origin in the ν­(C–H) vibrations and the latter in the ν­(C–O) vibration. Over time, the ν­(C–O) band intensity decreases markedly and is accompanied by the emergence and growth of a broad absorption centered at *ca*. 3400 cm^–1^. The observed spectral changes indicate a loss of coordinated methanol and the concomitant uptake of atmospheric moisture. The presence of coordinated water molecules in *cis*-[Zn­(pic)_2_(H_2_O)_2_]·1/2CH_3_CH_2_CN (**2**) is shown by two broad absorptions: a high-frequency band centered at 3163 cm^–1^, attributable to ν­(O–H) vibrations, and another band near 650 cm^–1^. A weak band at 2248 cm^–1^ undoubtedly confirms the incorporation of propionitrile solvent molecules. A comparable weak absorption at 2251 cm^–1^ was observed for another nitrile solvate, [Zn­(pic)_2_(1a2bOH)]·CH_3_CN (**9**).

## Conclusions

A series of seven β-amino alcohols were reacted with the methanolic solution of {Zn­(pic)_2_} to afford a new class of zinc­(II) complexes of general formula [Zn­(pic)_2_(amino alcohol)]. In addition to targeted products, two simple picolinate complexes, *trans*-[Zn­(pic)_2_(CH_3_OH)_2_] and *cis*-[Zn­(pic)_2_(H_2_O)_2_]·1/2CH_3_CH_2_CN, and two ionic [Zn­(pic)_3_]^−^ compounds were also obtained. With the sole exception of one compound, the remaining 12 were obtained in the form of single crystals. In all [Zn­(pic)_2_(amino alcohol)] complexes, both picolinate and amino alcohol ligands coordinated in an *N*,*O*-bidentate chelating mode, generating an octahedral coordination environment around the zinc­(II) center. A *meridional* distribution of N_3_O_3_ donor set was observed consistently across the entire series. A preference for *mer* distribution was further confirmed by DFT calculations on the 2-methylaminoethanol complex that showed the experimentally observed isomer as the thermodynamically most stable one, with the *fac* isomer lying only 1.9 kcal/mol higher in energy. Systematic variation of the amino alcohol ligands, differing in the position of methyl or ethyl substituents, enabled evaluation of substituent effects on the supramolecular organization of the complex molecules in the solid state. Representative complexes were assessed for antibacterial activity against a panel of Gram-positive and Gram-negative bacterial strains and exhibited pronounced selectivity toward *Staphylococcus epidermidis*, with no significant activity observed against other tested strains. Notably, complexes incorporating amino alcohols bearing unsubstituted NH_2_ groups showed enhanced antibacterial potency compared with those containing *N*-alkyl-substituted amino alcohols, underscoring the importance of amine substitution in modulating biological activity. These findings provide a foundation for the rational design of zinc­(II) complexes with tailored supramolecular architectures and targeted antibacterial properties.

## Experimental Section

### General

All reagents were obtained from commercial sources and used as received, except for acetonitrile, which was dried over molecular sieves prior to use.^57^ Infrared spectra were recorded on a Bruker Alpha II FTIR spectrophotometer equipped with an attenuated total reflection (ATR) module in the 4000–400 cm^–1^ range. Spectra are presented without further corrections. Band intensities are denoted as follows: w = weak, m = medium, s = strong, vs = very strong, and vvs = very very strong. ^1^H NMR spectra were acquired on a Bruker Avance NEO 600 MHz instrument. Deuterated dimethyl sulfoxide ((CD_3_)_2_SO) with 0.03% tetramethylsilane (TMS) standard was used as solvent. Chemical shifts were referenced to the residual (CD_3_)_2_SO signal at 2.50 ppm or TMS standard at 0.00 ppm.^58^ Chemical shifts (δ) are reported in ppm, and coupling constants (*J*) are in Hz. Multiplicities are denoted as follows: s = singlet, d = doublet, t = triplet, q = quartet, dd = doublet of doublets, m = multiplet, and br = broad signal. Spectra were processed using the MestReNova software (version 14.2.2).^59^ The elemental analyses of carbon, nitrogen, and hydrogen were carried out using a PerkinElmer 2400 II analyzer. High-resolution mass spectra (HRMS) of compounds **4** and **5** were obtained on an Agilent 6224 Mass TOF LC Mass Spectrometer. The other *tris-chelates* exhibited solubility in acetonitrile that was too low to permit HRMS analysis.

### X-ray Structure Analysis

Single-crystal X-ray diffraction data for **1**–**11** were collected on an Agilent SuperNova diffractometer equipped with a molybdenum microfocus sealed X-ray source (Mo Kα, λ = 0.71073 Å) at 150 K. Data for**11a** were collected on an XtaLAB Synergy single-crystal X-ray diffractometer, also using Mo Kαradiation. Crystals were mounted on a glass fiber tip using silicone grease and placed on the goniometer head. Data reduction and processing were carried out with CrysAlis PRO.^60^ The structures were solved using the Olex^2^ software^61^ with ShelXT^62^ and refined with least-squares methods in ShelXL.^63^ Anisotropic displacement parameters were applied to all nonhydrogen atoms. Where possible, the hydrogen atoms on heteroatoms were located from difference Fourier maps and refined with isotropic displacement parameters. The remaining hydrogen atoms were added in calculated positions and refined using a riding model. The disorder of the propionitrile molecules in **2** could not be modeled. The contribution of the disordered solvent to the scattering factors was therefore accounted for using the solvent mask function. In complex **3**, the 2aeOH ligand showed a symmetry-imposed disorder about a twofold rotation axis. The disorder was treated using a PART −1 instruction. Although modeled, the disorder affects the bonding pattern and limits comparison with related complexes. In **9**, the disorder of the ethyl group in the 1a2bOH ligand was modeled using the PART instruction. The first component accounts for 84%, and the second accounts for 16%. The programs Platon^64^ and Mercury^65^ were used for crystal structure analysis and preparation of figures. Crystallographic data for **1**–**11a** are summarized in Tables 6–8. All crystal structures were deposited to the CCDC and were assigned deposition numbers 2522126 (**1**), 2522127 (**2**), 2522128 (**3**), 2522129 (**4**), 2522130 (**5**), 2522131 (**6**), 2522132 (**7**), 2522133 (**8**), 2522134 (**9**), 2522135 (**10**), 2522136 (**11**), and 2531934 (**11a**)

**Table 6:** Crystallographic Data for **1**–**5**

	*trans*-[Zn(pic)_2_(CH_3_OH)_2_] (**1**)	*cis*-[Zn(pic)_2_(H_2_O)_2_]·1/2CH_3_CH_2_CN (**2**)^a^	[Zn(pic)_2_(2aeOH)] (**3**)	[Zn(pic)_2_(2maeOH)] (**4**)	[Zn(pic)_2_(2eaeOH)] (**5**)
empirical formula	C_14_H_16_N_2_O_6_Zn	C_12_H_12_N_2_O_6_Zn	C_14_H_15_N_3_O_5_Zn	C_15_H_17_N_3_O_5_Zn	C_16_H_19_N_3_O_5_Zn
formula weight	373.66	345.61	370.66	384.68	398.71
crystal system	monoclinic	monoclinic	orthorhombic	triclinic	triclinic
space group	*P*2_1_/*n*	*I*2/*a*	*Fdd*2	*P*1̅	*P*1̅
*T* [K]	150.00(10)	150.00(10)	150.00(10)	150.00(10)	150.00(10)
λ [Å]	0.71073	0.71073	0.71073	0.71073	0.71073
*a* [Å]	8.9036(4)	16.5486(7)	30.7178(13)	9.0178(3)	8.5732(6)
*b* [Å]	6.2414(2)	8.6125(4)	12.6168(5)	9.2349(3)	9.9549(7)
*c* [Å]	14.2155(7)	21.0707(10)	7.6147(3)	11.6958(4)	11.1840(5)
α [°]	90	90	90	91.306(3)	84.323(4)
β [°]	107.618(5)	94.345(4)	90	107.648(3)	86.723(4)
γ [°]	90	90	90	117.482(4)	65.956(7)
*V* [Å^3^]	752.92(6)	2994.5(2)	2951.2(2)	808.04(5)	867.26(10)
*Z*	2	8	8	2	2
*D*_**calc**_[g/cm^3^]	1.648	1.533	1.668	1.581	1.527
μ [mm^–1^]	1.664	1.667	1.695	1.551	1.448
collected reflections	18,015	19,254	21,203	24,102	14,434
unique reflections	2177	4231	2158	4544	4781
observed reflections	1875	3589	2102	4022	4181
*R*_int_	0.0485	0.0366	0.0386	0.0427	0.0662
*R*_1_ (*I* > 2σ(*I*))	0.0267	0.0273	0.0183	0.0289	0.0344
*wR*_2_ (all data)	0.0710	0.0733	0.0460	0.0654	0.0807

a The solvent mask function was applied; therefore, the listed data pertain to *cis*-[Zn­(pic)_2_(H_2_O)_2_].

**7:** Crystallographic Data for **6**–**9**

	[Zn(pic)_2_(2dmaeOH)] (**6**)	[Zn(pic)_2_(2a1pOH)]·H_2_O (**7**)	[Zn(pic)_2_(2a1pOH)] (**8**)	[Zn(pic)_2_(1a2bOH)]·CH_3_CN (**9**)
empirical formula	C_16_H_19_N_3_O_5_Zn	C_15_H_19_N_3_O_6_Zn	C_15_H_17_N_3_O_5_Zn	C_18_H_22_N_4_O_5_Zn
formula weight	398.71	402.70	384.68	439.76
crystal system	orthorhombic	triclinic	monoclinic	triclinic
space group	*Pna*2_1_	*P*1̅	*C*2/*c*	*P*1̅
*T* [K]	150.00(10)	150.00(10)	150.00(10)	150.00(10)
λ [Å]	0.71073	0.71073	0.71073	0.71073
*a* [Å]	14.8354(4)	9.1123(4)	22.0116(5)	8.6376(3)
*b* [Å]	15.8563(5)	9.4387(4)	9.0428(2)	10.9509(4)
*c* [Å]	7.4120(2)	11.3428(5)	15.8297(4)	11.8469(4)
α [°]	90	93.156(4)	90	91.620(3)
β [°]	90	107.245(4)	97.793(2)	100.680(3)
γ [°]	90	113.760(4)	90	112.670(4)
*V* [Å^3^]	1743.56(9)	835.58(7)	3121.75(13)	1009.94(7)
*Z*	4	2	8	2
*D*_calc_ [g/cm^3^]	1.519	1.601	1.637	1.446
μ [mm^–1^]	1.440	1.507	1.605	1.252
collected reflections	42,055	16,671	75,748	25,151
unique reflections	5074	4657	4626	5682
observed reflections	4619	3798	4148	4506
*R*_int_	0.0511	0.0397	0.0505	0.0378
*R*_1_ (*I* > 2σ(*I*))	0.0314	0.0479	0.0244	0.0382
*wR*_2_ (all data)	0.0726	0.1359	0.0607	0.1073

**Table 8:** Crystallographic Data for **10** and **11**

	[Zn(pic)_2_(1a2m2pOH)]·CH_3_OH (**10**)	(1a2m2pOH_2_)[Zn(pic)_3_]·H_2_O (**11**)	(1a2m2pOH_2_)[Zn(pic)_3_]·CH_3_OH (**11a**)
empirical formula	C_17_H_23_N_3_O_6_Zn	C_22_H_26_N_4_O_8_Zn	C_23_H_28_N_4_O_8_Zn
formula weight	430.75	539.84	553.86
crystal system	triclinic	triclinic	monoclinic
space group	*P*1̅	*P*1̅	*P*2_1_/*c*
*T* [K]	150.00(10)	150.00(10)	150.00(10)
λ [Å]	0.71073	0.71073	0.71073
*a* [Å]	9.4818(2)	9.1976(2)	11.6438(4)
*b* [Å]	9.6487(3)	11.7279(3)	14.0854(4)
*c* [Å]	12.1119(3)	23.3095(5)	16.6090(5)
α [°]	66.837(3)	93.167(2)	90
β [°]	73.925(2)	92.622(2)	109.701(4)
γ [°]	87.777(2)	104.571(2)	90
*V* [Å^3^]	975.84(5)	2425.14(10)	2564.55(15)
*Z*	2	4	4
*D*_calc_ [g/cm^3^]	1.466	1.479	1.434
μ [mm^–1^]	1.296	1.067	1.011
collected reflections	48,771	60,034	26,903
unique reflections	5660	13690	6330
observed reflections	5068	10621	5044
*R*_int_	0.0397	0.0447	0.0316
*R*_1_ (*I* > 2σ(*I*))	0.0265	0.0351	0.0311
*wR*_2_ (all data)	0.0640	0.0812	0.0759

### Antibacterial Studies

Antimicrobial testing was performed using a broth microdilution method in a 96-well plate format following the CLSI guidelines and European Committee for Antimicrobial Susceptibility Testing recommendations.^66,67^ A suspension of specific bacterial strain, equivalent to 0.5 McFarland turbidity standard, was diluted with cation-adjusted Mueller Hinton broth to obtain a final inoculum of 10^5^ CFU/mL. Compounds were dissolved in DMSO at a concentration 5.12 mg/mL, and half dilutions in DMSO were prepared. DMSO solutions of compounds and inoculum were mixed together and incubated for 18–24 h at 35 °C. After incubation, the minimal inhibitory concentration (MIC) values were determined by visual inspection as the lowest dilution of compounds showing no turbidity. The MICs were determined against *S. aureus* (ATCC 29213), *E. coli* (ATCC 25922), *P. aeruginosa* (RDK 184 (DSM 939, ATCC 15442), *P. mirabilis* (RDK 064, DSM 788), *B. subtilis* (RDK 108, WDCM 00003), and *S. epidermidis* (RDK 065, WDCM 000065) bacterial strains. Tetracycline was used as a positive control on every assay plate.

### Computational Details

Geometry optimizations were performed without symmetry constraints using the ORCA 6.0 software package.^68^ Density functional theory (DFT) calculations employed the B3PW91 hybrid functional^69,70^ together with the def2-TZVP basis set^71^ and the corresponding def2/J auxiliary basis for the resolution-of-identity approximation. Empirical dispersion effects were included via the Grimme atom-pairwise D3 correction with Becke–Johnson damping (D3BJ).^72^ Bulk solvent effects (methanol) were modeled using the SMD implicit solvation model.^73^ Vibrational frequency analyses at the same level of theory were used to characterize all stationary points as minima (zero imaginary frequency) and to obtain thermodynamic corrections.

### Synthesis

#### General Method for the Synthesis of Complexes

##### Preparation of the {Zn­(pic)_2_} Precursor Solution

Zinc­(II) oxide (654 mg, 8.04 mmol) and picolinic acid (1.990 g, 16.16 mmol) were added to methanol (45 mL), and the mixture was heated under reflux conditions for 3 h. A clear, colorless solution was obtained. The solution was then cooled to an ambient temperature and weighed. The typical mass of the resulting solution was 33.66 g, corresponding to 0.239 mmol of {Zn­(pic)_2_} pergram of solution. *Note:* Occasionally, a small amount of crystalline *trans*-[Zn­(pic)_2_(CH_3_OH)_2_] (**1**) precipitated from the solution. The crystals of **1** underwent almost instantaneous decomposition when removed from the solution.

##### Analytical Data for **1**

IR (ATR, cm^–1^): 3100w, 3076w, 2982w, 2961w, 2923w, 2843w, 2806m, 1624vs, 1589vs, 1565vs, 1474m, 1446m, 1398m, 1376vs, 1293s, 1262m, 1241m, 1153m, 1128m, 1096m, 1043s, 1023vs, 971w, 923w, 847m, 823m, 758vs, 695vvs, 645s, 536s, 439s, 417s. ^1^H NMR ((CD_3_)_2_SO with 0.03% v/v TMS, 600 MHz): δ 8.40 (br s, 2H, pic^–^), 8.18 (d, *J* = 7.7 Hz, 2H, pic^–^), 8.13 (t, *J* = 7.7 Hz, 2H, pic^–^), 7.69–7.66 (m, 2H, pic^–^), 4.12 (q, *J* = 5.2 Hz, CH_3_O*H*), 3.16 (d, *J* = 5.2 Hz, C*H*_3_OH) ppm. *Note*. Owing to the inherent instability of crystals of **1**, the integrals for methanol resonances are somewhat smaller compared to theoretical values.

##### Preparation of the *Tris-chelates*

An accurately weighted portion of the {Zn­(pic)_2_} precursor solution was transferred into a round-bottom flask and combined with the desired amino alcohol in a slight excess of the 1:1 ligand-to-zinc­(II) molar ratio. This solution was heated under reflux for 3 h. Afterward, the solution was cooled to ambient temperature, and its volume was reduced to one-third using a rotary evaporator. Acetonitrile or propionitrile (3 mL) was added to the concentrate, which was placed into an Erlenmeyer flask together with a small vial filled with diethyl ether. The mixture was left to stand in a stoppered flask at ambient temperature. Within 1–14 days, depending upon the amino alcohol, crystalline product precipitated.

#### [Zn­(pic)_2_(2aeOH)] (**3**)

{Zn­(pic)_2_} precursor solution (6.96 g, 1.66 mmol Zn), 2-aminoethanol (250 mg, 4.09 mmol), propionitrile. Yield: 137 mg, 22%. Crystals were obtained on the following day. Elemental analysis calcd. for C_14_H_15_N_3_O_5_Zn (%): C, 45.36; H, 4.08; N, 11.34; found (%): C, 45.82; H, 4.36; N, 11.02. IR (ATR, cm^–1^): 3348w, 3220w, 3112m, 3069w, 3028w, 2954m, 2880m, 2835w, 2803w, 1626vs, 1591vs, 1562vs, 1480m, 1465w, 1444m, 1408w, 1387s, 1372vvs, 1335s, 1300s, 1260m, 1241m, 1171w, 1156w, 1119w, 1089w, 1052vs, 1040s, 1020s, 1001s, 981w, 886w, 868m, 847vs, 812w, 751vs, 698vvs, 637s, 521s, 429s, 411s. ^1^H NMR ((CD_3_)_2_SO with 0.03% v/v TMS, 600 MHz): δ 8.52 (br s, 2H, pic^–^), 8.20–8.11 (m, 4H, pic^–^), 7.72 (br s, 2H, pic^–^), 3.99 (br s, 2H, N*H*_2_CH_2_CH_2_OH), 3.39 (t, *J* = 5.6 Hz, 2H, NH_2_CH_2_C*H*_2_OH), 2.58 (t, *J* = 5.6 Hz, 2H, NH_2_C*H*_2_CH_2_OH) ppm.

#### [Zn­(pic)_2_(2maeOH)] (**4**)

{Zn­(pic)_2_} precursor solution (6.88 g, 1.64 mmol Zn), 2-methylaminoethanol (200 mg, 2.66 mmol), acetonitrile. Yield: 353 mg, 56%. Crystals were obtained after 3 days. Elemental analysis calcd. for C_15_H_17_N_3_O_5_Zn (%): C, 46.83; H, 4.45; N, 10.92; found (%): C, 46.32; H, 5.13; N, 11.02. ESI-HRMS: *m*/*z* calcd. for [C_15_H_18_N_3_O_5_Zn]^+^ = [Zn­(pic)_2_(2maeOH) + H]^+^: 384.0532, found: 384.0541. IR (ATR, cm^–1^): 3244m, 3195w, 3070w, 3008w, 2999w, 2965m, 2889w, 2868w, 2830w, 2736w, 2687w, 2655w, 2592w, 1622vs, 1591vs, 1566vs, 1476m, 1442m, 1433m, 1411w, 1382vs, 1352vvs, 1298m, 1291m, 1259m, 1241m, 1188w, 1164m, 1150m, 1112m, 1089m, 1066m, 1042s, 1014s, 1001s, 976s, 878w, 840s, 829s, 777s, 760vs, 699vvs, 639s, 581m, 530m, 428s, 416s. ^1^H NMR ((CD_3_)_2_SO with 0.03% v/v TMS, 600 MHz): δ 8.39 (br s, 2H, pic^–^), 8.16 (d, *J* = 7.7 Hz, 2H, pic^–^), 8.11 (t, *J* = 7.7 Hz, 2H, pic^–^), 7.67 (br s, 2H, pic^–^), 3.51 (t, *J* = 5.4 Hz, 2H, NH­(CH_3_)­CH_2_C*H*_2_OH), 2.63 (t, *J* = 5.4 Hz, 2H, NH­(CH_3_)­C*H*_2_CH_2_OH), 2.21 (s, 3H, NH­(C*H*_3_)­CH_2_CH_2_OH) ppm.

#### [Zn­(pic)_2_(2eaeOH)] (**5**)

{Zn­(pic)_2_} precursor solution (6.96 g, 1.66 mmol Zn), 2-ethylaminoethanol (250 mg, 2.80 mmol), acetonitrile. Yield: 180 mg, 27%. Crystals were obtained after 14 days. Elemental analysis calcd. for C_16_H_19_N_3_O_5_Zn (%): C, 48.20; H, 4.80; N, 10.54; found (%): C, 47.84; H, 4.54; N, 10.90. ESI-HRMS: *m*/*z* calcd. for [C_16_H_20_N_3_O_5_Zn]^+^ = [Zn­(pic)_2_(2eaeOH) + H]^+^: 398.0689, found: 398.0692. IR (ATR, cm^–1^): 3225w, 3184w, 3072w, 2976w, 2952w, 2889w, 2832w, 2738w, 1622vvs, 1589vvs, 1566vvs, 1475m, 1445m, 1415w, 1380vvs, 1350vvs, 1299s, 1290s, 1259w, 1241s, 1171m, 1151m, 1136m, 1091s, 1067s, 1043vs, 1016vs, 931w, 915w, 899w, 842s, 811m, 778s, 761vs, 700vvs, 639s, 585w, 541m, 483w, 431vs. ^1^H NMR ((CD_3_)_2_SO with 0.03% v/v TMS, 600 MHz): δ 8.36 (br s, 2H, pic^–^), 8.17 (d, *J* = 7.7 Hz, 2H, pic^–^), 8.11 (t, *J* = 7.7 Hz, 2H, pic^–^), 7.66 (br s, 2H, pic^–^), 4.85 (br s, 2H, N*H*(CH_2_CH_3_)­CH_2_CH_2_O*H*), 3.55–3.51 (m, 2H, NH­(CH_2_CH_3_)­CH_2_C*H*_2_OH), 2.72–2.68 (m, 2H, NH­(CH_2_CH_3_)­C*H*_2_CH_2_OH), 2.60 (q, *J* = 7.2 Hz, 2H, NH­(C*H*_2_CH_3_)­CH_2_CH_2_OH), 0.99 (t, *J* = 7.2 Hz, 3H, NH­(CH_2_C*H*_3_)­CH_2_CH_2_OH) ppm.

#### [Zn­(pic)_2_(2dmaeOH)] (**6**)

{Zn­(pic)_2_} precursor solution (6.92 g, 1.65 mmol Zn), 2-dimethylaminoethanol (231 mg, 2.59 mmol), propionitrile. Yield: 425 mg, 65%. Crystals were obtained after 3 days. Elemental analysis calcd. for C_16_H_19_N_3_O_5_Zn (%): C, 48.20; H, 4.80; N, 10.54; found (%): C, 48.17; H, 4.82; N, 10.62. IR (ATR, cm^–1^): 3106w, 3068w, 3060w, 3033w, 3002m, 2979w, 2962m, 2917m, 2889w, 2844w, 2815w, 2761w, 2721w, 2699w, 2668w, 2649w, 2611w, 1634vs, 1620vs, 1589vvs, 1562vvs, 1474m, 1460m, 1446m, 1415w, 1388vvs, 1364vvs, 1292m, 1255m, 1244m, 1173m, 1151w, 1100m, 1076vs, 1047vs, 1030w, 1014vs, 993w, 944s, 882s, 848vs, 774vs, 753vs, 697vvs, 637vs, 579m, 534m, 473s, 432vs, 422s. ^1^H NMR ((CD_3_)_2_SO with 0.03% v/v TMS, 600 MHz): δ 8.38 (br s, 2H, pic^–^), 8.17 (d, *J* = 7.7 Hz, 2H, pic^–^), 8.12 (t, *J* = 7.7 Hz, 2H, pic^–^), 7.69–7.65 (m, 2H, pic^–^), 5.14 (s, 1H, N­(CH_3_)_2_CH_2_CH_2_O*H*), 3.50 (t, *J* = 6.0 Hz, 2H, N­(CH_3_)_2_CH_2_C*H*_2_OH), 2.41 (t, *J* = 6.0 Hz, 2H, N­(CH_3_)_2_C*H*_2_CH_2_OH), 2.16 (s, 6H, N­(C*H*_3_)_2_CH_2_CH_2_OH) ppm.

#### Attempt to Recrystallize [Zn­(pic)_2_(2dmaeOH)] (**6**)

Compound **6** (50 mg) was dissolved in methanol (1 mL), and propionitrile (0.5 mL) was added to the resulting solution. The mixture was transferred to a small test tube, carefully layered with diethyl ether, and then sealed. After 1 week, the tube was opened, and the solvents were allowed to evaporate. Crystals of *cis*-[Zn­(pic)_2_(H_2_O)_2_]·1/2CH_3_CH_2_CN (**2**) formed on the bottom and along the walls of the tube. Elemental analysis of **2** calcd. for C_13.5_H_14.5_N_2.5_O_6_Zn (%): C, 43.45; H, 3.92; N, 9.38; found (%): C, 42.89; H, 3.65; N, 9.11. IR (ATR, cm^–1^): 3163s (broad), 2248w, 1619vvs, 1588vvs, 1566vs, 1473m, 1440m, 1370vvs, 1290s, 1261m, 1240m, 1168w, 1153w, 1139w, 1089m, 1047s, 1020s, 984s, 912w, 854s, 760vs, 698vvs, 637vs, 546vs, 528vs, 431vs, 410s. ^1^H NMR ((CD_3_)_2_SO with 0.03% v/v TMS, 600 MHz): δ 8.40 (br s, 2H, pic^–^), 8.18 (d, *J* = 7.6 Hz, 2H, pic^–^), 8.13 (t, *J* = 7.6 Hz, 2H, pic^–^), 7.69–7.66 (m, 2H, pic^–^), 2.46 (q, *J* = 7.6 Hz, 1H, CH_3_C*H*_2_CN), 1.14 (t, *J* = 7.6 Hz, 1.5H, C*H*_3_CH_2_CN) ppm.

#### [Zn­(pic)_2_(2a1pOH)]·H_2_O (**7**)

{Zn­(pic)_2_} precursor solution (3.49 g, 0.833 mmol Zn), 2-amino-1-propanol (150 mg, 2.00 mmol), and acetonitrile. Yield: 160 mg, 48%. Crystals of **7** were obtained after 4 days. *Note:* When the crystals of **7** were left in the reaction mixture for 3 weeks, a new compound precipitated. Single-crystal X-ray diffraction revealed its composition to be the anhydrous complex [Zn­(pic)_2_(2a1pOH)] (**8**). As compound **8** could not be isolated in a pure form, no further characterization was possible.

##### Analytical Data for **7**

Elemental analysis calcd. for C_15_H_19_N_3_O_6_Zn (%): C, 44.74; H, 4.76; N, 10.43; found (%): C, 44.86; H, 4.35; N, 10.52. IR (ATR, cm^–1^): 3480w, 3255w, 3173w, 3069w, 2964w, 2936w, 2884w, 1622vs, 1590vvs, 1565vs, 1473w, 1463w, 1441w, 1407w, 1373vvs, 1291s, 1262m, 1242m, 1222m, 1169w, 1150m, 1119s, 1092w, 1076w, 1042vs, 1020s, 927w, 839s, 763vs, 700vvs, 639vs, 580w, 563w, 527s, 430s, 415s. ^1^H NMR ((CD_3_)_2_SO with 0.03% v/v TMS, 600 MHz): δ 8.51 (br s, 2H, pic^–^), 8.17 (d, *J* = 7.5 Hz, 2H, pic^–^), 8.14–8.10 (m, 2H, pic^–^), 7.70 (br s, 2H, pic^–^), 4.15 (br s, 2H, N*H*_2_CH­(CH_3_)­CH_2_OH), 3.40–3.29 (m, 2H, NH_2_CH­(CH_3_)­C*H*_2_OH), 3.13 (t, *J* = 9.2 Hz, 1H, NH_2_CH­(CH_3_)­CH_2_O*H*), 2.86–2.80 (m, 1H, NH_2_C*H*(CH_3_)­CH_2_OH), 0.94 (d, *J* = 6.4 Hz, 3H, NH_2_CH­(C*H*_3_)­CH_2_OH) ppm.

#### [Zn­(pic)_2_(1a2bOH)]·CH_3_CN (**9**)

{Zn­(pic)_2_} precursor solution (4.61 g, 1.10 mmol Zn), 1-amino-2-butanol (280 mg, 3.14 mmol), and acetonitrile. Crystals of two morphologies were obtained after 10 days: prisms of [Zn­(pic)_2_(1a2bOH)]·CH_3_CN (**9**) and clusters of very fragile, needle-like crystals **9a**. The crystals were separated manually using an optical microscope. In the air, the crystals of **9** slowly release acetonitrile molecules of crystallization.

##### Analytical Data for **9**

Elemental analysis of [Zn­(pic)_2_(1a2bOH)] calcd. for C_16_H_19_N_3_O_5_Zn (%): C, 48.20; H, 4.80; N, 10.54; found (%): C, 47.79; H, 4.74; N, 10.68. IR (ATR, cm^–1^): 3258m, 3166m, 3115w, 2963m, 2934m, 2880w, 2251w, 1631vs, 1595vs, 1568s, 1477m, 1461w, 1443m, 1357vs, 1299m, 1261m, 1238m, 1169m, 1147m, 1114m, 1090m, 1062w, 1048s, 1020s, 982s, 920w, 845vs, 790w, 755vs, 698vvs, 641vs, 536m, 501w, 431vs. ^1^H NMR ((CD_3_)_2_SO with 0.03% v/v TMS, 600 MHz): δ 8.50 (br s, 2H, pic^–^), 8.17 (d, *J* = 7.6 Hz, 2H, pic^–^), 8.13 (t, *J* = 7.6 Hz, 2H, pic^–^), 7.71 (br s, 2H, pic^–^), 3.86 (br s, 2H, N*H*_2_CH_2_CH­(CH_2_CH_3_)­OH), 3.37–3.32 (m, 2H, NH_2_CH_2_C*H*(CH_2_CH_3_)­O*H*), 2.57 (dd, *J* = 12.5, 3.5 Hz, 1H, NH_2_C*H*_2_CH­(CH_2_CH_3_)­OH), 2.35 (dd, *J* = 12.5, 8.4 Hz, 1H, NH_2_C*H*_2_CH­(CH_2_CH_3_)­OH), 2.07 (s, 3H, C*H*_3_CN), 1.35–1.24 (m, 2H, NH_2_CH_2_CH­(C*H*_2_CH_3_)­OH), 0.78 (t, *J* = 7.5 Hz, 3H, NH_2_CH_2_CH­(CH_2_C*H*_3_)­OH) ppm.

##### Analytical Data for **9a**

Elemental analysis of [Zn­(pic)_2_(1a2bOH)]·H_2_O calcd. for C_16_H_21_N_3_O_6_Zn (%): C, 46.11; H, 5.08; N, 10.08; found (%): C, 45.88; H, 4.92; N, 10.43. IR (ATR, cm^–1^): 3353m, 3245m, 3156m, 3072w, 2969m, 2936m, 2878w, 2249w, 1630vs, 1591vs, 1565s, 1475m, 1462m, 1446m, 1377vs, 1347vvs, 1291m, 1260m, 1239m, 1169m, 1151w, 1120w, 1091m, 1046vs, 1019s, 979s, 927m, 883w, 846vs, 838vs, 763vs, 701vvs, 668w, 639vs, 528m, 433vs, 414s. *Note*. ^1^H NMR spectrum for **9a** is the same as for **9.**

#### [Zn­(pic)_2_(1a2m2pOH)]·CH_3_OH (**10**)

{Zn­(pic)_2_} precursor solution (3.95 g, 0.943 mmol Zn), 1-amino-2-methyl-2-propanol (132 mg, 1.48 mmol), and acetonitrile. Crystals of **10** were obtained after 6 days. *Notes*. The crystals of **10** decomposed almost immediately upon removal from the mother liquor. The compound loses methanol molecules of crystallization and binds moisture from the air. A small quantity of stable crystals with a different morphology was also observed. Single-crystal X-ray diffraction identified these as (1a2m2pOH_2_)­[Zn­(pic)_3_]·H_2_O (**11**). Since compound **11** could not be isolated in pure form, no further characterization was possible.

##### Analytical Data for **10**

Elemental analysis for [Zn­(pic)_2_(1a2m2pOH)]·H_2_O calcd. for C_16_H_21_N_3_O_6_Zn (%): C, 46.11; H, 5.08; N, 10.08; found (%): C, 45.56; H, 5.09; N, 10.01. IR (ATR, cm^–1^): 3373w, 3282w, 3176w, 3103w, 3076w, 3031w, 2963w, 2817w, 1625vvs, 1591vvs, 1566vvs, 1475m, 1446m, 1408m, 1372vvs, 1292m, 1262m, 1242m, 1211m, 1171w, 1149m, 1095s, 1045vs, 1035vs, 1019s, 994w, 969m, 932w, 909m, 899m, 842s, 807w, 761vs, 699vvs, 639vs, 586w, 559m, 535m, 430s, 416s. ^1^H NMR ((CD_3_)_2_SO with 0.03% v/v TMS, 600 MHz): δ 8.49 (br s, 2H, pic^–^), 8.17 (d, *J* = 7.7 Hz, 2H, pic^–^), 8.14–8.08 (m, 2H, pic^–^), 7.69 (br s, 2H, pic^–^), 4.10 (s, 1H, CH_3_O*H*), 3.17 (s, 3H, C*H*_3_OH), 2.43 (s, 2H, NH_2_C*H*_2_C­(CH_3_)_2_OH), 1.06 (s, 6H, NH_2_CH_2_C­(C*H*_3_)_2_OH) ppm.

#### Reaction of **10** with Picolinic Acid

Dried [Zn­(pic)_2_(1a2m2pOH)] (95 mg) was added with stirring to the methanol (4 mL) solution of picolinic acid (100 mg). Within a couple of minutes, all the solid was consumed. Diethyl ether was added dropwise until the solution turned turbid. The resulting solution was stored overnight in the refrigerator. On the following day, a substantial amount of colorless crystals of (1a2m2pOH_2_)­[Zn­(pic)_3_]·CH_3_OH (**11a**) was obtained. Yield: 74 mg. The crystals of **11a** turned opaque almost immediately after being removed from the mother liquor. The change in the appearance is due to the loss of methanol solvent molecules. Elemental analysis for (1a2m2pOH_2_)­[Zn­(pic)_3_]·2H_2_O calcd. for C_22_H_28_N_4_O_9_Zn (%): C, 47.37; H, 5.06; N, 10.04; found (%): C, 47.56; H, 5.10; N, 10.05. IR (opaque crystals) (ATR, cm^–1^): 3328br, 3072w, 2972m, 2938w, 2824w, 2693w, 2612w, 1618vs, 1589vvs, 1565vvs, 1472m, 1443m, 1373vvs, 1290s, 1261m, 1240m, 1212m, 1171m, 1148m, 1090m, 1046s, 1032m, 1019s, 967m, 897w, 847s, 757s, 700vvs, 639s, 553m, 526m, 430s. ^1^H NMR ((CD_3_)_2_SO with 0.03% v/v TMS, 600 MHz): δ 8.19 (d, *J* = 5.0 Hz, 3H, pic^–^), 8.14 (d, *J* = 7.7 Hz, 3H, pic^–^), 8.08–8.04 (m, 3H, pic^–^), 7.74 (br s, 3H, ^+^N*H*_3_CH_2_C­(CH_3_)_2_OH), 7.62–7.59 (m, 3H, pic^–^), 5.03 (s, 1H, ^+^NH_3_CH_2_C­(CH_3_)_2_O*H*), 2.70 (s, 2H, ^+^NH_3_C*H*_2_C­(CH_3_)_2_OH), 1.13 (s, 6H, ^+^NH_3_CH_2_C­(C*H*_3_)_2_OH) ppm.

## Supplementary Material





## Notes

X-ray structure analysis (Figures S1–S21 and Table S1), IR spectra of **1**–**11a** (Figures S22 and S33), ^1^H NMR spectra of **1**–**11a** (Figures S34–S43), and theoretical calculations (*XYZ* coordinates) (PDF)
DFT geometries and energies (XYZ)
