# Supplementary material for: Exploring Zinc(II) Coordination Chemistry with Picolinate and Amino Alcohols: Toward New Antibacterial Agents
Source: ACS Omega. 2026 May 25;11(22):32955–70. doi: 10.1021/acsomega.6c02474 (PMC13261601; doi:10.1021/acsomega.6c02474)
Supplement: Supplementary file 1 [file ao6c02474_si_001.pdf]

Supporting Information
for

Exploring zinc(II) coordination chemistry with picolinate and amino alcohols: towards new antibacterial agents

Barbara Modec,[a,*] Nina Podjed Rihtaršič,[a] Joaquín López-Serrano,[b] Martina Hrast Rambaher[c] and Majda Golob[d]

[a] Faculty of Chemistry and Chemical Technology, University of Ljubljana, Večna pot 113
1000 Ljubljana, Slovenia

[b] Instituto de Investigaciones Químicas (IIQ), Departamento de Química Inorgánica and Centro de Innovación en Química Avanzada (ORFEO-CINQA), Consejo Superior de Investigaciones Científicas (CSIC) and Universidad de Sevilla, Avenida Américo Vespucio 49, 41092 Sevilla, Spain

[c] Faculty of Pharmacy, University of Ljubljana, Aškerčeva 7, Ljubljana, Slovenia.

[d] Institute of Microbiology and Parasitology, Veterinary Faculty, University of Ljubljana, Gerbičeva 60, Ljubljana, Slovenia.

* Corresponding author: barbara.modec@fkkt.uni-lj.si

Contents

1. X-ray structure analysis
2. Infrared spectra
3. ^{1}H NMR spectra
4. DFT calculations

1. X-ray structure analysis

Figure S1. ORTEP drawing of [Zn(pic)$_2$(2aeOH)] (**3**), with thermal ellipsoids at the 50% probability level. For clarity, one component of a disordered amino alcohol ligand is drawn.

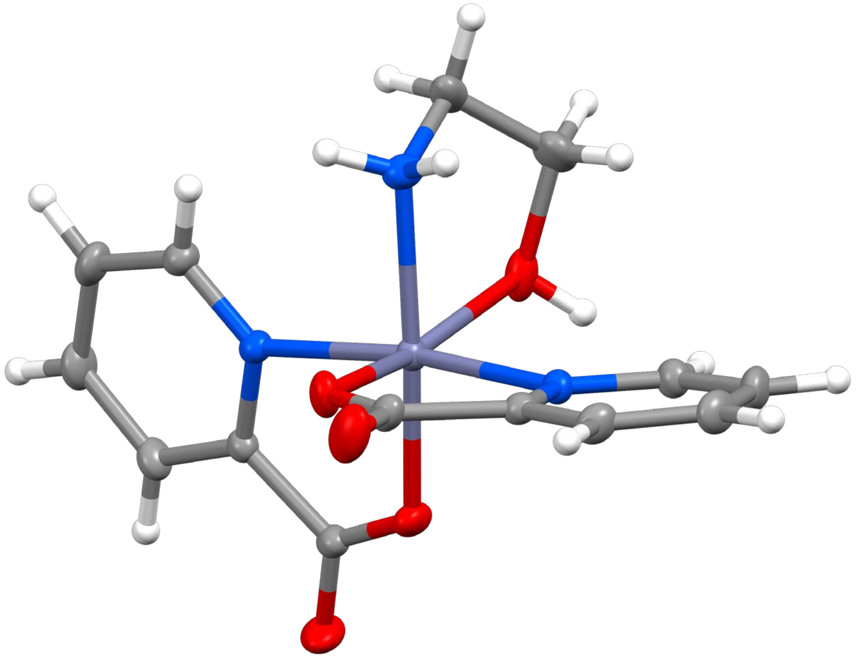

Figure S2. ORTEP drawing of [Zn(pic)$_2$(2eaeOH)] (**5**), with thermal ellipsoids at the 50% probability level.

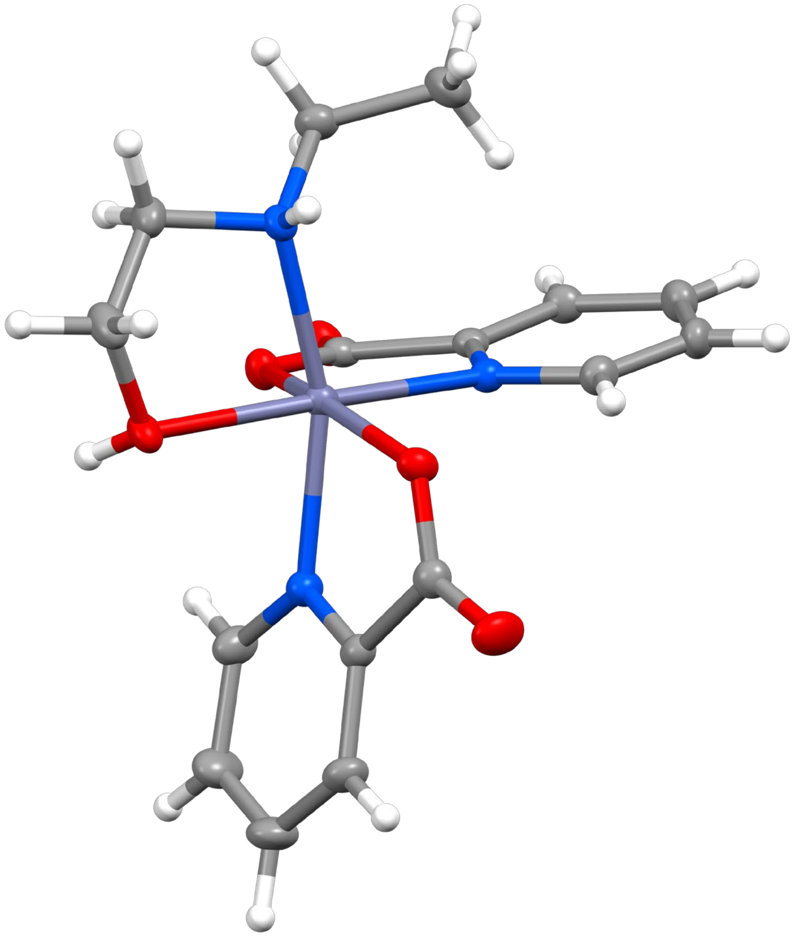

Figure S3. ORTEP drawing of [Zn(pic)$_2$(2dmaeOH)] (**6**), with thermal ellipsoids at the 50% probability level.

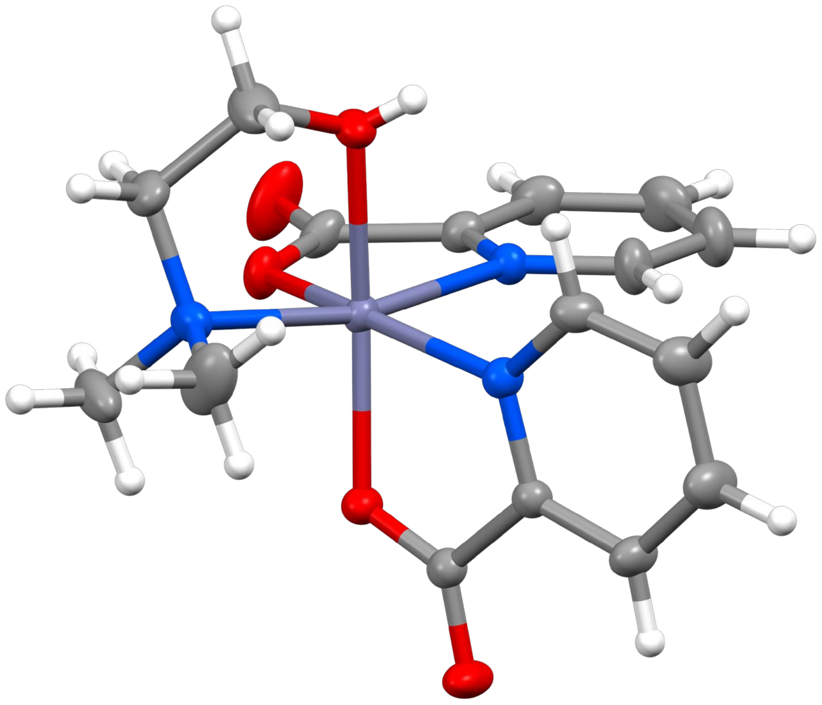

Figure S4. ORTEP drawing of [Zn(pic)$_2$(2a1pOH)], a complex molecule in **7**, with thermal ellipsoids at the 50% probability level.

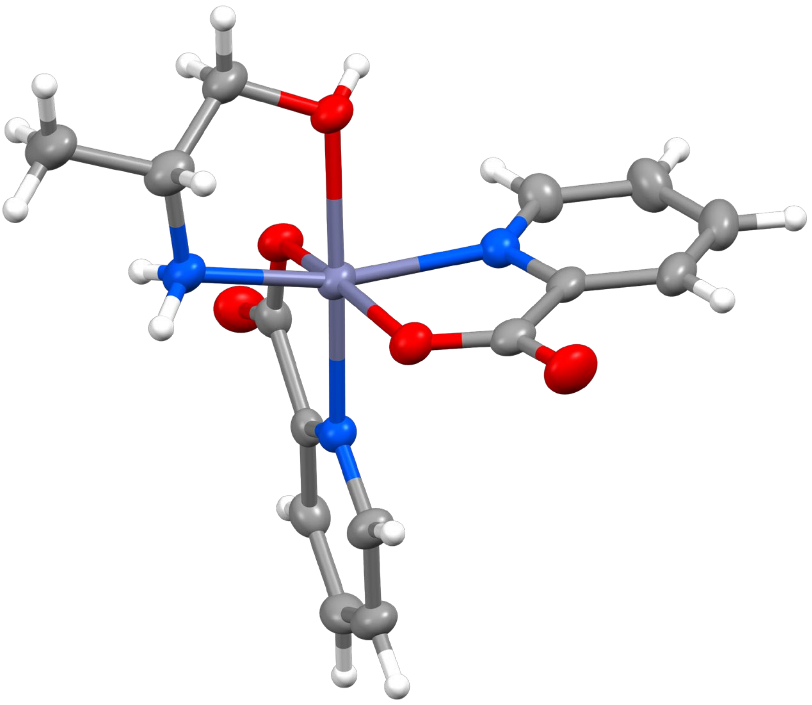

Figure S5. ORTEP drawing of [Zn(pic)$_2$(2a1pOH)] (**8**), with thermal ellipsoids at the 50% probability level.

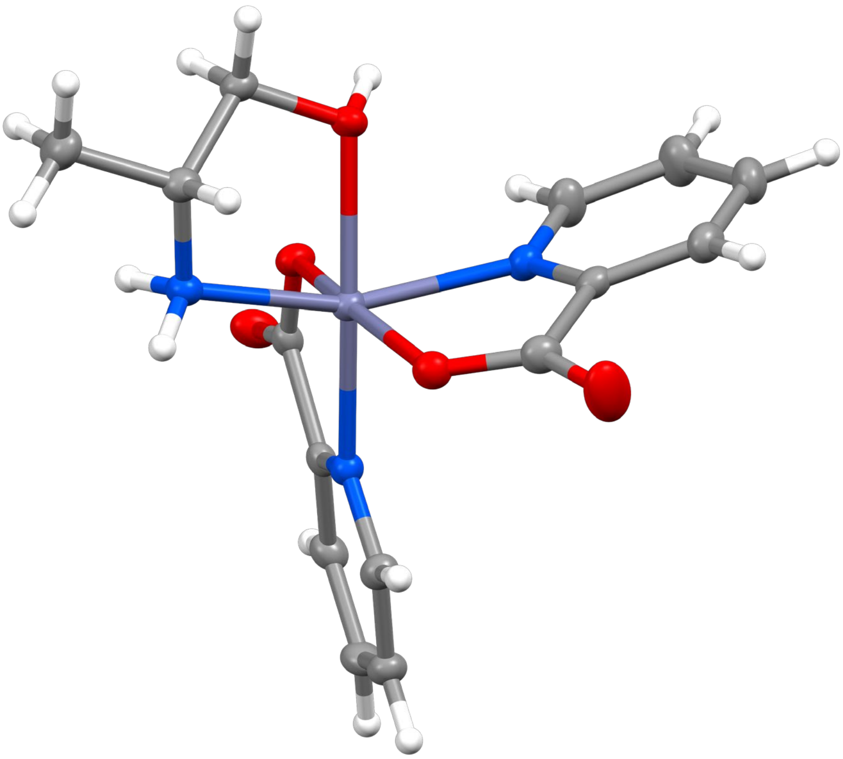

Figure S6. ORTEP drawing of [Zn(pic)$_2$(1a2bOH)], a complex molecule in **9**, with thermal ellipsoids at the 50% probability level. For clarity, one component of the disordered ethyl group of the amino alcohol is drawn.

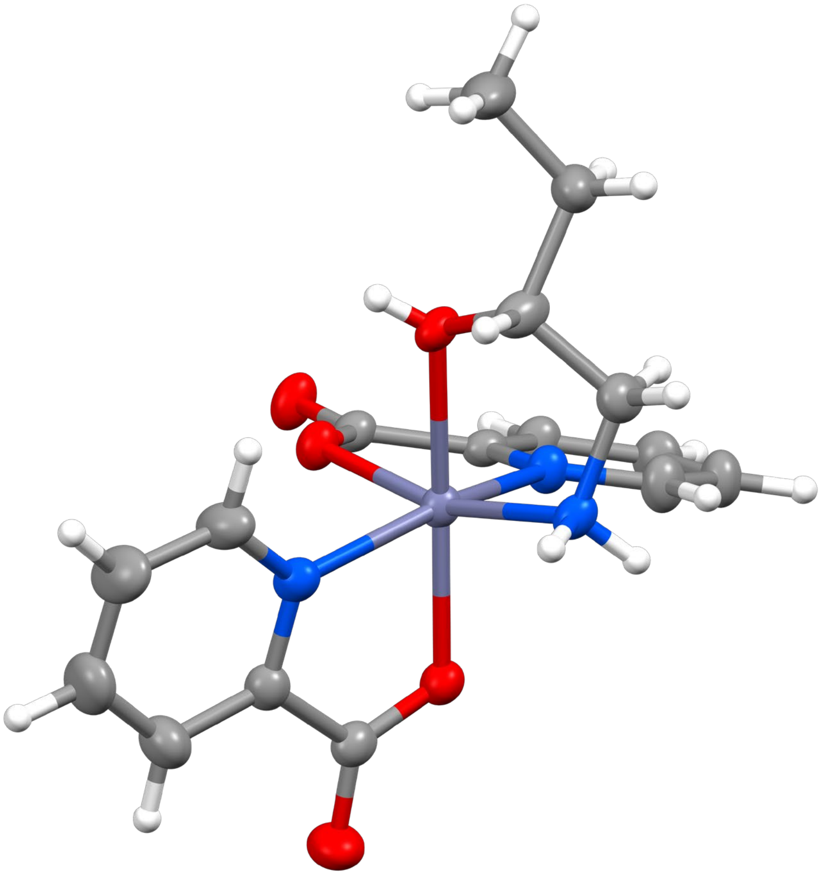

Figure S7. ORTEP drawing of [Zn(pic)$_2$(1a2m2pOH)], a complex molecule in **10**, with thermal ellipsoids at the 50% probability level.

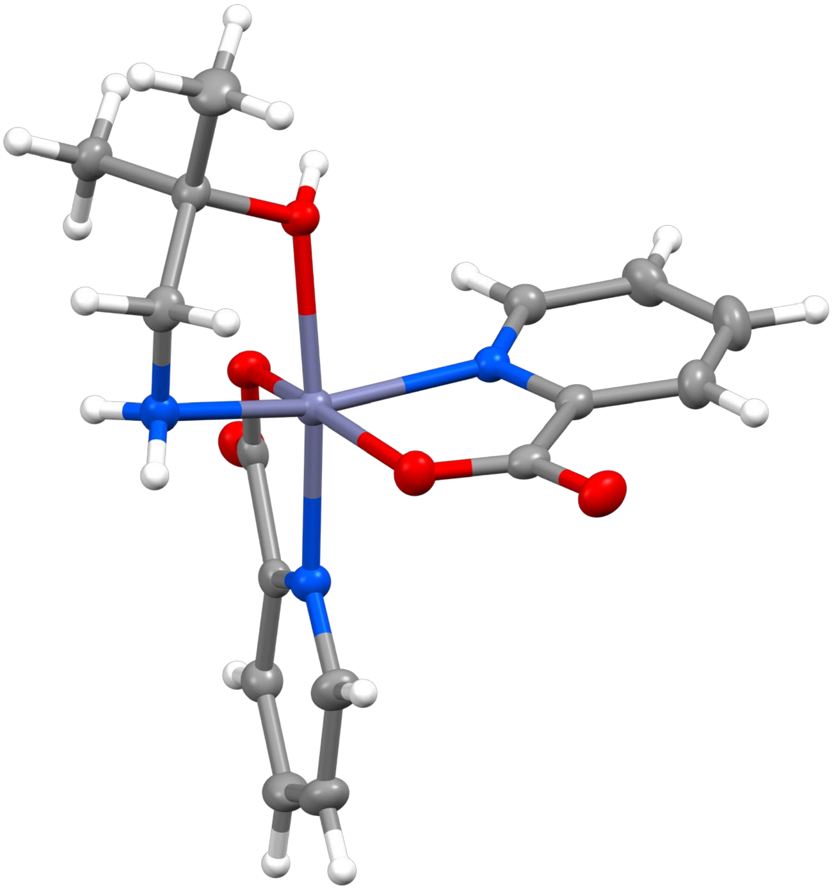

Figure S8. ORTEP drawing of $[Zn(pic)_3]^-$, a complex anion in $(1a2m2pOH_2)[Zn(pic)_3]\cdot CH_3OH$ (**11a**) with thermal ellipsoids at the 50% probability level.

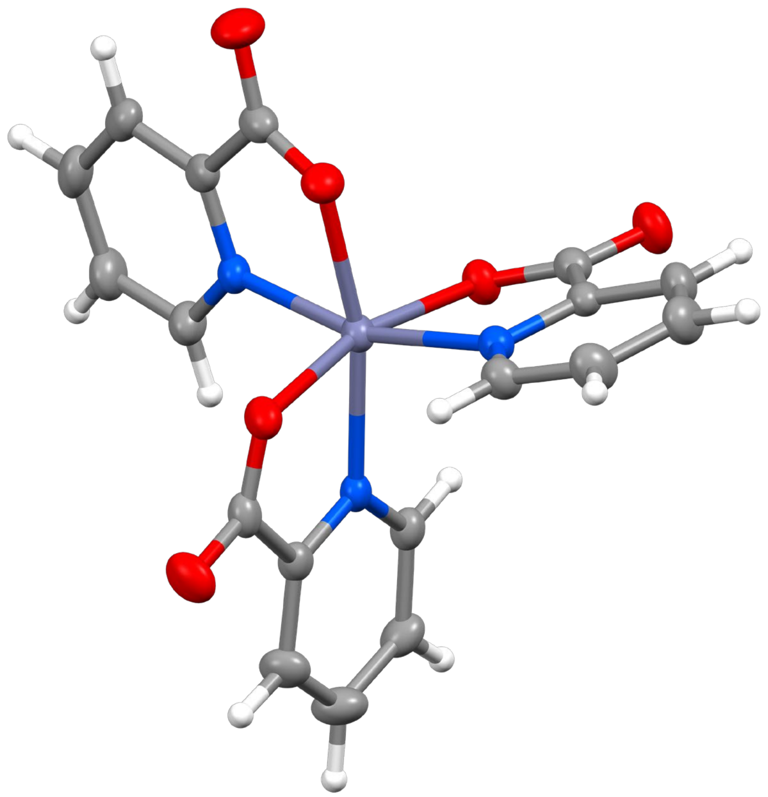

Table S1. Hydrogen bond parameters for **1–11a**.

**Compound**	**Hydrogen bond**	**D···A distance [Å]**
trans-[Zn(pic)$_2$(CH$_3$OH)$_2$] (**1**)	$CH_3OH\cdots COO^-$	O···O [1–*x*, 2–*y*, 1–*z*] = 2.6544(16)
cis-[Zn(pic)$_2$(H$_2$O)$_2$]·1/2CH$_3$CH$_2$CN (**2**)	$H_2O\cdots COO^-$	O···O [1.5–*x*, 1.5–*y*, 0.5–*z*] = 2.6910(17)
	$H_2O\cdots COO^-$	O···O [1–*x*, –0.5+*y*, 0.5–*z*] = 2.6991(18)
	$H_2O\cdots COO^-$	O···O [1–*x*, –0.5+*y*, 0.5–*z*] = 2.6904(17)
	$H_2O\cdots COO^-$	O···O [–0.5+*x*, 1–*y*, *z*] = 2.7125(16)
[Zn(pic)$_2$(2aeOH)] (**3**)	$OH\cdots COO^-$	O···O [*x*, –0.5+*y*, –0.5+*z*] = 2.852(11)
	$NH_2\cdots COO^-$	N···O [0.5–*x*, 1–*y*, –0.5+*z*] = 2.670(14)
[Zn(pic)$_2$(2maeOH)] (**4**)	$OH\cdots COO^-$	O···O [1–*x*, 1–*y*, 1–*z*] = 2.6370(16)
[Zn(pic)$_2$(2eaeOH)] (**5**)	$OH\cdots COO^-$	O···O [1–*x*, 1–*y*, 1–*z*] = 2.6582(15)
	$NH\cdots COO^-$	N···O [1–*x*, 1–*y*, –*z*] = 2.9914(17)
[Zn(pic)$_2$(2dmaeOH)] (**6**)	$OH\cdots COO^-$	O···O [*x*, *y*, 1+*z*] = 2.632(4)
[Zn(pic)$_2$(2a1pOH)]·H$_2$O (**7**)	$OH\cdots COO^-$	O···O [1–*x*, 1–*y*, 1–*z*] = 2.638(3)
	$NH_2\cdots H_2O$	N···O = 2.810(6)
	$H_2O\cdots COO^-$	O···O [–1+*x*, –1+*y*, *z*] = 2.659(6)
	$H_2O\cdots H_2O$	O···O [–*x*, –*y*, –*z*] = 2.776(15)
[Zn(pic)$_2$(2a1pOH)] (**8**)	$OH\cdots COO^-$	O···O [1.5–*x*, 0.5+*y*, 0.5–*z*] = 2.6783(13)
[Zn(pic)$_2$(1a2bOH)]·CH$_3$CN (**9**)	$OH\cdots COO^-$	O···O [1–*x*, 2–*y*, 1–*z*] = 2.729(2)
	$NH_2\cdots COO^-$	N···O [1–*x*, 1–*y*, 1–*z*] = 3.066(2)
	$NH_2\cdots COO^-$	N···O [1+*x*, *y*, *z*] = 2.990(2)
[Zn(pic)$_2$(1a2m2pOH)]·CH$_3$OH (**10**)	$OH\cdots COO^-$	O···O [1–*x*, 2–*y*, 1–*z*] = 2.6765(14)
	$CH_3OH\cdots COO^-$	O···O = 2.7534(15)
(1a2m2pOH$_2$)[Zn(pic)$_3$]·H$_2$O (**11**)	$OH\cdots COO^-$	O···O [1–*x*, 1–*y*, 1–*z*] = 2.815(2)
	$OH\cdots H_2O$	O···O = 2.764(2)
	$NH_3^+\cdots COO^-$	N···O [–1+*x*, *y*, *z*] = 2.983(2)
	$NH_3^+\cdots COO^-$	N···O = 2.903(2)
	$NH_3^+\cdots COO^-$	N···O = 2.741(2)
	$NH_3^+\cdots COO^-$	N···O [1+*x*, *y*, *z*] = 2.9258(19)
	$NH_3^+\cdots COO^-$	N···O [1+*x*, *y*, *z*] = 2.901(2)
	$NH_3^+\cdots COO^-$	N···O [1+*x*, 1+*y*, *z*] = 2.886(2)
	$NH_3^+\cdots COO^-$	N···O [*x*, 1+*y*, *z*] = 2.8392(19)
	$H_2O\cdots COO^-$	O···O [1–*x*, 1–*y*, 1–*z*] = 2.925(3)
	$H_2O\cdots COO^-$	O···O = 2.883(2)
	$H_2O\cdots COO^-$	O···O [2–*x*, 1–*y*, –*z*] = 2.8610(19)
	$H_2O\cdots COO^-$	O···O = 2.732(2)
(1a2m2pOH$_2$)[Zn(pic)$_3$]·CH$_3$OH (**11a**)	$OH\cdots COO^-$	O···O [1+*x*, 0.5–*y*, 0.5+*z*] = 2.9041(18)
	$NH_3^+\cdots COO^-$	N···O = 2.706(2)
	$NH_3^+\cdots COO^-$	N···O [*x*, 0.5–*y*, 0.5+*z*] = 2.8222(19)
	$NH_3^+\cdots CH_3OH$	N···O [1–*x*, 1–*y*, 1–*z*] = 2.858(2)
	$CH_3OH\cdots COO^-$	O···O = 2.7392(19)

Figure S9. A supramolecular chain in the structure of *trans*-[Zn(pic)$_2$(CH$_3$OH)$_2$] (**1**).

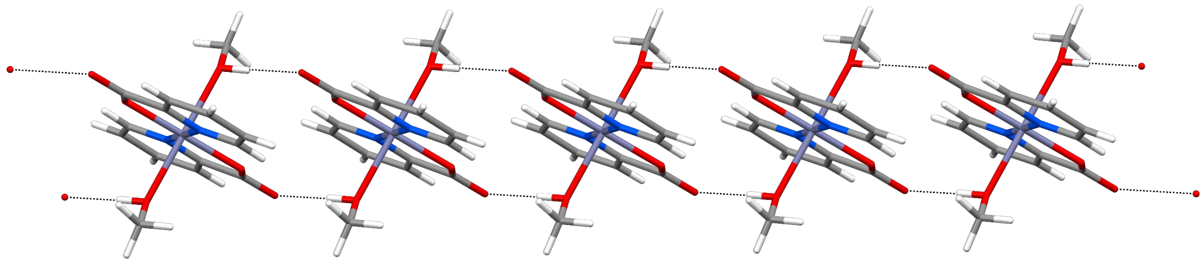

Figure S10. Packing of supramolecular chains in the structure of *trans*-[Zn(pic)$_2$(CH$_3$OH)$_2$] (**1**), a view along the chains. Each molecule is surrounded by six other molecules.

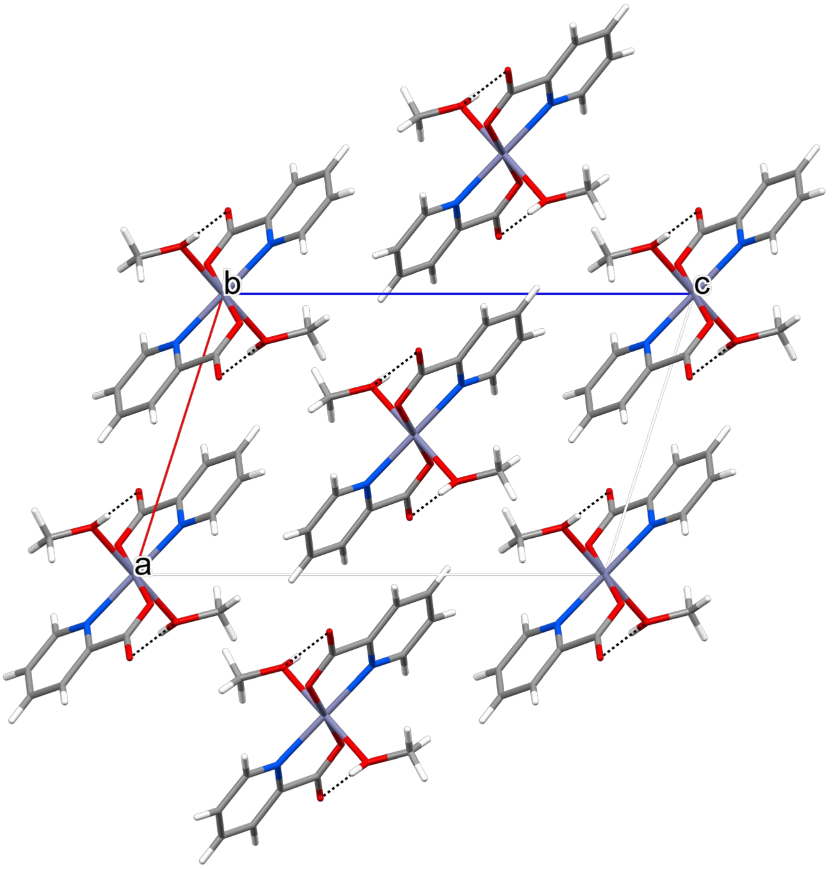

Figure S11. A view along the supramolecular layer in the structure of *cis*-[Zn(pic)$_2$(H$_2$O)$_2$]·1/2CH$_3$CH$_2$CN (**2**). Propionitrile molecules of crystallisation are not shown.

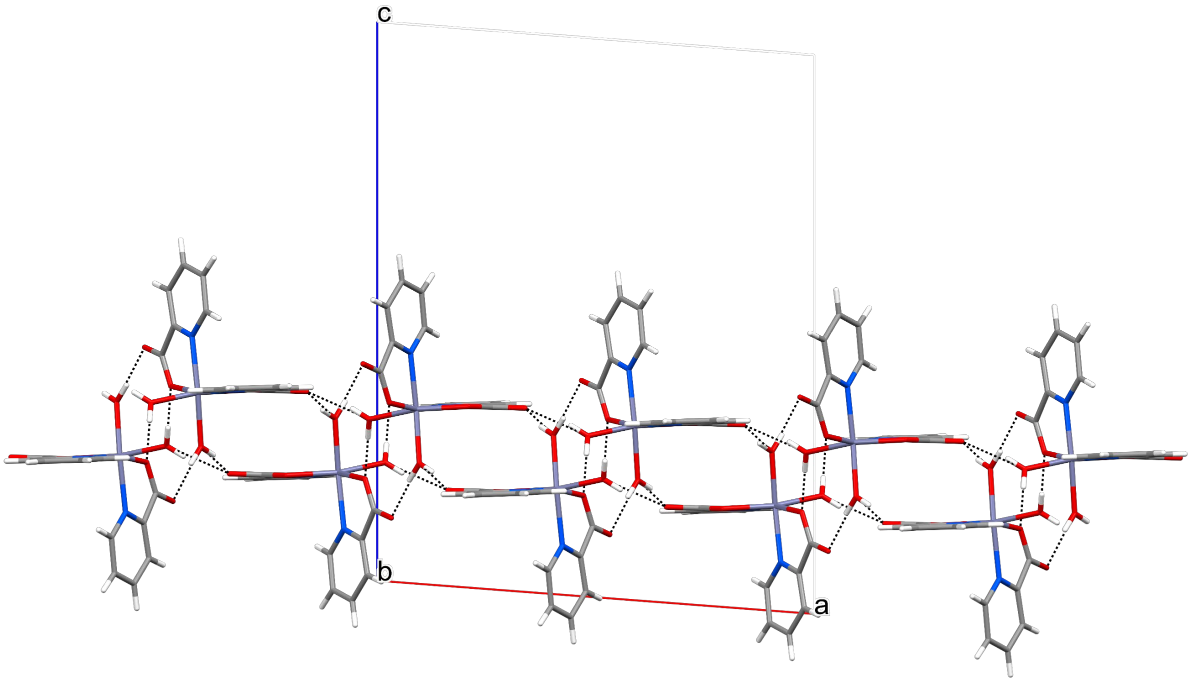

Figure S12. Stacking of layers along *c*-axis in the structure of *cis*-[Zn(pic)$_2$(H$_2$O)$_2$]·1/2CH$_3$CH$_2$CN (**2**). Propionitrile molecules of crystallisation are not shown.

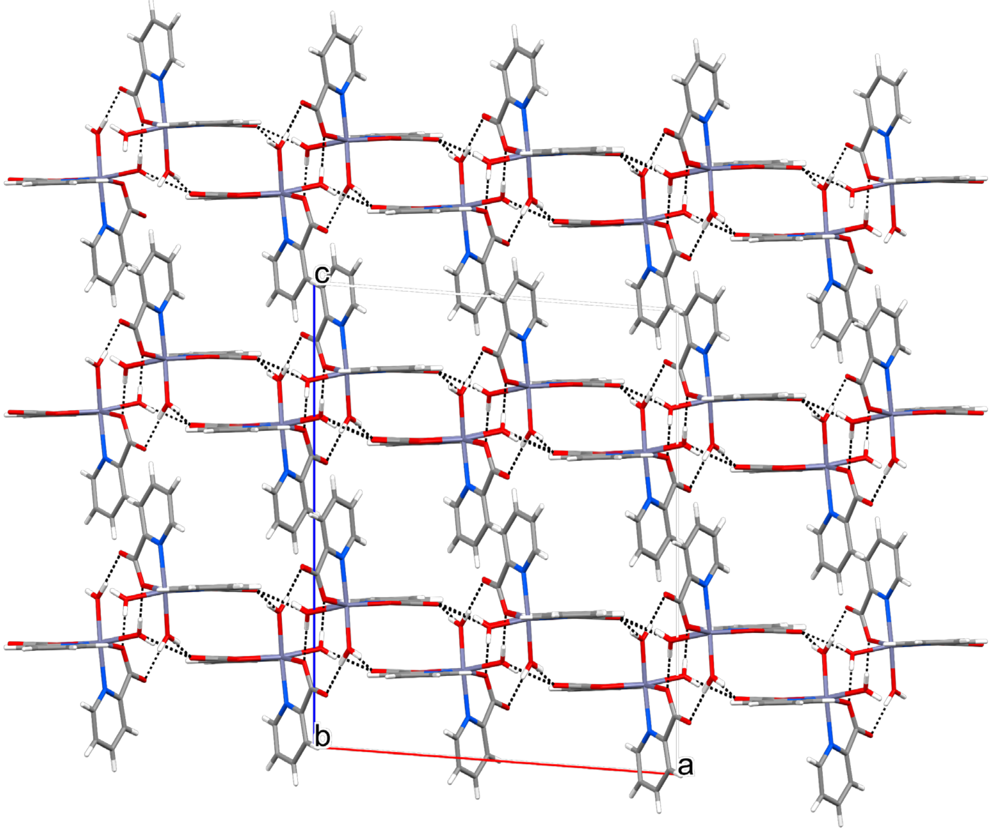

Figure S13. A view perpendicular to the supramolecular layer in the structure of [Zn(pic)$_2$(2aeOH)] (**3**). The layers stack along *a* axis.

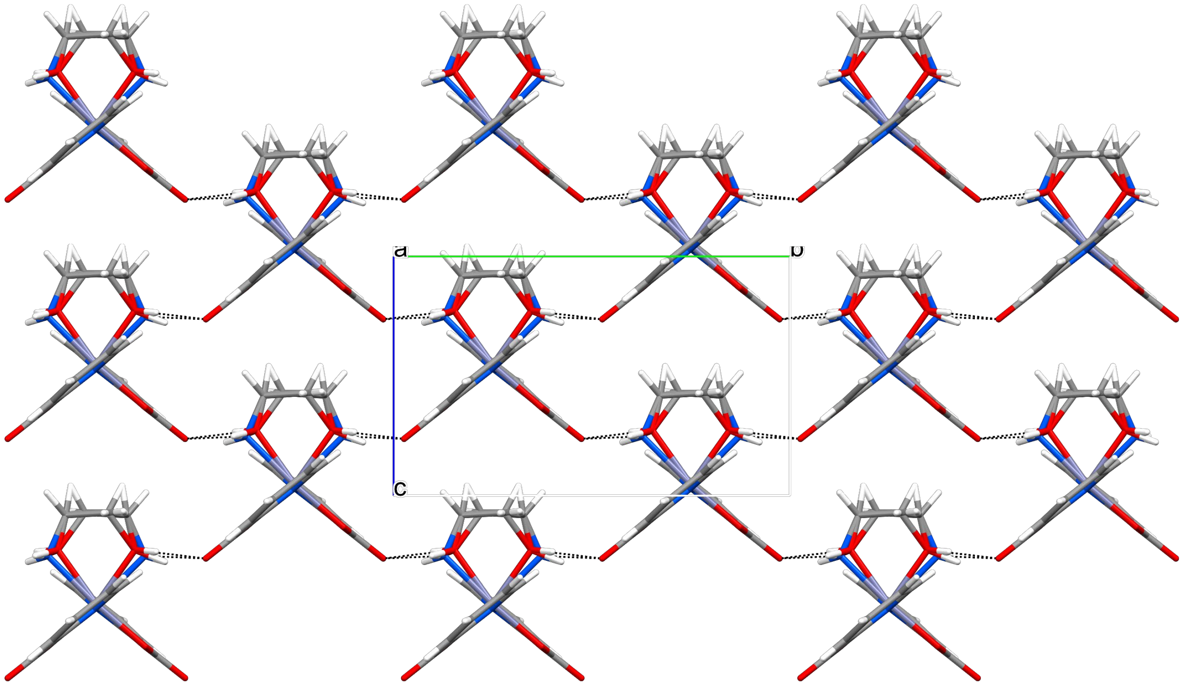

Figure S14. Hydrogen-bonding pattern in the structure of [Zn(pic)$_2$(2maeOH)] (**4**): OH···COO$^-$ hydrogen bonds link a pair of complex molecules.

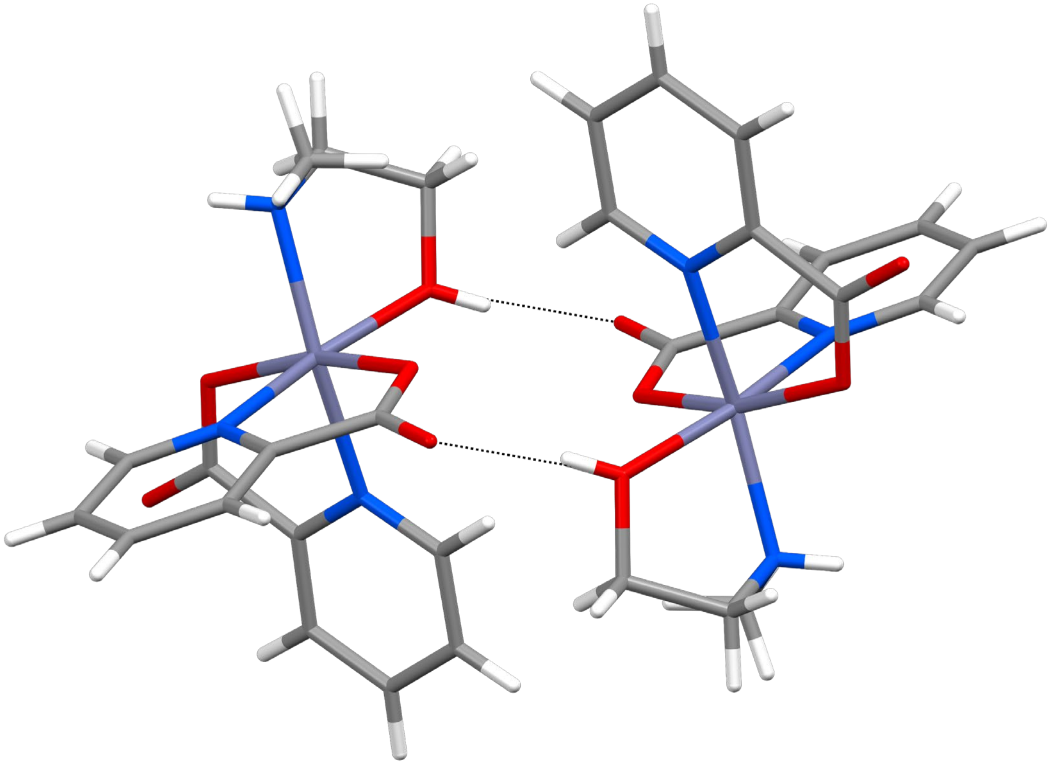

Figure S15. A supramolecular chain in the structure of [Zn(pic)$_2$(2eaeOH)] (**5**).

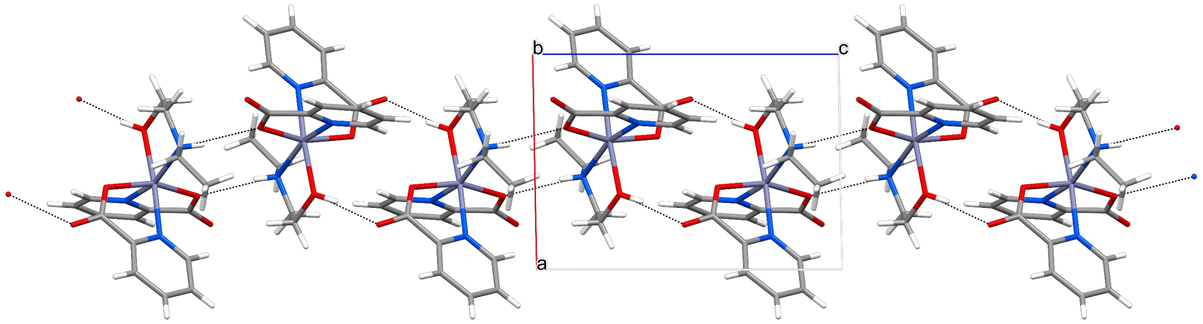

Figure S16. Packing of chains in the structure of [Zn(pic)$_2$(2eaeOH)] (**5**), a view along the chains. Each chain is surrounded by six others.

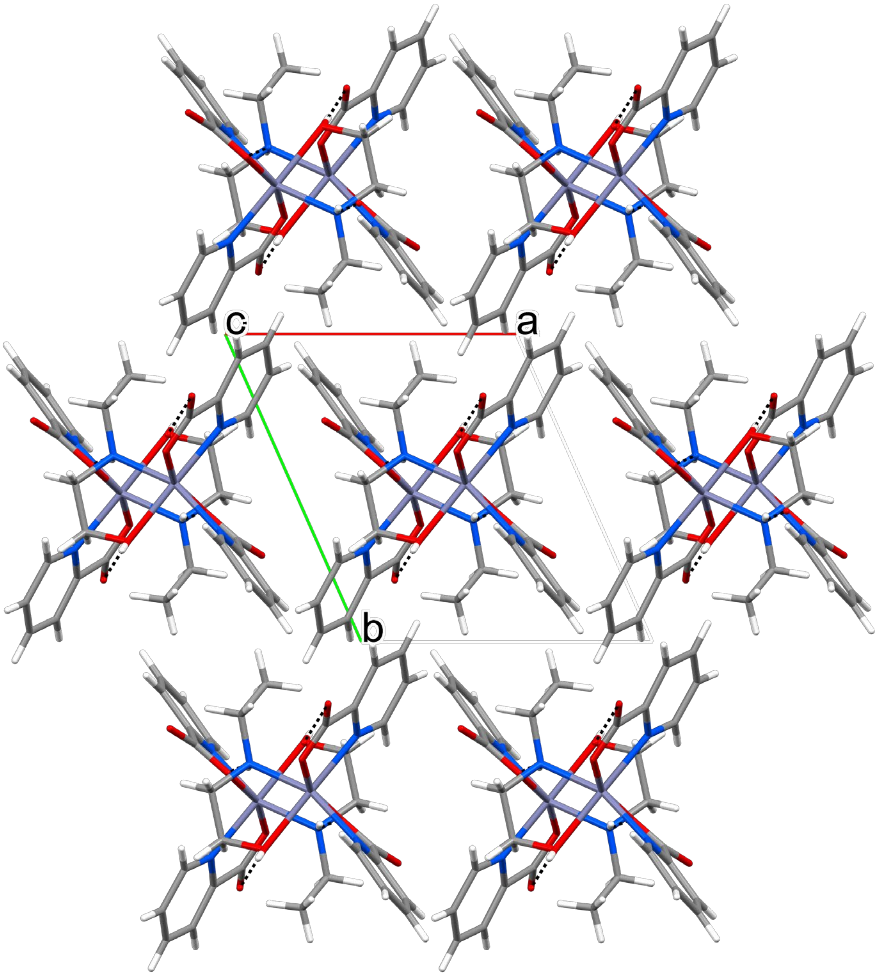

Figure S17. A supramolecular chain in the structure of [Zn(pic)$_2$(2dmaeOH)] (**6**).

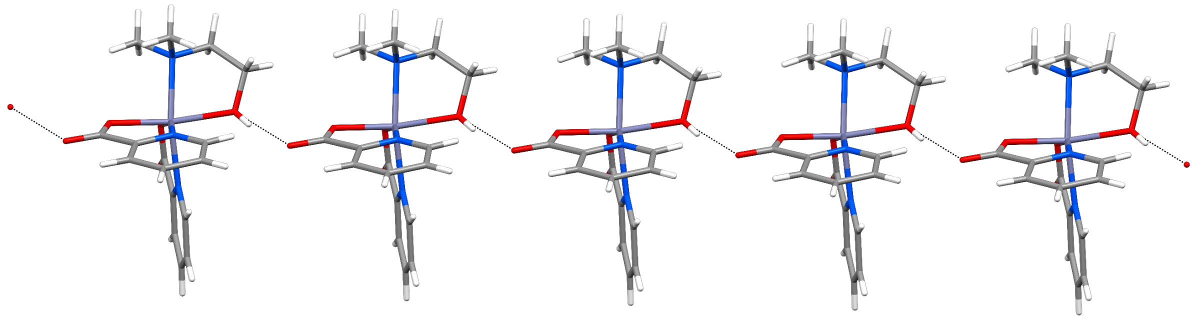

Figure S18. Packing diagram for the structure of [Zn(pic)$_2$(1a2bOH)]·CH_3CN (**9**), a view along the layers. Colour code: grey – complex molecules, and blue – acetonitrile molecules of crystallisation.

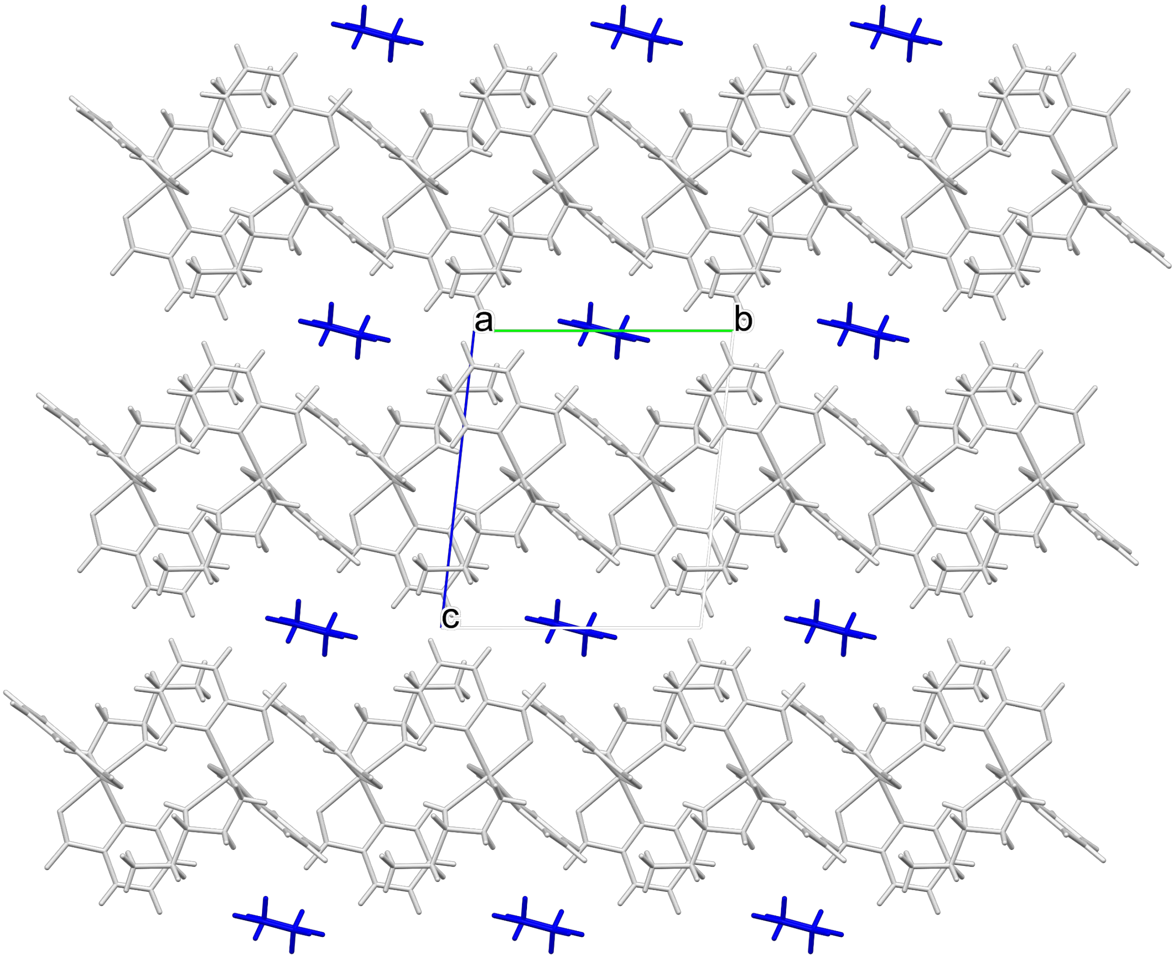

Figure S19. Hydrogen-bonding pattern in the structure of $[Zn(pic)_2(1a2m2pOH)]\cdot CH_3OH$ (**10**): hydrogen bonds of the OH···COO⁻ type link complex molecules into a dimer. Methanol molecules are attached to this dimer.

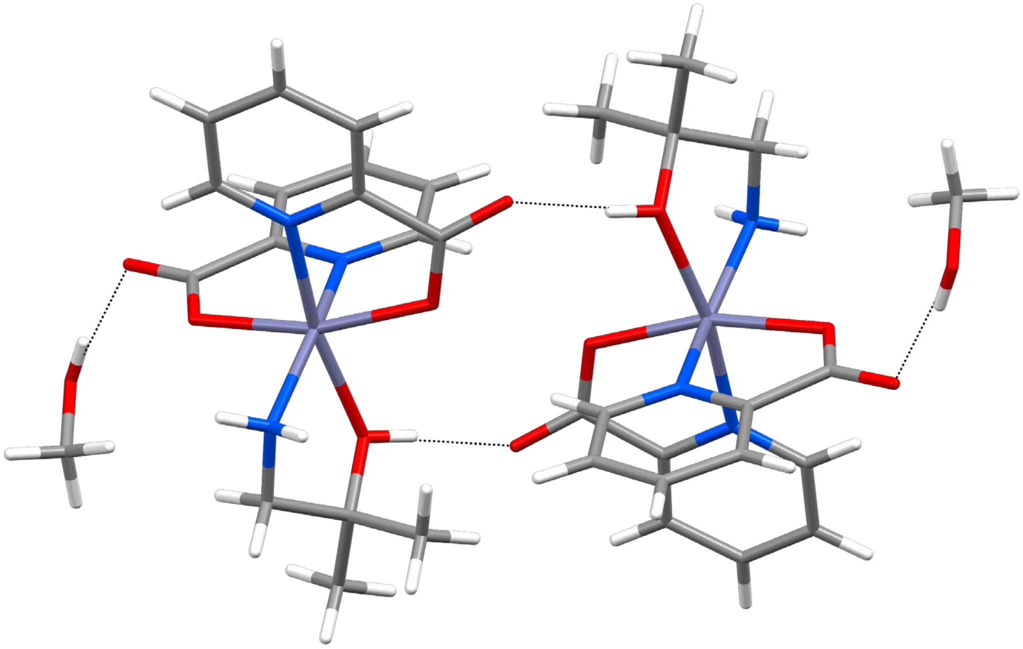

Figure S20. Packing diagram for the structure of (1a2m2pOH$_2$)[Zn(pic)$_3$]·H_2O (**11**). Colour code: grey – complex molecules, blue – 1a2m2pOH$_2^+$ cations, and red – water molecules of crystallisation.

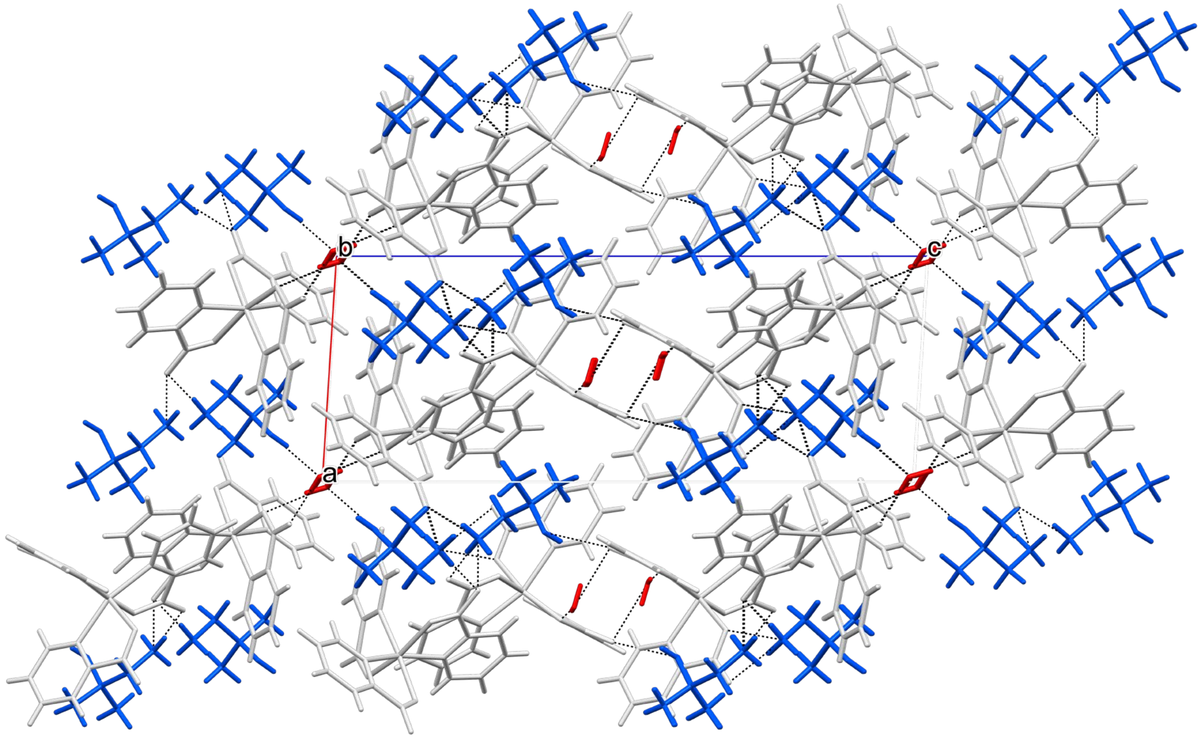

Figure S21. Packing diagram for the structure of (1a2m2pOH$_2$)[Zn(pic)$_3$]·CH_3OH (**11a**). Colour code: grey – complex molecules, blue – 1a2m2pOH$_2^+$ cations, and red – methanol molecules.

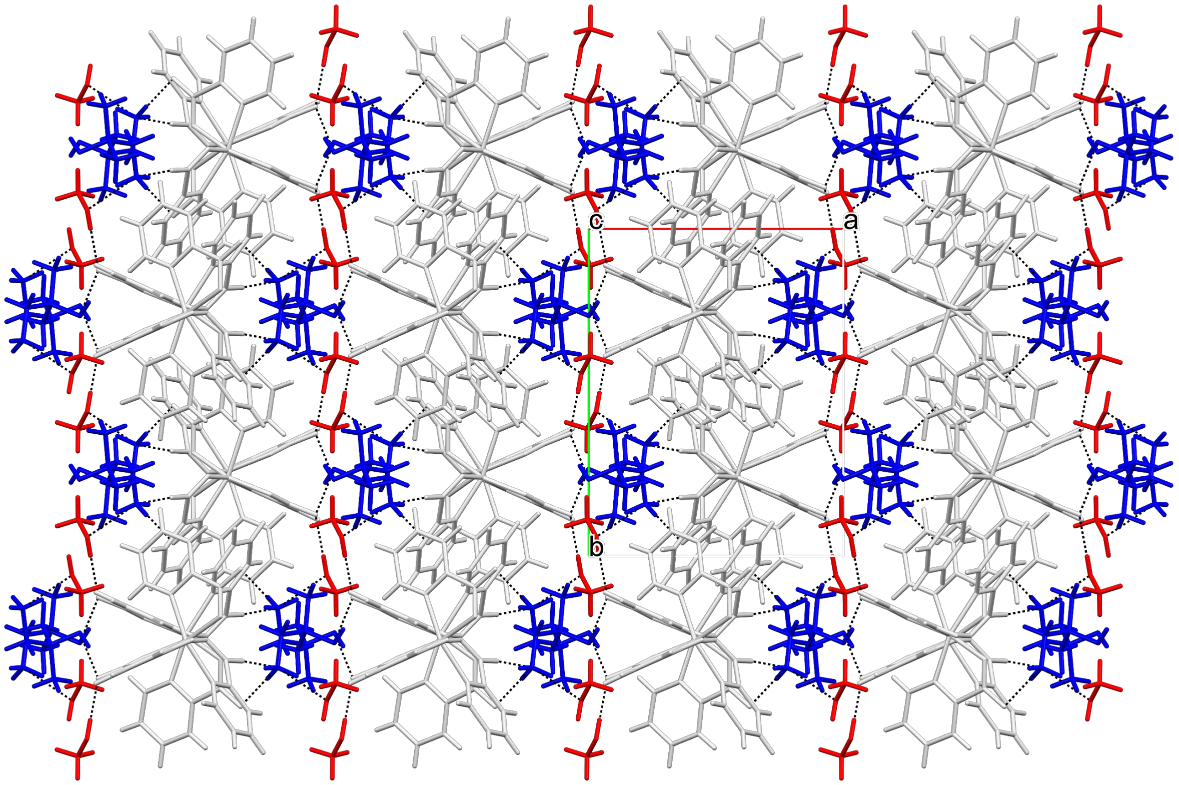

2. Infrared spectra

Figure S22. Infrared spectrum of *trans*-[Zn(pic)$_2$(CH$_3$OH)$_2$] (**1**).

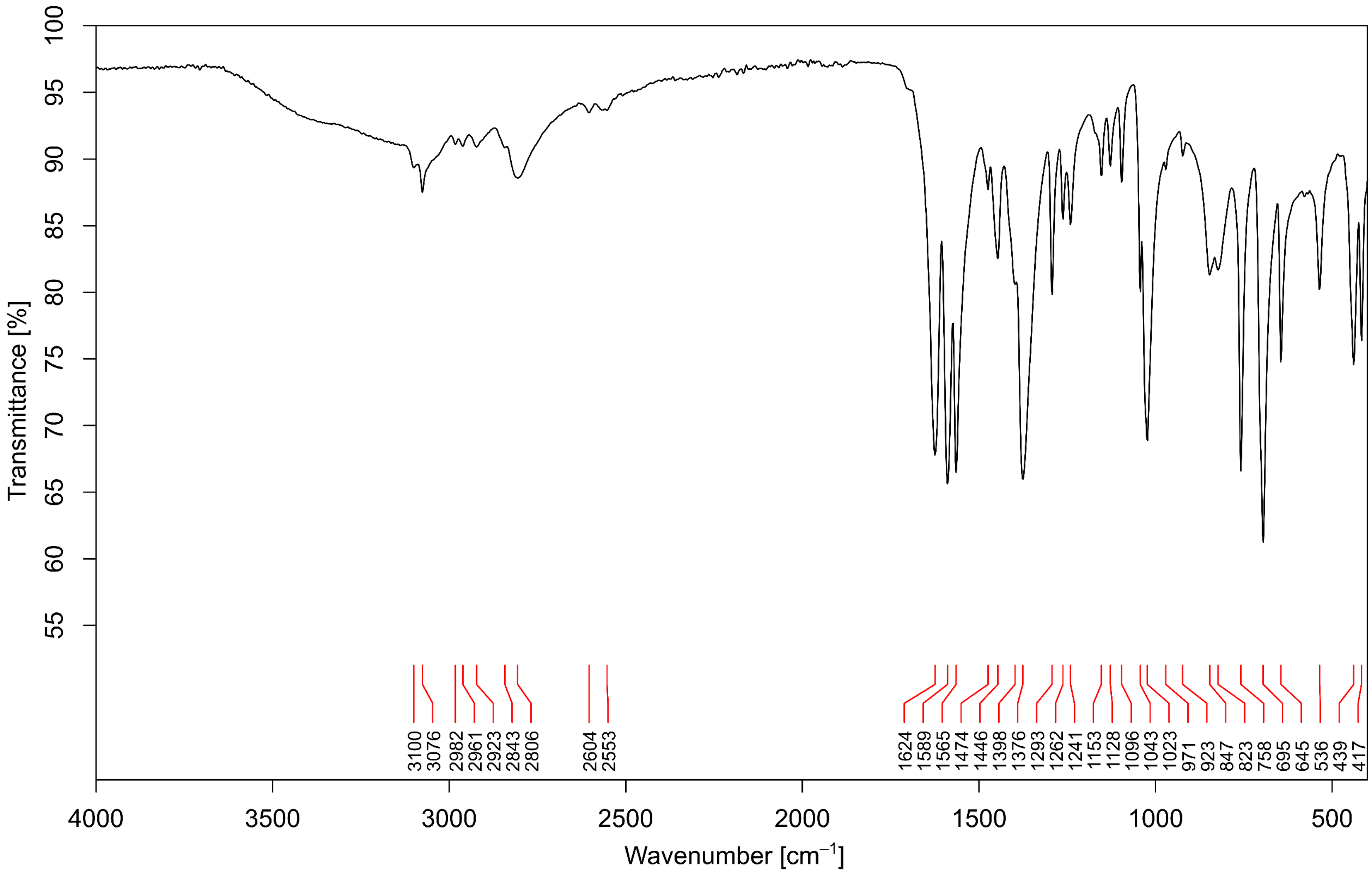

Figure S23. Infrared spectrum of *cis*-[Zn(pic)$_2$(H_2O)$_2$]·0.5CH_3CH_2CN (**2**).

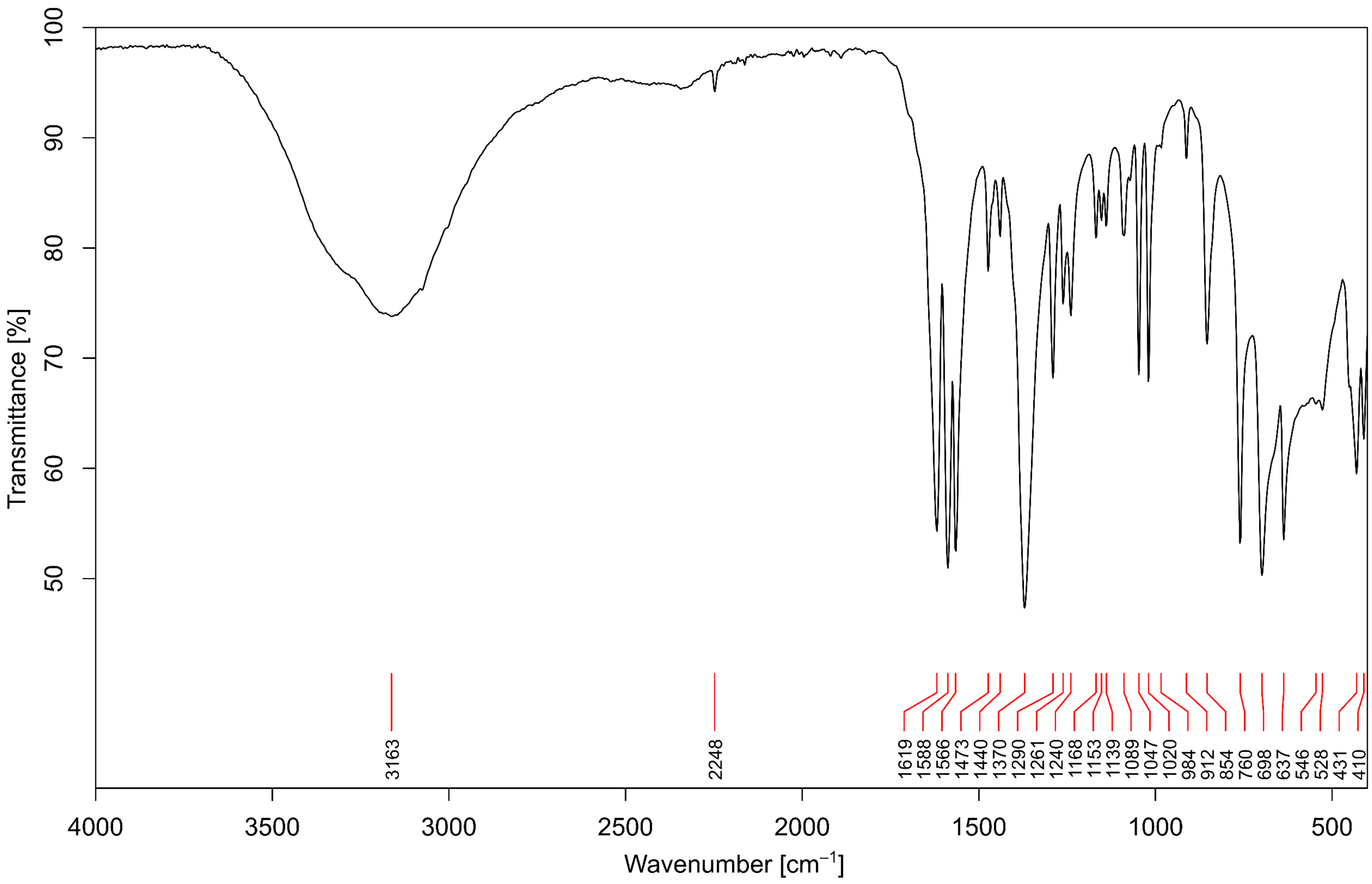

Figure S24. Infrared spectrum of [Zn(pic)$_2$(2aeOH)] (**3**).

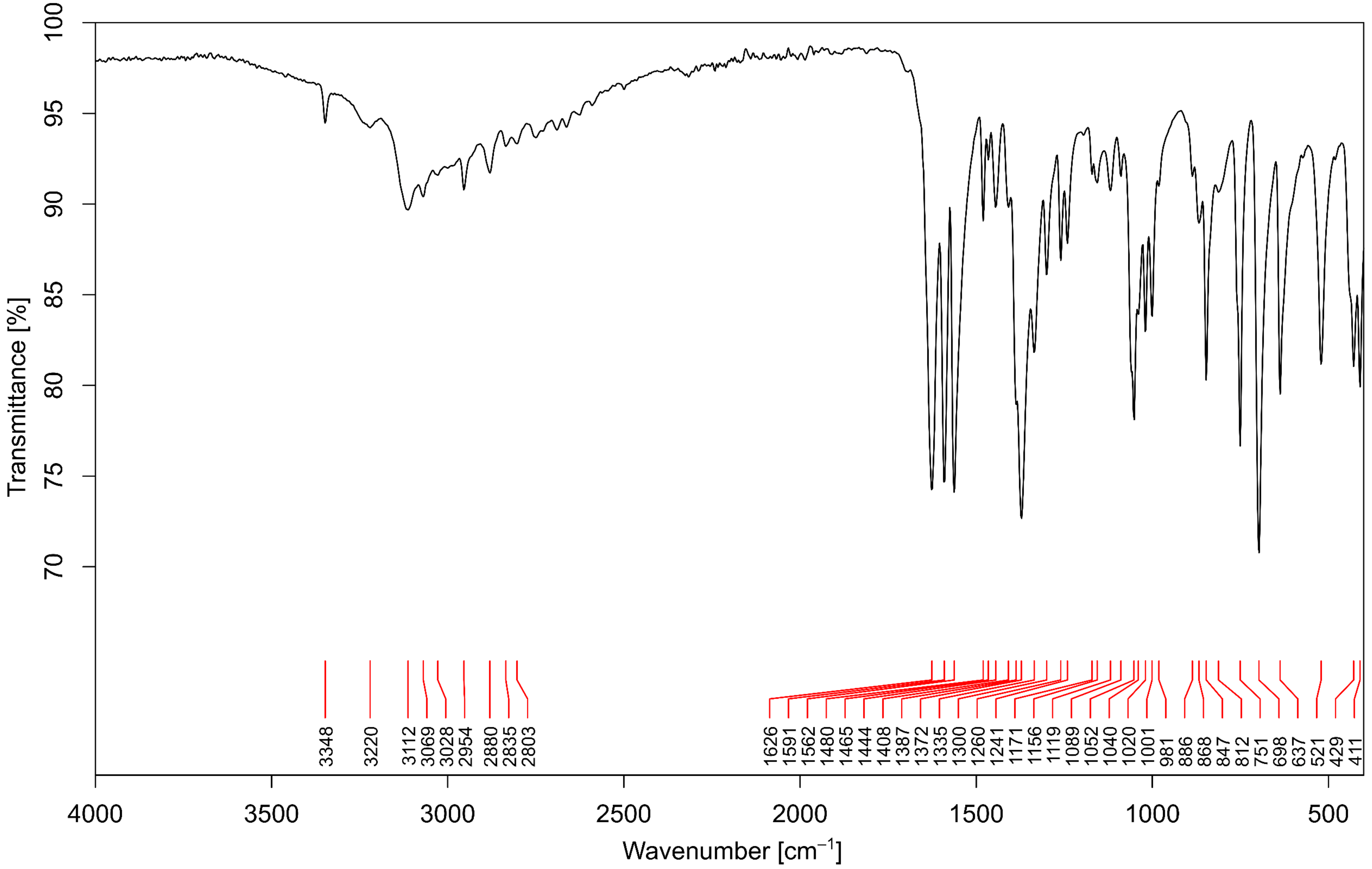

Figure S25. Infrared spectrum of [Zn(pic)$_2$(2maeOH)] (**4**).

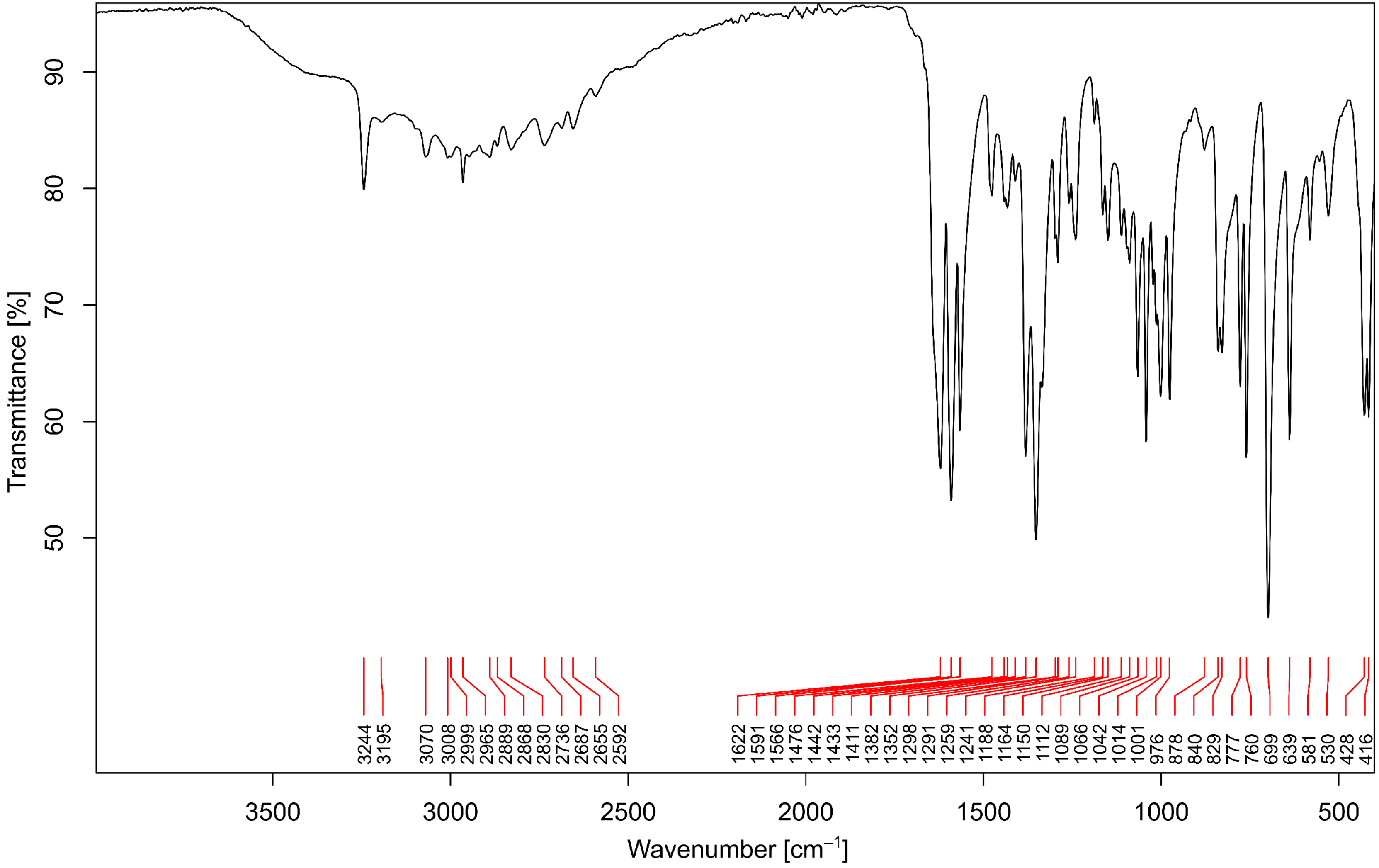

Figure S26. Infrared spectrum of [Zn(pic)$_2$(2eaeOH)] (**5**).

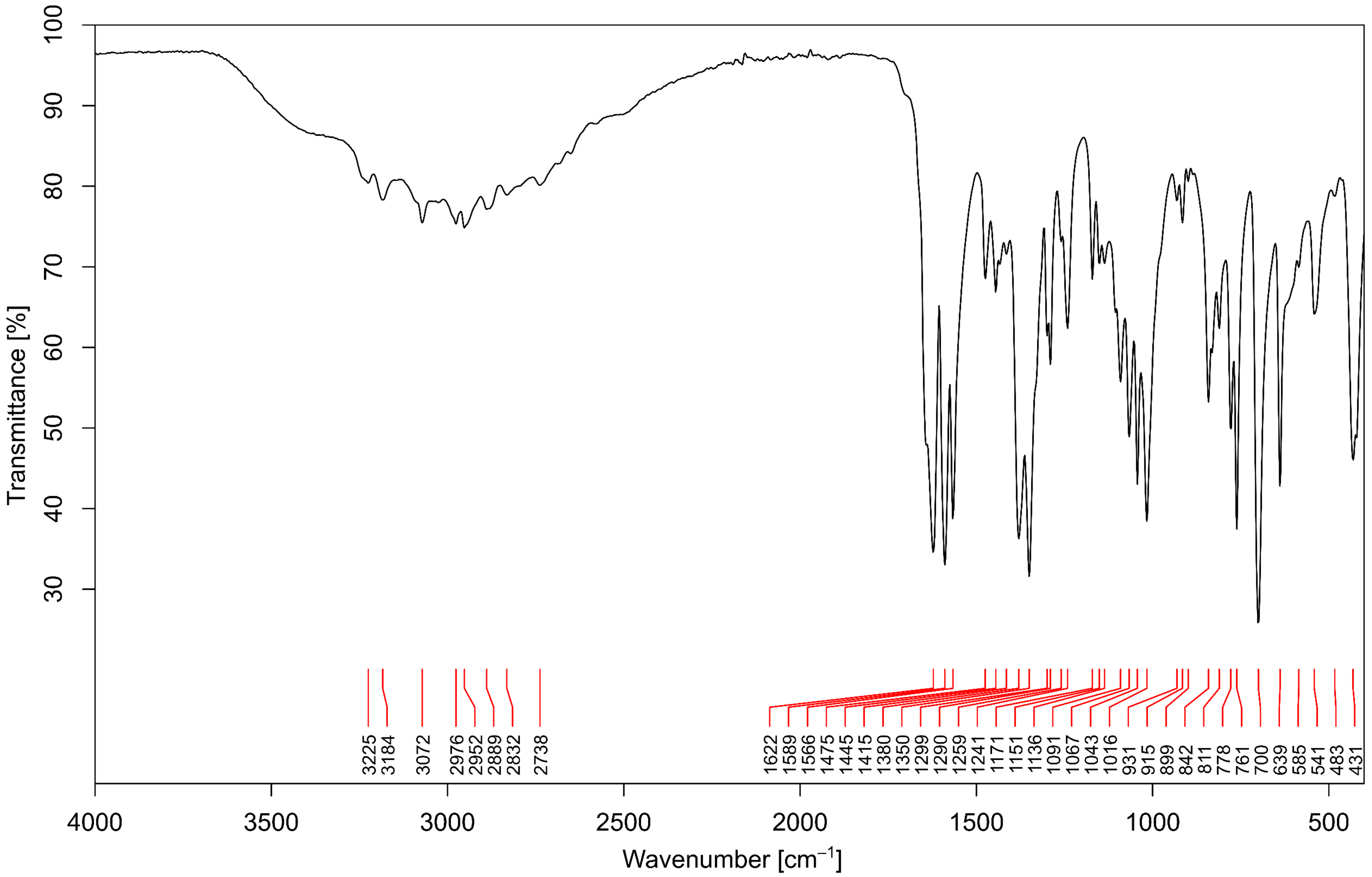

Figure S27. Infrared spectrum of [Zn(pic)$_2$(2dmaeOH)] (**6**).

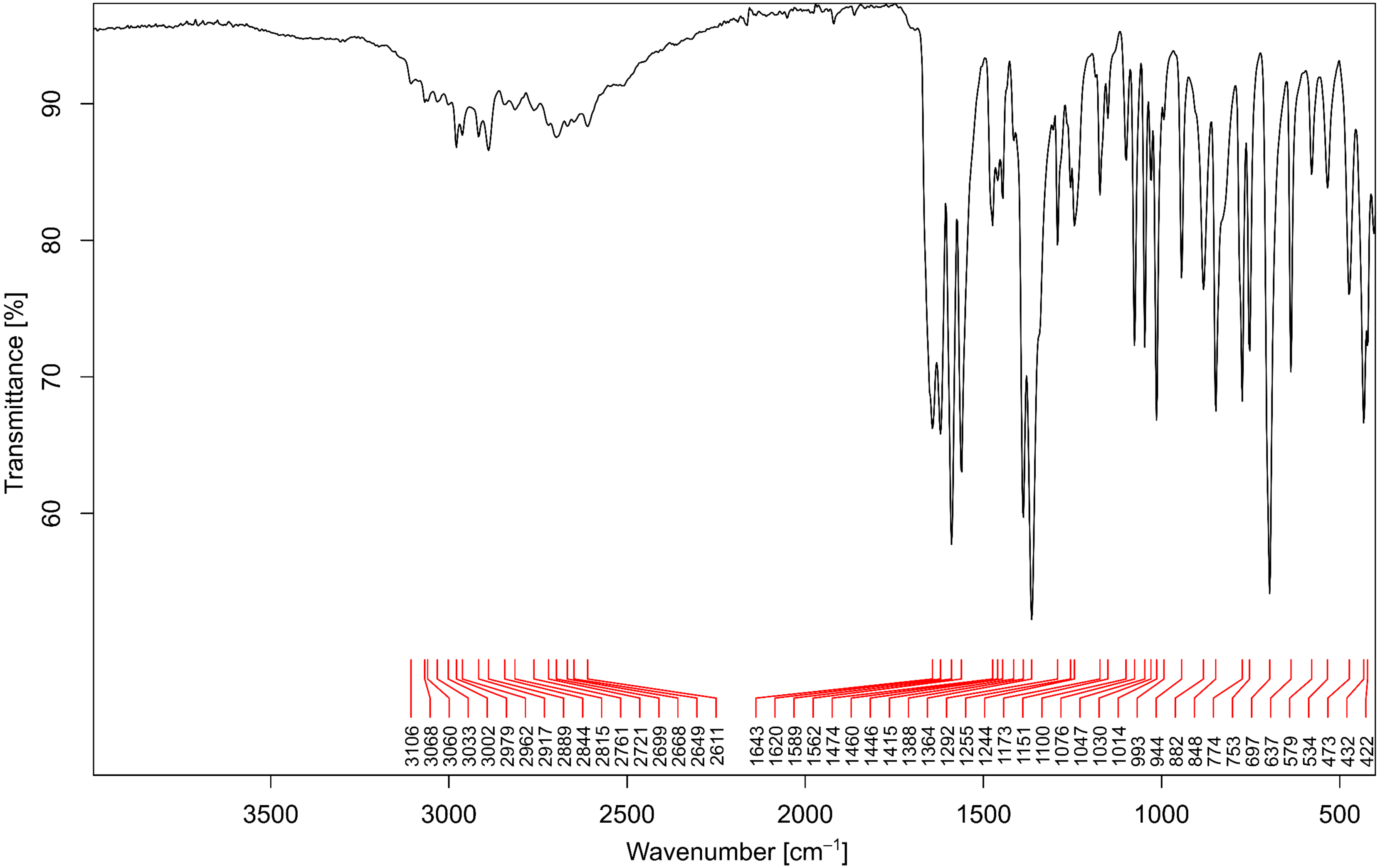

Figure S28. Infrared spectrum of [Zn(pic)$_2$(2a1pOH)]·H_2O (**7**).

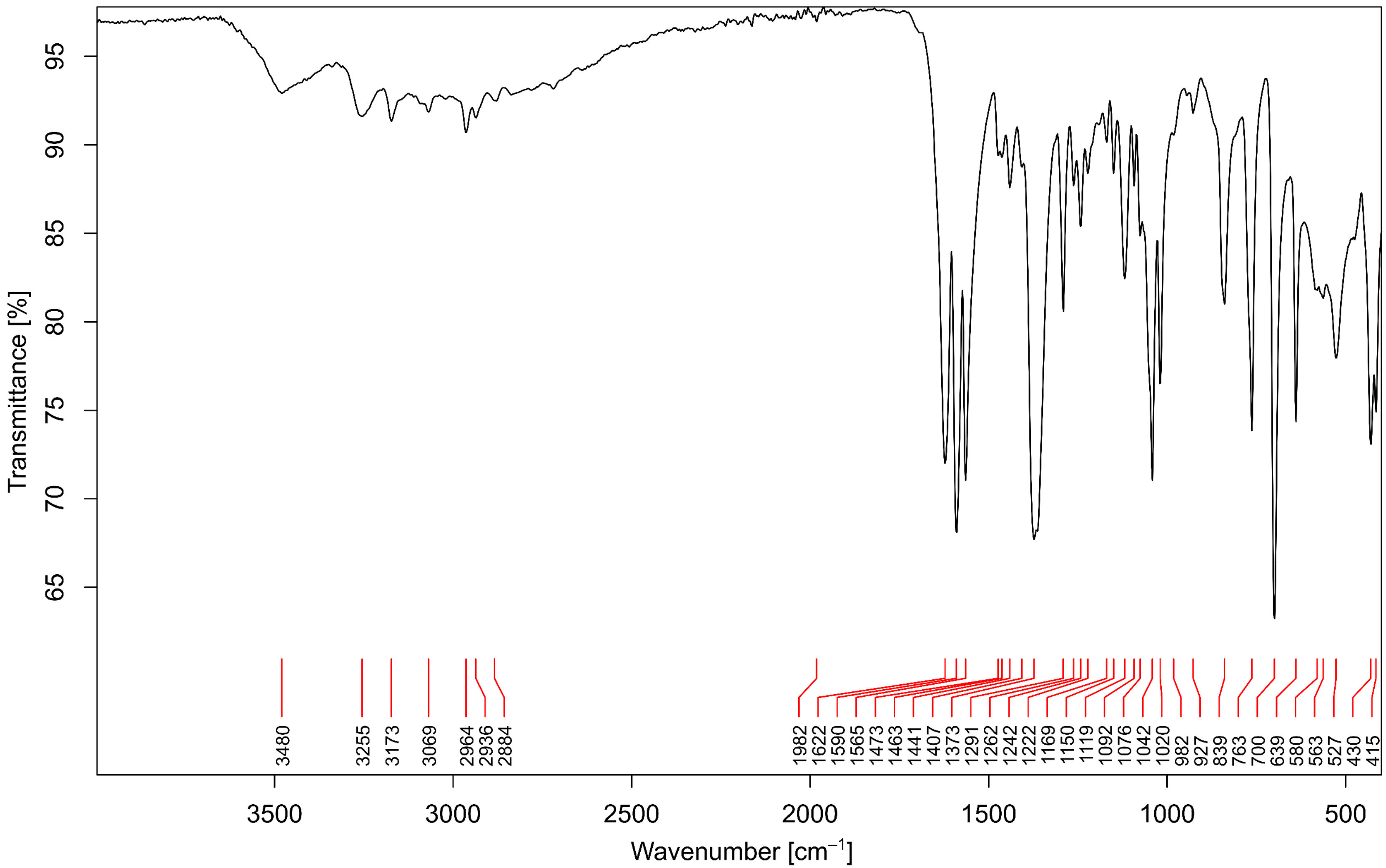

Figure S29. Infrared spectrum of $[Zn(pic)_2(1a2bOH)]\cdot CH_3CN$ (**9**).

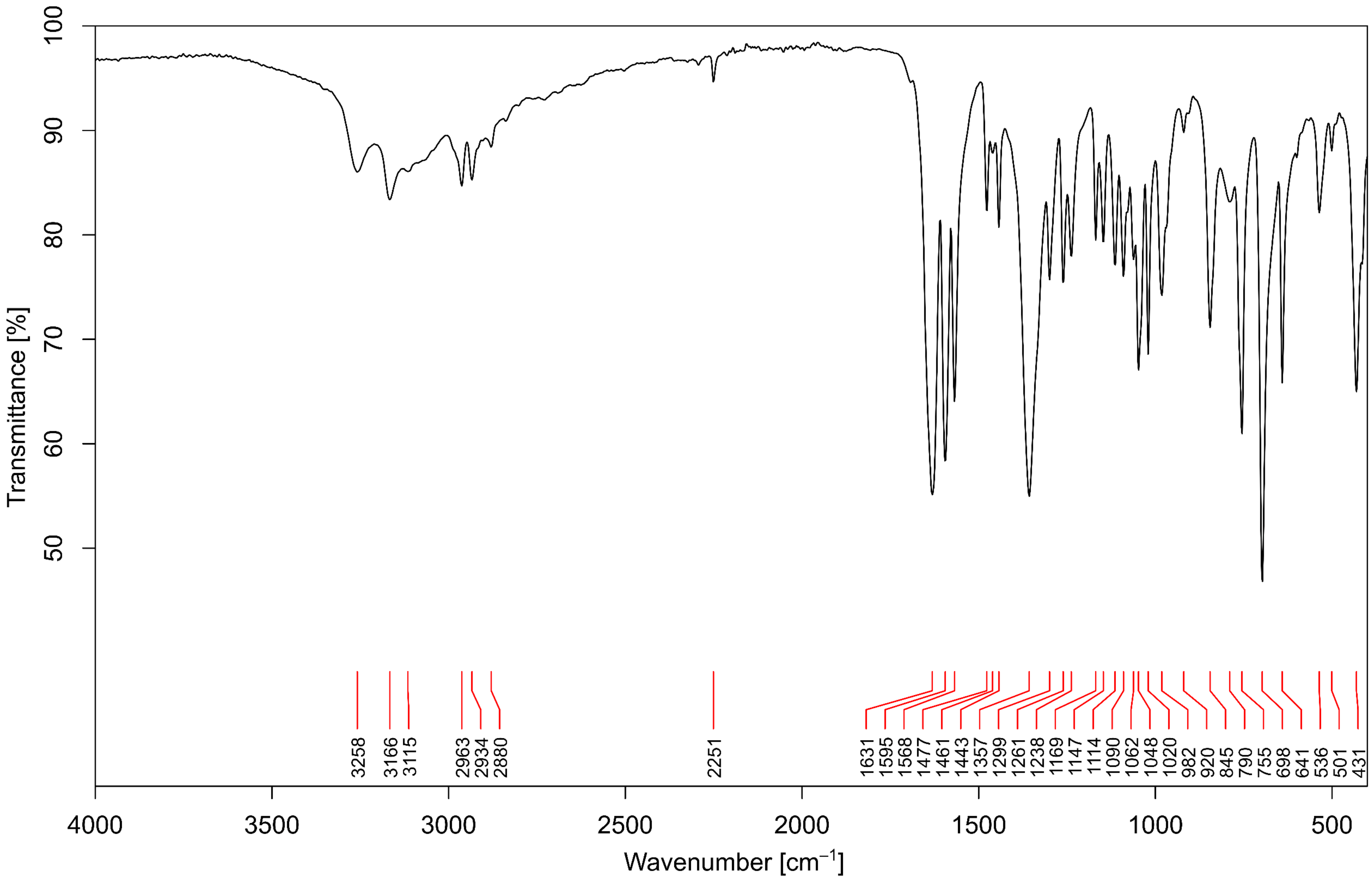

Figure S30. Infrared spectrum of [Zn(pic)$_2$(1a2bOH)]·CH_3CN (**9a**), needle-shaped crystals.

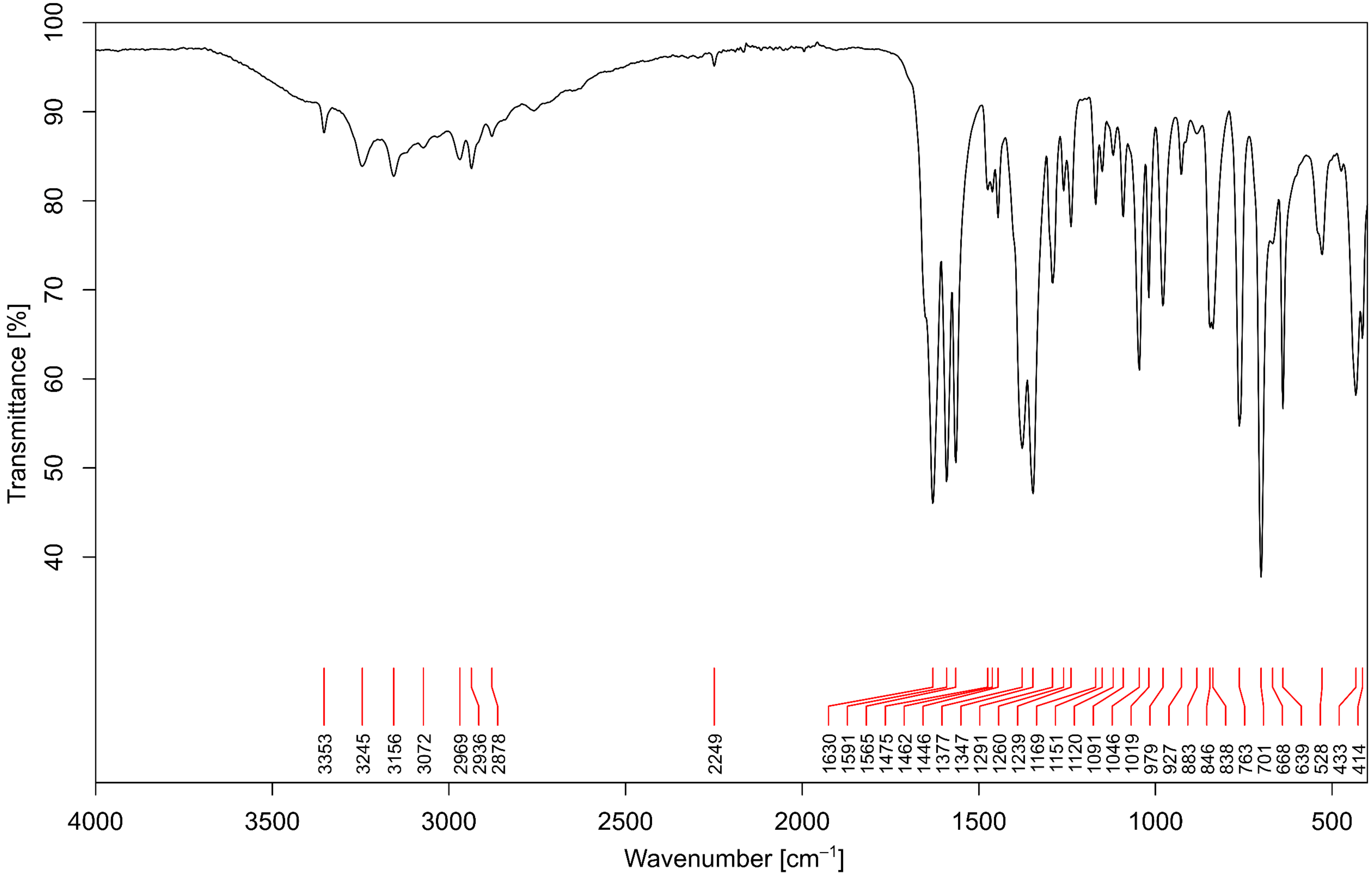

Figure S31. Infrared spectrum of [Zn(pic)$_2$(1a2m2pOH)]·CH_3OH (**10**).

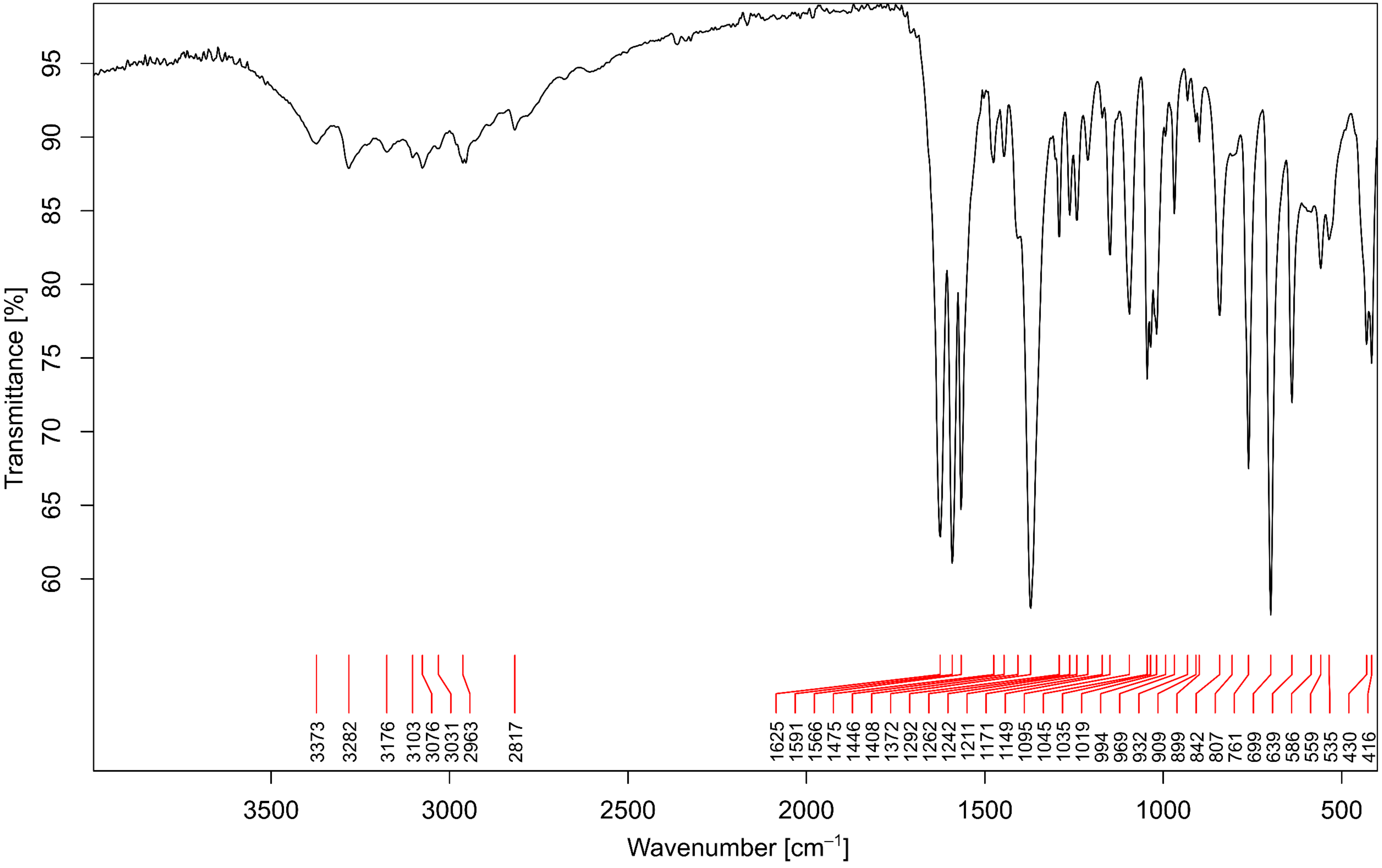

Figure S32. Infrared spectrum of (1a2m2pOHH)[Zn(pic)$_3$]·CH_3OH (**11a**).

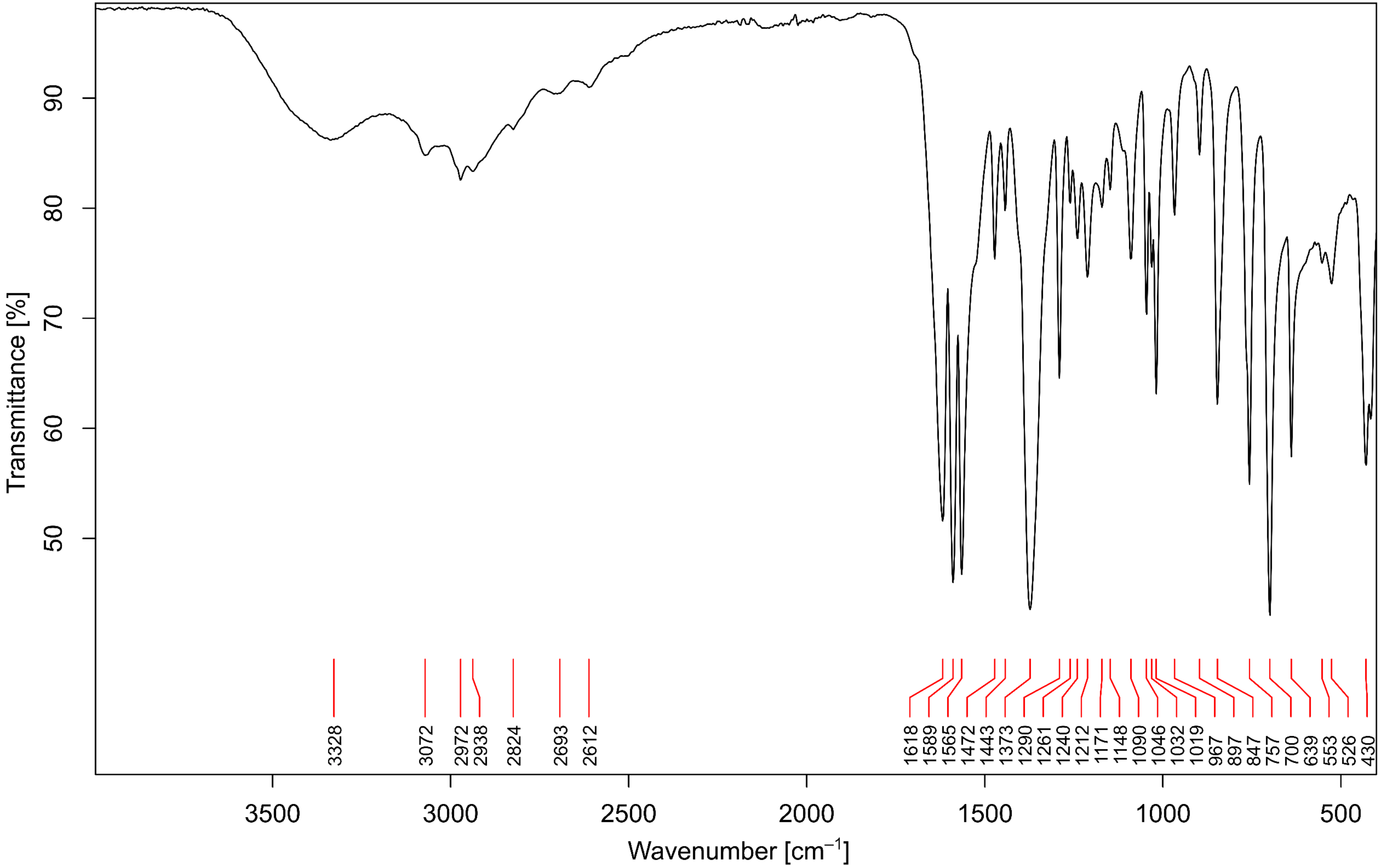

Figure S33. Monitoring the decomposition of *trans*-[Zn(pic)$_2$(CH_3OH)$_2$] (**1**) in the air with IR spectroscopy. The decomposition is slower when the sample is kept in the ATR holder than if it is left in the open air. Colour code: black curve – IR spectrum of freshly prepared crystals of **1**, blue curve – spectrum of sample after 60 minutes exposure, red curve – spectrum of sample after 24 hour exposure. The changes, associated with the losss of methanol molecules are: the disapperance of the 2982, 2961, 2923, 2806, 1128 and 823 cm^{-1} bands and changes in the intensity of the 1043 and 1023 cm^{-1} bands. Furthermore, a broad band, centred at *ca*. 3400 cm^{-1}, appears with time.

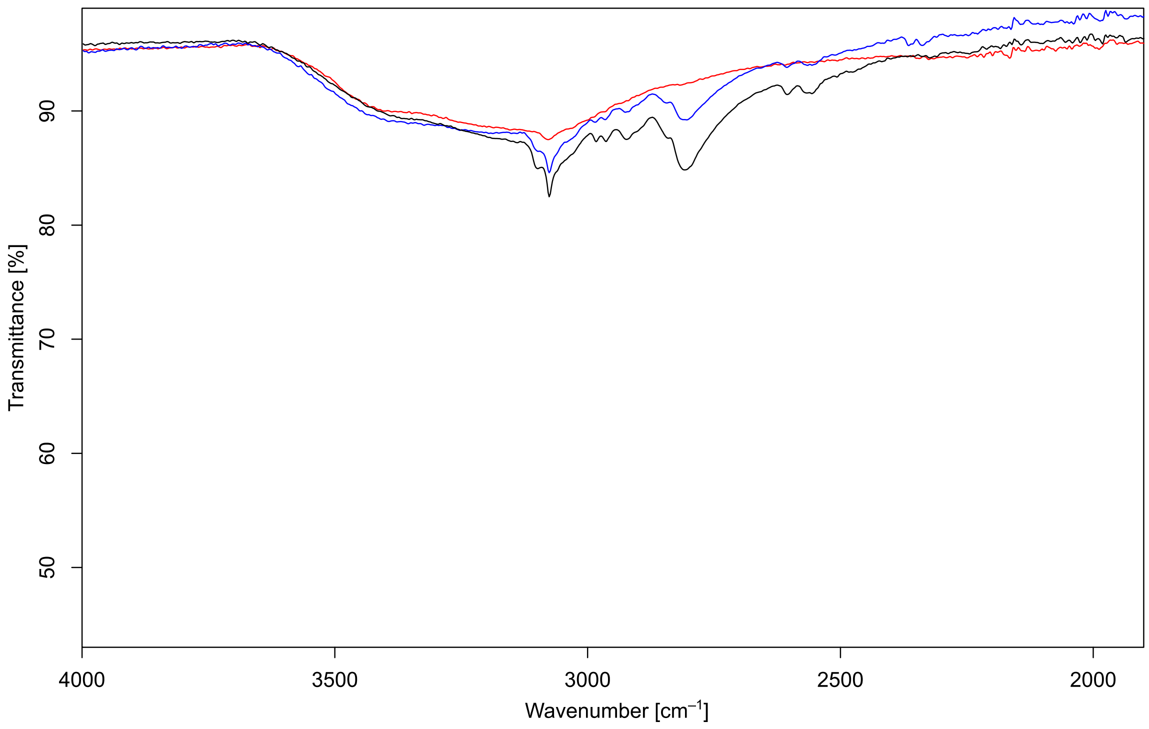

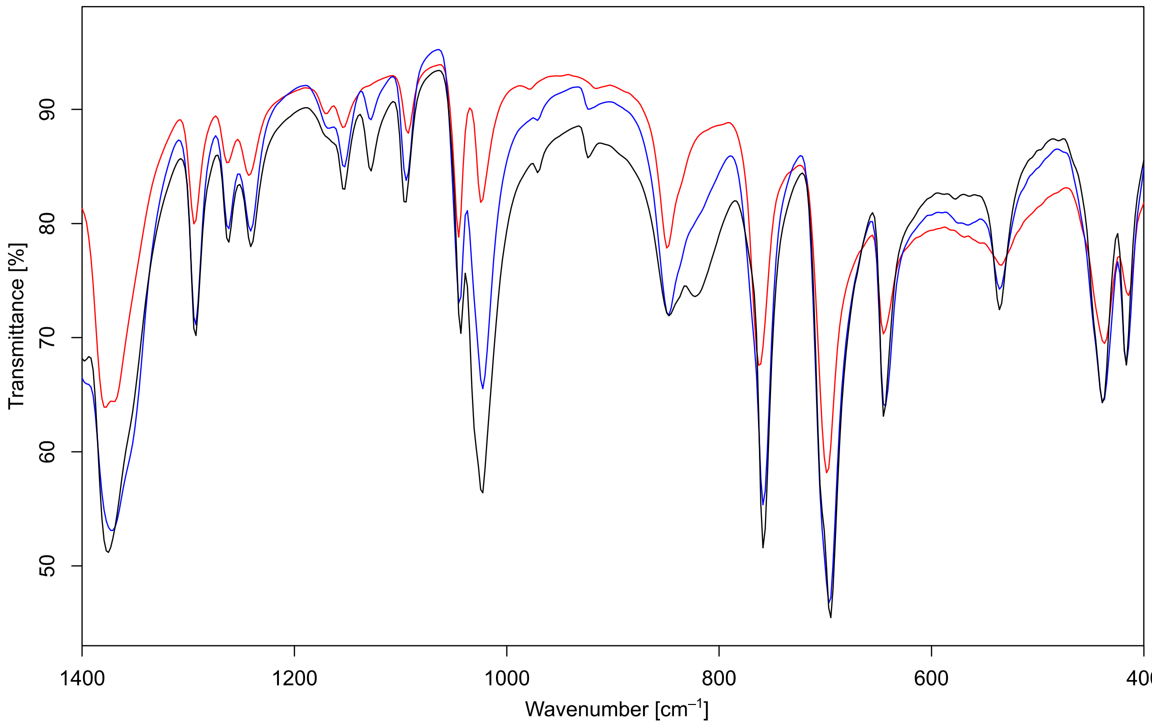

3. ^{1}H NMR spectra

Figure S34. ^{1}H NMR spectrum of DMSO-d_6 solution of *trans*-$[Zn(pic)_2(CH_3OH)_2]$ (**1**).

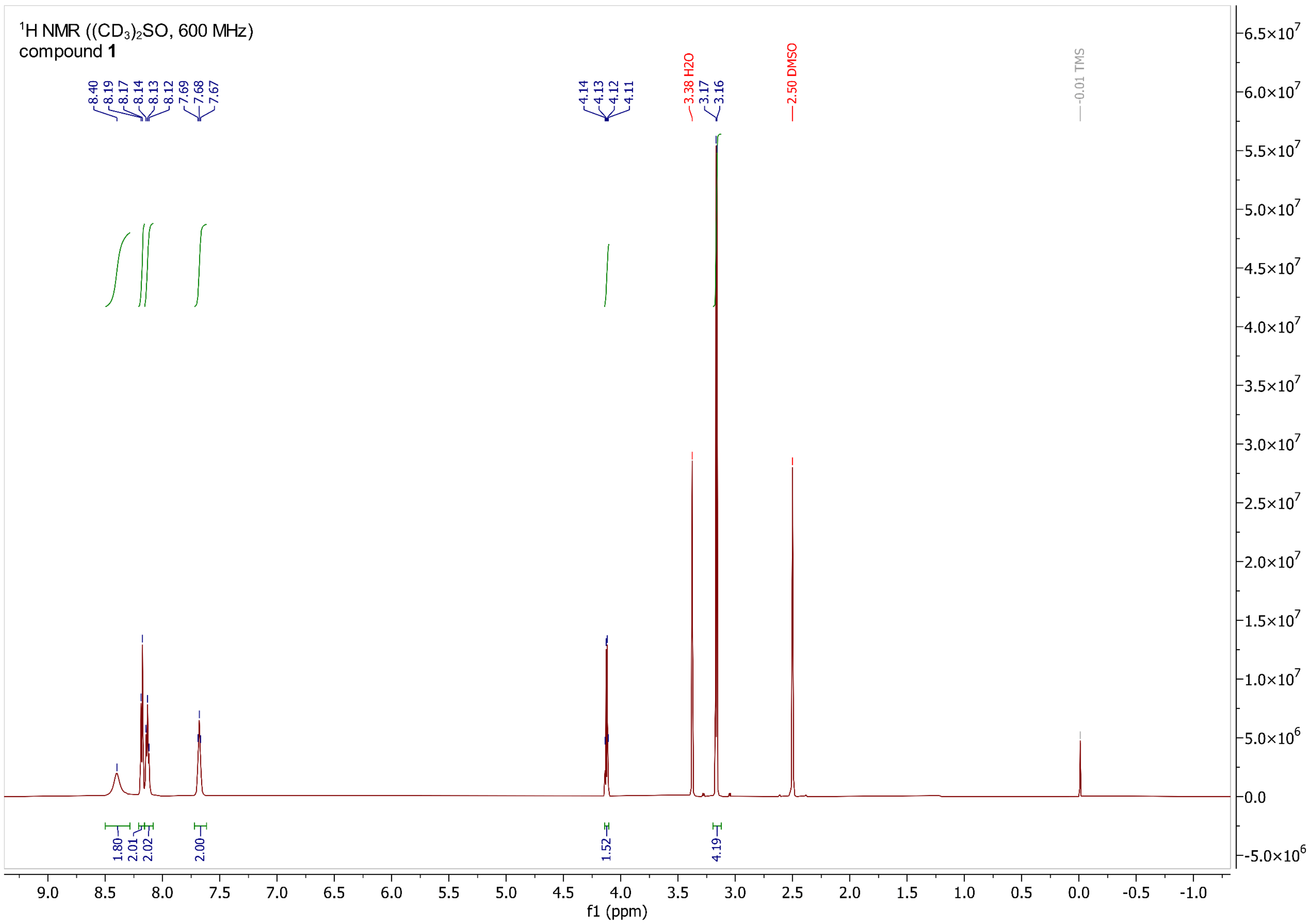

Figure S35. ^{1}H NMR spectrum of DMSO-d_6 solution of *cis*-$[Zn(pic)_2(H_2O)_2]\cdot 1/2CH_3CH_2CN$ (**2**).

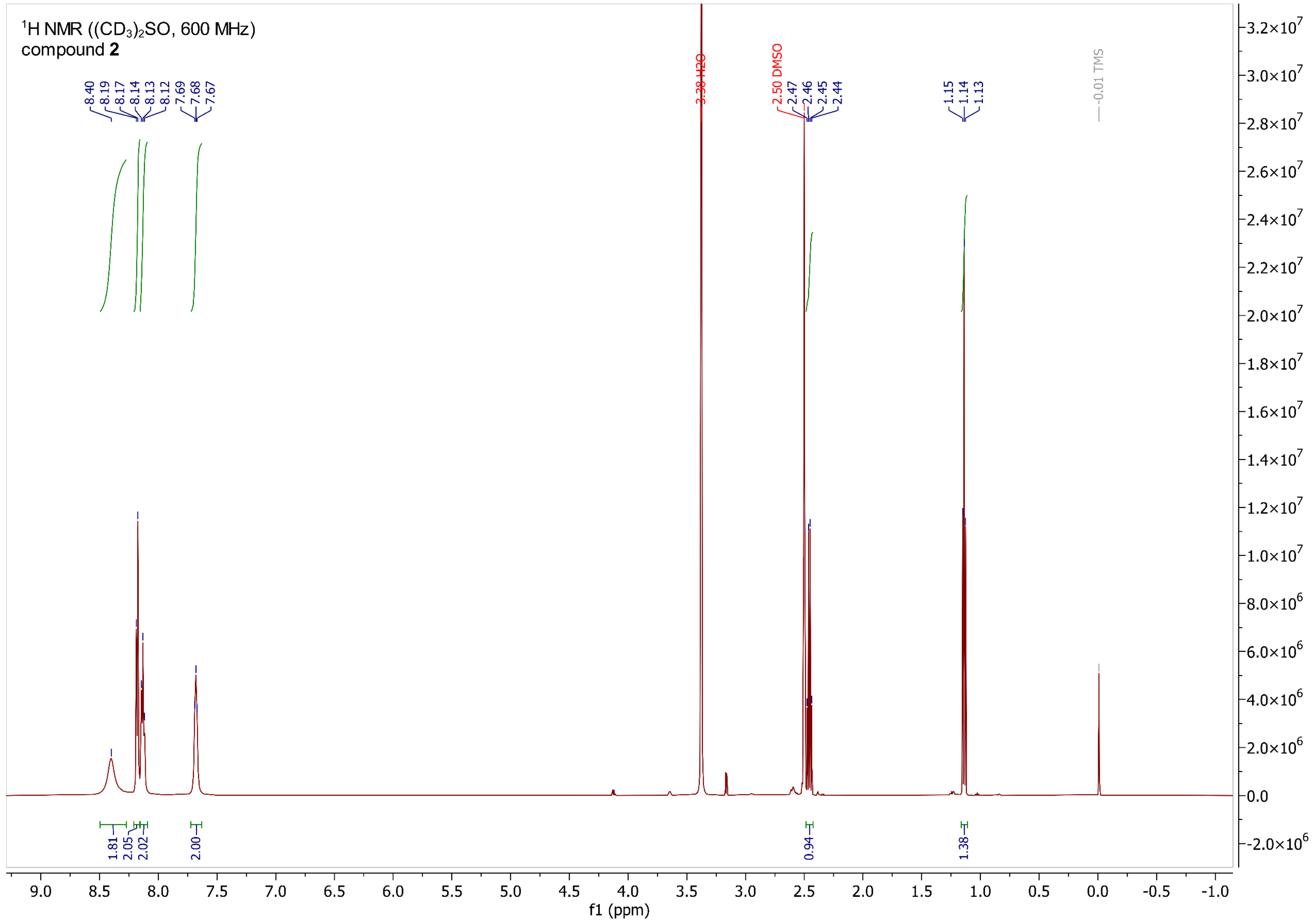

Figure S36. ^{1}H NMR spectrum of DMSO-d_6 solution of [Zn(pic)$_2$(2aeOH)] (**3**).

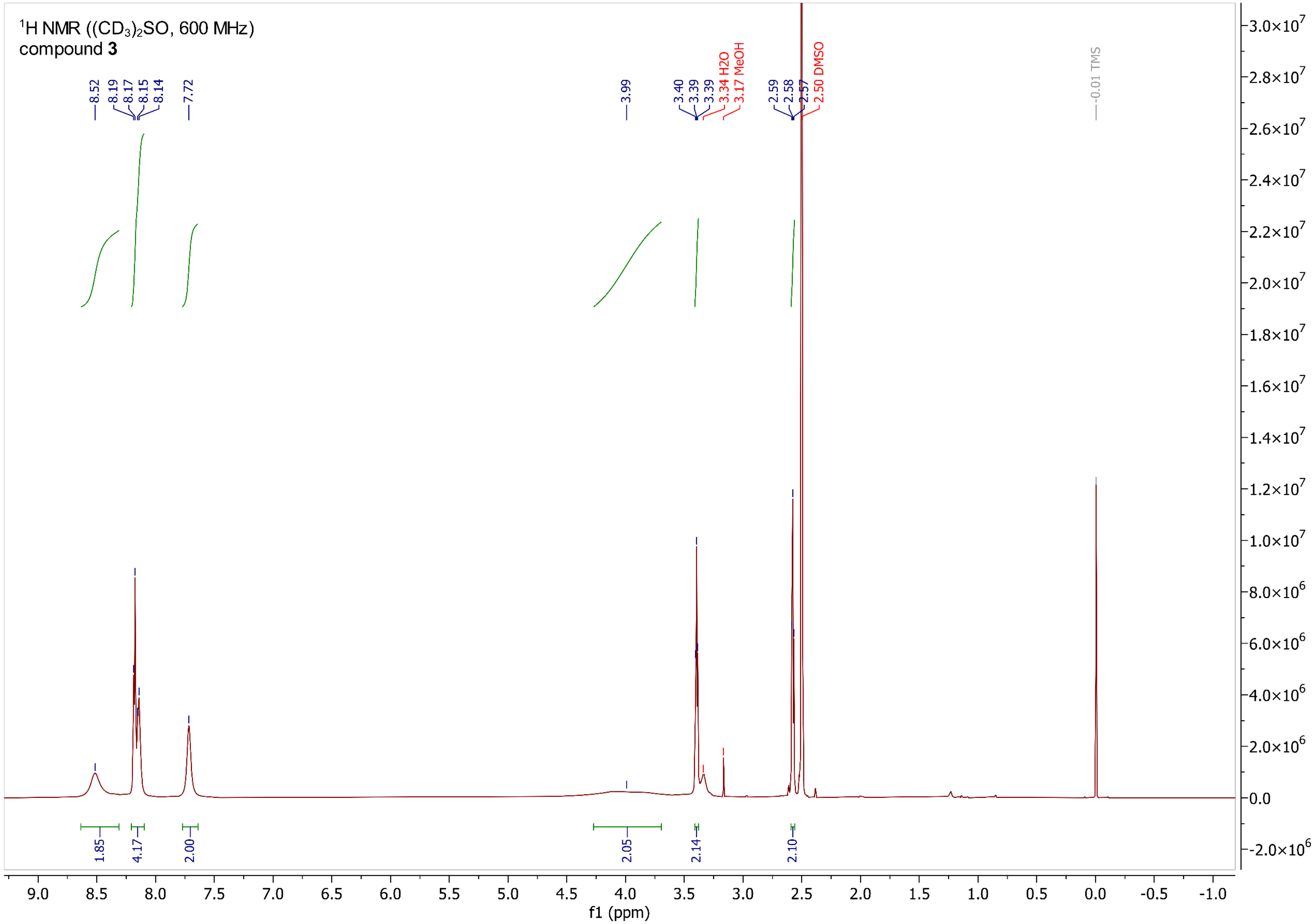

Figure S37. ^{1}H NMR spectrum of DMSO-d_6 solution of $[Zn(pic)_2(2maeOH)]$ (**4**).

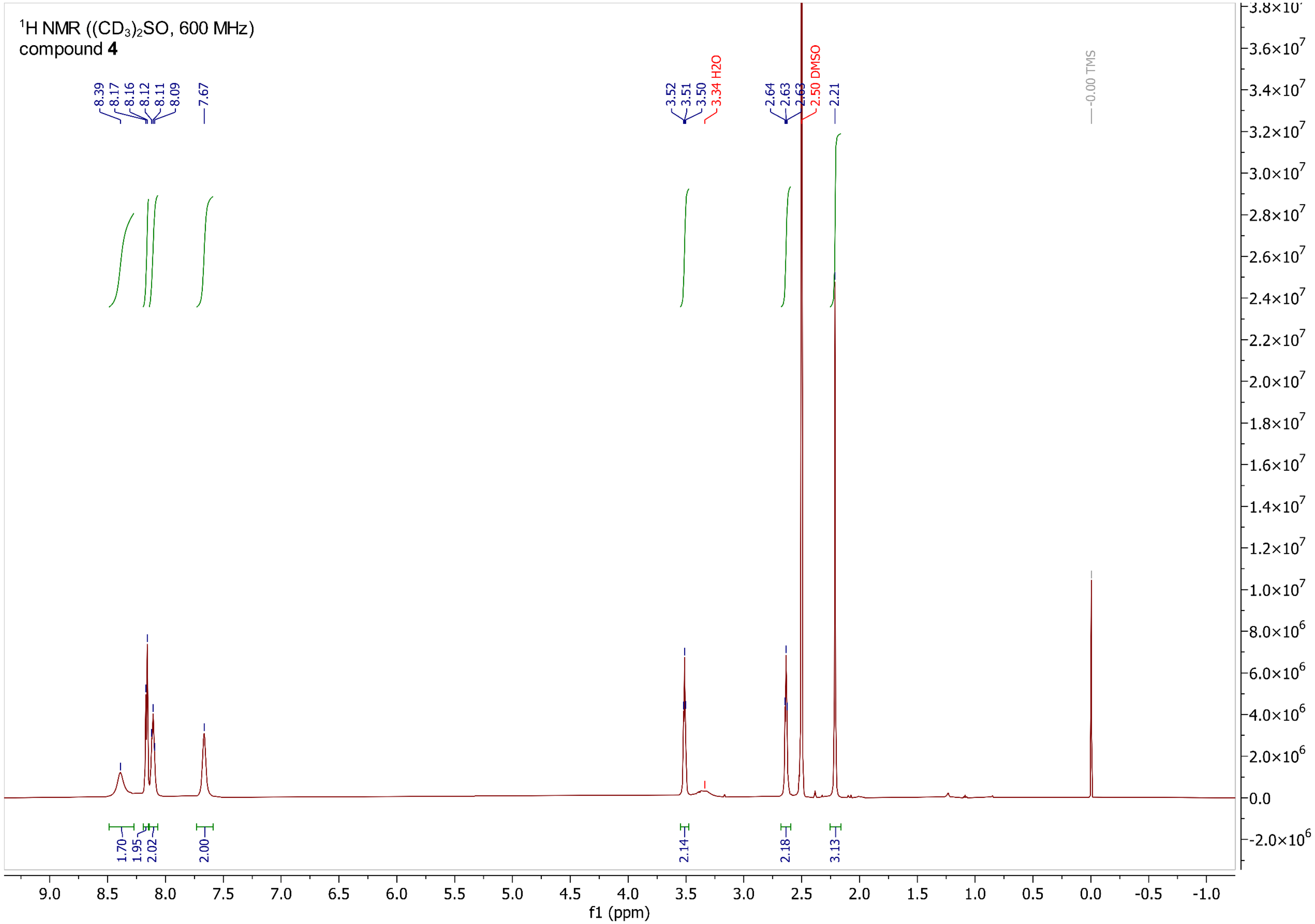

Figure S38. ^{1}H NMR spectrum of DMSO-d_6 solution of $[Zn(pic)_2(2eaeOH)]$ (**5**).

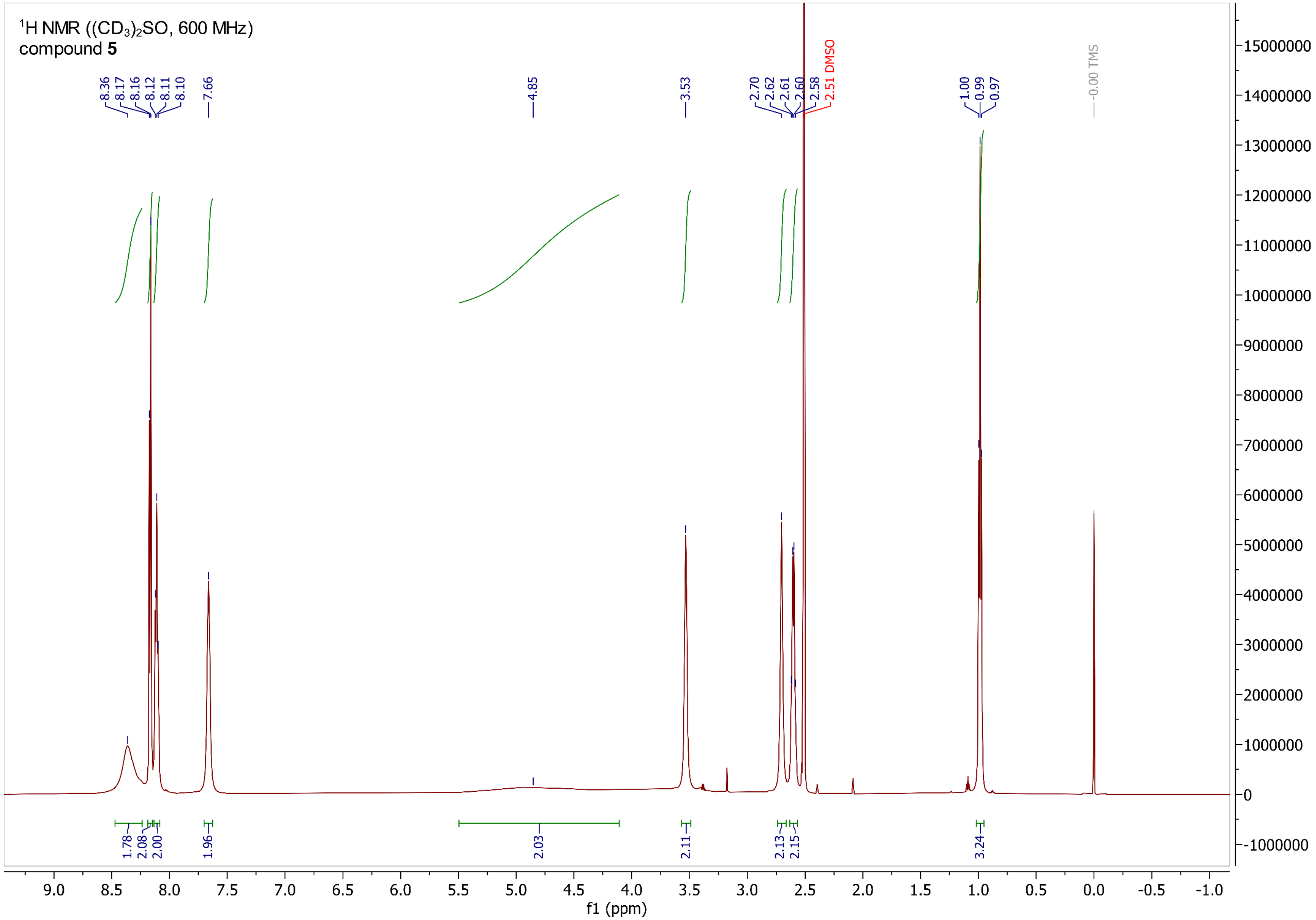

Figure S39. ^{1}H NMR spectrum of DMSO-d_6 solution of $[Zn(pic)_2(2dmaeOH)]$ (**6**).

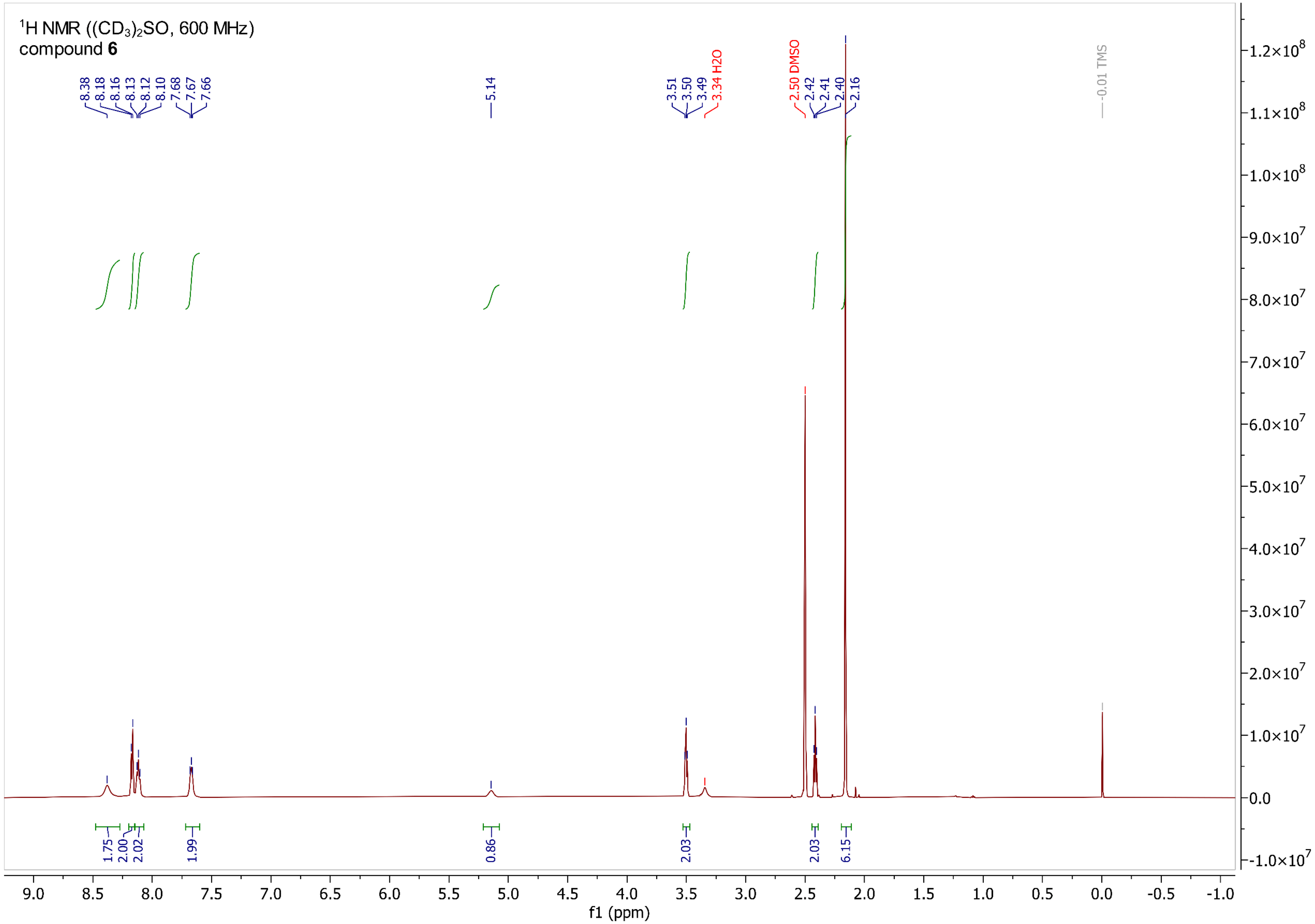

Figure S40. ^{1}H NMR spectrum of DMSO-d_6 solution of $[Zn(pic)_2(2a1pOH)]\cdot H_2O$ (**7**).

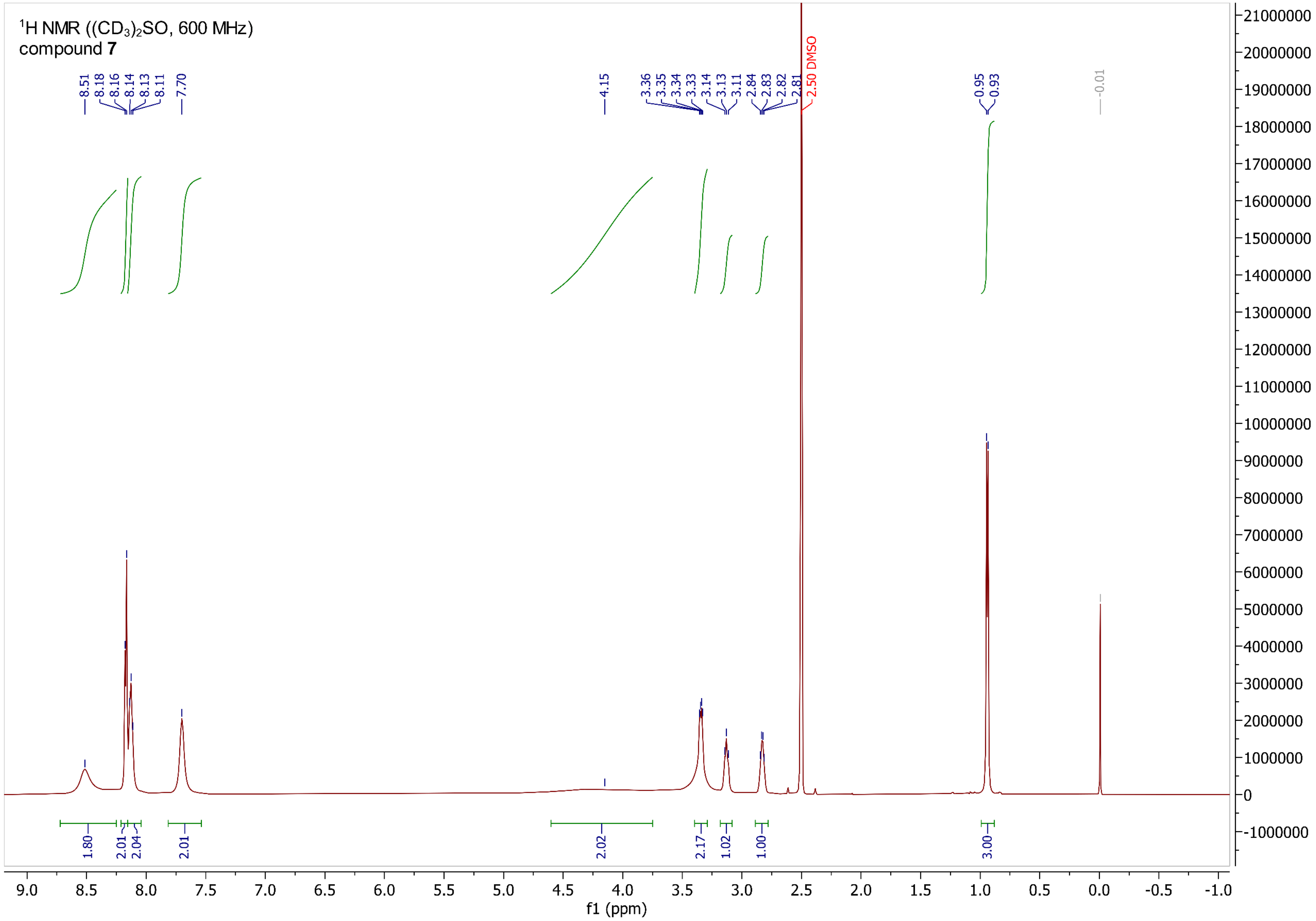

Figure S41. ^{1}H NMR spectrum of DMSO-d_6 solution of [Zn(pic)$_2$(1a2bOH)]·CH_3CN (**9**).

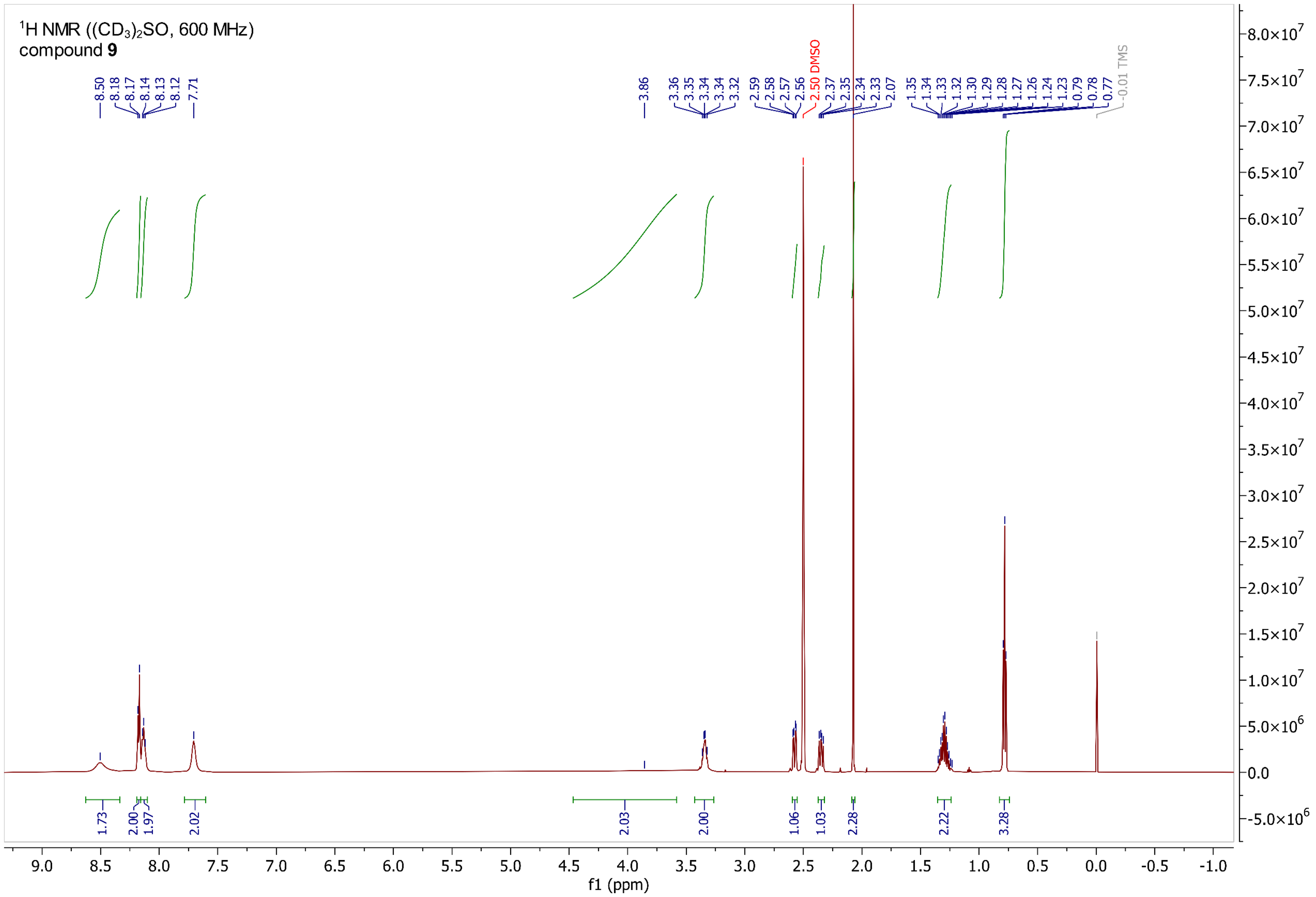

Figure S42. ^{1}H NMR spectrum of DMSO-d_6 solution of $[Zn(pic)_2(1a2m2pOH)]\cdot CH_3OH$ (**10**).

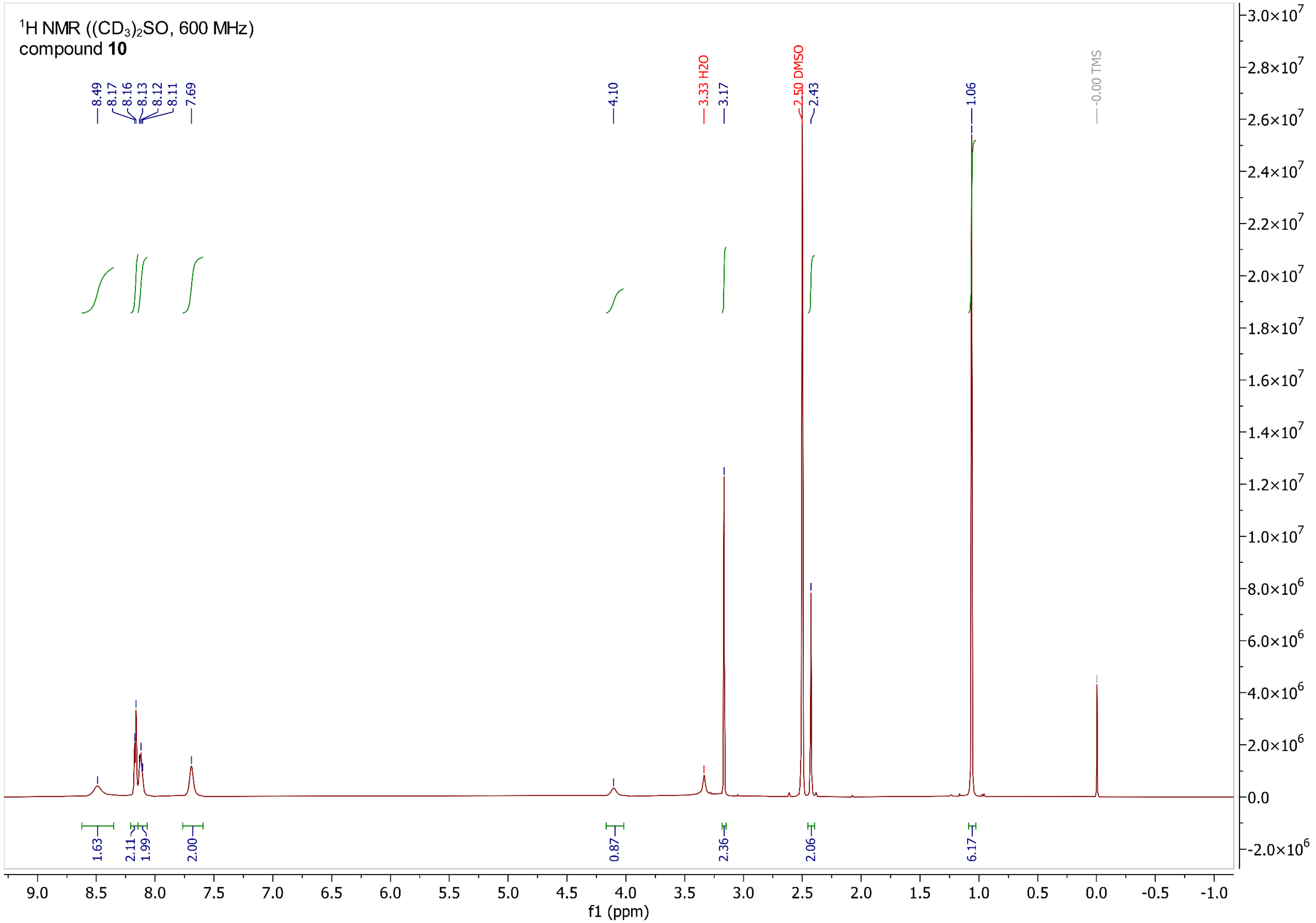

Figure S43. ^{1}H NMR spectrum of DMSO-d_6 solution of $(1a2m2pOH_2)[Zn(pic)_3]\cdot CH_3OH$ (**11a**).

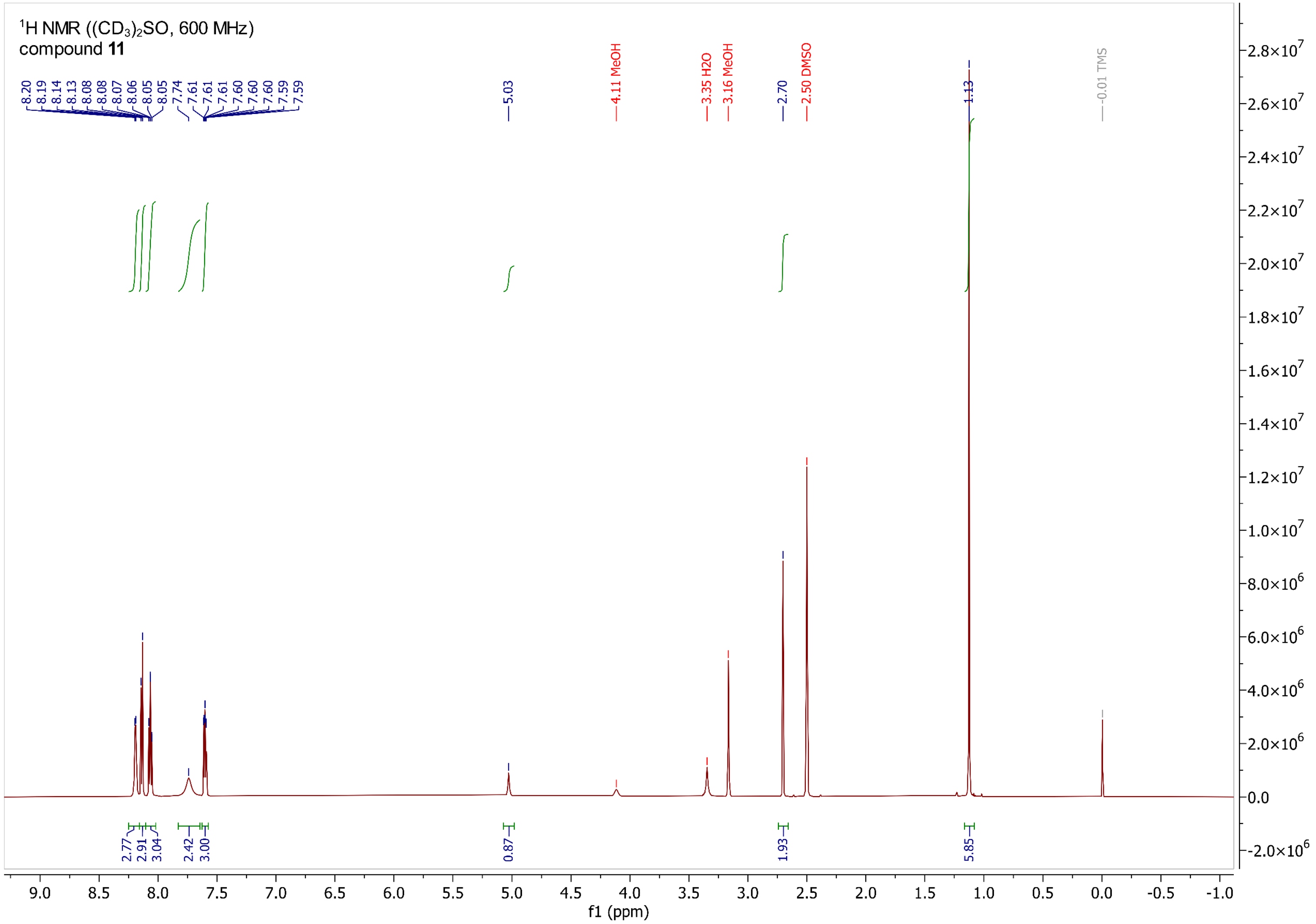

4. DFT calculations

XYZ-coordinates and electronic energies of the optimized species

4fac | E(SCF)=-2901.894765107978 Eh | method=B3PW91-D3BJ/def2-TZVP(SMD)
Zn 1.427946 4.142006 2.827531
O -1.866636 1.837785 2.019071
O 0.464242 5.187769 4.374019
O 2.568592 3.163171 4.517885
H 1.967237 2.826194 5.192334
O -0.037273 2.651045 2.998519
O 0.563346 7.030598 5.624363
N 0.148175 4.630174 1.216339
N 2.569979 5.947952 2.921261
N 2.861424 2.951765 1.748361
H 3.265102 3.568349 1.051417
C -0.873363 3.778342 1.092283
C 2.160222 6.764028 3.896725
C 0.277582 5.635257 0.355998
H 1.123935 6.295609 0.503045
C -0.943342 2.659196 2.111915
C -1.812681 3.911058 0.084442
H -2.626549 3.202800 0.014077
C -0.620013 5.836271 -0.678848
H -0.482040 6.666246 -1.359487
C 0.968284 6.301249 4.707826
C -1.682764 4.957753 -0.814997
H -2.403578 5.086160 -1.613564
C 2.783067 7.974613 4.147867
H 2.421811 8.604367 4.948867
C 3.928762 2.566159 2.682292
H 4.546193 3.447551 2.867111
H 4.567350 1.784932 2.256704
C 3.598106 6.310113 2.158482
H 3.891698 5.629134 1.369415
C 4.272045 7.505074 2.342606
H 5.102765 7.759511 1.697441
C 3.348550 2.087580 3.988382
H 2.717850 1.206661 3.844953
H 4.154993 1.830848 4.680160
C 3.858154 8.350416 3.358766
H 4.364048 9.292496 3.532752
C 2.283426 1.809284 1.034027
H 1.802020 1.129733 1.734612
H 3.053680 1.260916 0.481949
H 1.531327 2.163951 0.330019

4*fac*_b | E(SCF)=-2901.893751167977 Eh | method=B3PW91-D3BJ/def2-TZVP(SMD)
Zn 1.490066 4.221063 2.887806
O -1.726687 1.845929 1.916279
O 0.449427 5.199613 4.419295
O 2.712004 3.321778 4.585411
H 2.172398 3.018111 5.324641
O 0.043214 2.677553 2.987499
O 0.415980 7.055775 5.653593
N 0.248704 4.703602 1.250550
N 2.589907 6.031113 3.058269
N 2.800570 2.866032 1.864322
C -0.732654 3.817529 1.060764
C 2.106683 6.835420 4.010467
C 0.392201 5.722161 0.408354
H 1.205996 6.408569 0.609674
C -0.824010 2.682653 2.062830
C -1.616618 3.929644 0.001299
H -2.399715 3.194656 -0.122750
C -0.450384 5.903105 -0.675131
H -0.302082 6.744153 -1.339807
C 0.892099 6.335901 4.764725
C -1.471523 4.989268 -0.880454
H -2.148327 5.100186 -1.719157
C 2.683905 8.061257 4.294323
H 2.262610 8.682030 5.072597
C 3.969490 2.603549 2.708092
H 4.585351 3.505932 2.731097
H 4.579648 1.786797 2.306776
C 3.654550 6.415814 2.359321
H 4.010871 5.736230 1.597422
C 4.288511 7.626066 2.582111
H 5.152053 7.898439 1.989475
C 3.516394 2.243538 4.099232
H 2.928400 1.320777 4.081254
H 4.381869 2.095412 4.750284
C 3.793507 8.463090 3.568103
H 4.264749 9.417553 3.769769
H 2.221678 2.028675 1.869878
C 3.149650 3.148252 0.472268
H 3.738916 4.061717 0.409612
H 3.732639 2.331246 0.033977
H 2.239966 3.281797 -0.111939

4tbp | E(SCF)=-2901.895225052040 Eh | method=B3PW91-D3BJ/def2-TZVP(SMD)
Zn 0.764437 4.676152 2.571031
O -2.225795 2.537778 4.363554
O 1.603544 6.047440 1.222061
O 2.199406 2.794850 4.836901
H 1.247881 2.903377 4.652050
O -0.276453 3.564786 4.018130
O 2.883522 7.873064 1.161963
N -1.146186 4.715356 1.799414
N 1.552699 6.046466 3.879023
N 1.877781 3.117446 1.781960
H 2.231212 3.575664 0.945657
C -2.020044 3.971082 2.486059
C 2.231041 7.029361 3.276945
C -1.537100 5.345684 0.694844
H -0.785050 5.930059 0.180018
C -1.485115 3.289708 3.725834
C -3.334904 3.835226 2.081090
H -4.013475 3.225140 2.660856
C -2.837367 5.260495 0.227626
H -3.120984 5.788589 -0.673235
C 2.253102 6.994968 1.762951
C -3.749566 4.492828 0.932938
H -4.774438 4.405933 0.592956
C 2.866485 8.021144 4.001352
H 3.408868 8.798317 3.481347
C 3.071220 2.687182 2.539619
H 3.669984 3.577763 2.743215
H 3.667861 2.012457 1.913928
C 1.469091 6.014389 5.206912
H 0.900979 5.200084 5.636739
C 2.074389 6.974198 5.998801
H 1.984773 6.917207 7.075588
C 2.773649 1.970028 3.838608
H 2.148988 1.088426 3.660882
H 3.728046 1.611576 4.231711
C 2.787600 7.991106 5.385128
H 3.275806 8.755414 5.977617
C 1.035638 1.996509 1.340694
H 0.485238 1.580149 2.182564
H 1.638897 1.205906 0.884039
H 0.317210 2.360624 0.606726

4mer | E(SCF)=-2901.896500746398 Eh | method=B3PW91-D3BJ/def2-TZVP(SMD)
Zn 1.197210 4.209983 2.771667
O -2.207281 2.775257 4.553157
O 2.352848 5.498054 1.570224
O 2.945494 3.303446 3.903978
H 2.746480 3.220496 4.844157
O -0.064809 3.174729 4.087365
O 3.206154 7.555117 1.468686
N -0.709636 4.682027 1.982553
N 1.381545 5.918329 4.011779
N 1.723329 2.596873 1.485961
H 1.783521 3.031412 0.570877
C -1.709113 4.149421 2.689760
C 2.033585 6.926252 3.428102
C -0.979047 5.445751 0.928795
H -0.128393 5.852494 0.394952
C -1.309805 3.294196 3.874983
C -3.034386 4.368234 2.355647
H -3.818236 3.920734 2.950882
C -2.277978 5.712574 0.529682
H -2.459923 6.339148 -0.333743
C 2.581676 6.652644 2.043347
C -3.321771 5.163203 1.256787
H -4.349831 5.351797 0.971678
C 2.193380 8.150832 4.053467
H 2.726495 8.944965 3.549431
C 3.065421 2.127141 1.856569
H 3.788634 2.859502 1.492734
H 3.293332 1.162568 1.390688
C 0.865279 6.081321 5.225851
H 0.344109 5.230490 5.647428
C 0.982295 7.274842 5.918242
H 0.549195 7.370771 6.905400
C 3.197369 2.006383 3.353443
H 2.483466 1.285461 3.760141
H 4.208248 1.679008 3.610111
C 1.657666 8.326471 5.319931
H 1.765545 9.274224 5.833671
C 0.731059 1.521716 1.417786
H 0.614011 1.056751 2.395384
H 1.027554 0.754144 0.695505
H -0.231821 1.935170 1.119039

4*mer*_b | E(SCF)=-2901.896777175667 Eh | method=B3PW91-D3BJ/def2-TZVP(SMD)
Zn 1.199830 4.268344 2.704265
O -2.228976 2.773568 4.401515
O 2.388368 5.570365 1.577693
O 2.986792 3.379578 3.812123
H 2.837827 3.309284 4.762392
O -0.078960 3.176615 3.972541
O 3.322891 7.593617 1.586274
N -0.696292 4.768882 1.919393
N 1.369120 5.929009 4.011274
N 1.671438 2.592088 1.486954
C -1.704183 4.206937 2.590954
C 2.067478 6.936475 3.483330
C -0.951990 5.572152 0.891675
H -0.094427 6.000639 0.386850
C -1.321137 3.310572 3.751615
C -3.024936 4.435196 2.244904
H -3.816495 3.963917 2.810812
C -2.245370 5.850513 0.483240
H -2.416296 6.509091 -0.358325
C 2.645649 6.695709 2.104959
C -3.298138 5.270292 1.172596
H -4.322386 5.466394 0.878953
C 2.246166 8.132408 4.156965
H 2.817952 8.926738 3.697500
C 3.042455 2.163864 1.781516
H 3.724926 2.901927 1.353042
H 3.266048 1.192371 1.327624
C 0.822593 6.064616 5.215285
H 0.265117 5.215440 5.591494
C 0.954218 7.228993 5.953285
H 0.494939 7.303494 6.930400
C 3.247586 2.080536 3.271238
H 2.564552 1.349045 3.713632
H 4.275182 1.780109 3.491823
C 1.678571 8.279599 5.413185
H 1.799521 9.204698 5.963950
H 1.037114 1.867329 1.814145
C 1.440896 2.793575 0.055864
H 2.043552 3.630792 -0.295814
H 1.703274 1.902181 -0.523757
H 0.389777 3.024746 -0.113927
